# Blood Urea Nitrogen-to-Albumin Ratio May Predict Mortality in Patients with Traumatic Brain Injury from the MIMIC Database: A Retrospective Study

**DOI:** 10.3390/bioengineering11010049

**Published:** 2024-01-02

**Authors:** Yiran Guo, Yuxin Leng, Chengjin Gao

**Affiliations:** 1 Department of Emergency, Xinhua Hospital, Shanghai Jiao Tong University School of Medicine, Shanghai 200092, China; guoyr-criticalcare@sjtu.edu.cn; 2 Critical Care Medicine Department, Peking University Third Hospital, Beijing 100191, China

**Keywords:** blood urea nitrogen-to-albumin ratio, traumatic brain injury, the MIMIC database, machine learning

## Abstract

Traumatic brain injury (TBI), a major global health burden, disrupts the neurological system due to accidents and other incidents. While the Glasgow coma scale (GCS) gauges neurological function, it falls short as the sole predictor of overall mortality in TBI patients. This highlights the need for comprehensive outcome prediction, considering not just neurological but also systemic factors. Existing approaches relying on newly developed biomolecules face challenges in clinical implementation. Therefore, we investigated the potential of readily available clinical indicators, like the blood urea nitrogen-to-albumin ratio (BAR), for improved mortality prediction in TBI. In this study, we investigated the significance of the BAR in predicting all-cause mortality in TBI patients. In terms of research methodologies, we gave preference to machine learning methods due to their exceptional performance in clinical support in recent years. Initially, we obtained data on TBI patients from the Medical Information Mart for Intensive Care database. A total of 2602 patients were included, of whom 2260 survived and 342 died in hospital. Subsequently, we performed data cleaning and utilized machine learning techniques to develop prediction models. We employed a ten-fold cross-validation method to obtain models with enhanced accuracy and area under the curve (AUC) (Light Gradient Boost Classifier accuracy, 0.905 ± 0.016, and AUC, 0.888; Extreme Gradient Boost Classifier accuracy, 0.903 ± 0.016, and AUC, 0.895; Gradient Boost Classifier accuracy, 0.898 ± 0.021, and AUC, 0.872). Simultaneously, we derived the importance ranking of the variable BAR among the included variables (in Light Gradient Boost Classifier, the BAR ranked fourth; in Extreme Gradient Boost Classifier, the BAR ranked sixth; in Gradient Boost Classifier, the BAR ranked fifth). To further evaluate the clinical utility of BAR, we divided patients into three groups based on their BAR values: Group 1 (BAR < 4.9 mg/g), Group 2 (BAR ≥ 4.9 and ≤10.5 mg/g), and Group 3 (BAR ≥ 10.5 mg/g). This stratification revealed significant differences in mortality across all time points: in-hospital mortality (7.61% vs. 15.16% vs. 31.63%), as well as one-month (8.51% vs. 17.46% vs. 36.39%), three-month (9.55% vs. 20.14% vs. 41.84%), and one-year mortality (11.57% vs. 23.76% vs. 46.60%). Building on this observation, we employed the Cox proportional hazards regression model to assess the impact of BAR segmentation on survival. Compared to Group 1, Groups 2 and 3 had significantly higher hazard ratios (95% confidence interval (CI)) for one-month mortality: 1.77 (1.37–2.30) and 3.17 (2.17–4.62), respectively. To further underscore the clinical potential of BAR as a standalone measure, we compared its performance to established clinical scores, like sequential organ failure assessment (SOFA), GCS, and acute physiology score III(APS-III), using receiver operator characteristic curve (ROC) analysis. Notably, the AUC values (95%CI) of the BAR were 0.67 (0.64–0.70), 0.68 (0.65–0.70), and 0.68 (0.65–0.70) for one-month mortality, three-month mortality, and one-year mortality. The AUC value of the SOFA did not significantly differ from that of the BAR. In conclusion, the BAR is a highly influential factor in predicting mortality in TBI patients and should be given careful consideration in future TBI prediction research. The blood urea nitrogen-to-albumin ratio may predict mortality in TBI patients.

## 1. Introduction

Traumatic brain injury (TBI) is a disruption in the brain and nervous system caused by an external force, like trauma [1]. It’s a global burden, affecting millions and leading to disability, mortality, and significant social and economic costs [2,3]. Although there have been studies on mortality predictive models and predictors in patients with TBI, most of them involved many variables and exhibited high complexity [4,5,6,], and few of them found strong and concise predictors for patients with TBI.

As a metabolic biomarker, the blood urea nitrogen-to-albumin ratio (BAR) is a simple yet comprehensive metabolic biomarker, reflecting nitrogen balance, kidney function, and overall metabolic status. It shows promise for predicting prognosis in patients undergoing major surgery [7] and those with sepsis [8]. However, its impact on TBI patient survival and its potential as a mortality predictor for them remain largely unexplored.

The Medical Information Mart for Intensive Care (MIMIC) III [9] and IV [10] databases are extensive critical care medicine databases, covering the electronic medical records from intensive care units at the Beth Israel Deaconess Medical Center (BIDMC) from 2001 to 2019. They include various baseline information, laboratory tests, vital signs, imaging information, diagnoses, operations, procedures (based on International Classification of Diseases codes (ICD codes)), and other rich clinical information. They are now widely used for research in critical care medicine, artificial intelligence, and other areas.

Machine learning, a form of computational learning, empowers computers to acquire knowledge from real-world data by employing specific rules and algorithms [11]. By analyzing statistics, it uncovers hidden patterns and relationships within complex datasets. In clinical medicine, machine learning methods can assist in clinical decision making [12,13]. Here, we focused on utilizing machine learning techniques and data mining on the MIMIC database to improve clinical work by generating valuable insights. Specifically, we investigated the influence of BAR on survival and its potential as a predictive tool for mortality in patients with TBI.

## 2. Materials and Methods

### 2.1. Data Source

The MIMIC-III/IV databases, built by the Massachusetts Institute of Technology and provided by BIDMC, are large-scale, retrospective resources for critical care research. Patient data were anonymized in accordance with the Health Insurance Portability and Accountability Act (HIPAA) Safe Harbor provision. This study utilized data retrieved from the MIMIC database (MIMIC-III: https://physionet.org/content/mimiciii/1.4/ (accessed on 25 December 2023); MIMIC-IV: https://physionet.org/content/mimiciv/2.2/ (accessed on 25 December 2023)).

### 2.2. Data Collection

Electronic health records from the MIMIC-III and -IV databases were extracted using structured query language based on the PostgreSQL service (version 13.7). The inclusion criteria comprised individuals with a TBI diagnosis and ICD-9/10 codes, and the Supplementary Materials list the diagnostic codes. Additionally, patients needed to be at least 18 years old, have a single admission to the hospital and intensive care unit (ICU), and have comprehensive data on admission albumin and urea nitrogen. The relevant data within 24 h of admission from the MIMIC database include: (1) demographic: age and sex; (2) vital signs: minimum average arterial pressure, maximum heart rate, maximum respiratory rate, minimum blood oxygen saturation, maximum temperature; (3) blood differential: maximum white blood cell, minimum hemoglobin, minimum hematocrit, minimum platelet, red blood cell distribution width (RDW); (4) electrolyte: maximum sodium, minimum potassium, maximum chloride, minimum calcium, phosphate, magnesium, maximum anion gap; (5) serum biochemicals: blood urea nitrogen-to-albumin ratio, maximum urea nitrogen, albumin, maximum creatinine, maximum glucose, minimum bicarbonate; (6) coagulation: maximum International normalized ratio (INR), prothrombin time (PT), and activated partial thromboplastin time (APTT); (7) scores: minimum Glasgow coma scale (GCS) score, acute physiology score III (APS-III), sequential organ failure assessment (SOFA) score; (8) comorbidity: Charlson comorbidity score, congestive heart failure, chronic pulmonary disease, rheumatic disease, renal disease, diabetes, liver disease; (9) type of trauma and surgery: intraparenchymal hemorrhage, extradural hemorrhage, subdural hemorrhage, subarachnoid hemorrhage, neurosurgery (the definition of neurosurgery see the National Healthcare Safety Network); (10) blood products infusion: first-day red blood cell infusion, first-day platelet infusion; (11) outcomes: one-month, three-month, and one-year mortality, length of stay in hospital and intensive care unit (ICU). All laboratory results were acquired within 24 h of admission. If an indicator was unavailable within the time frame, the first indicator obtained after admission was utilized as a substitute.

### 2.3. Statistical Analysis

Missing values were imputed using the K-nearest neighbor method [14] (R version 4.2.1 packages: DMwR2). The Pearson correlation coefficients (r values) were determined by the correlation tests (python 3.8.12) on continuous variables. Variables with r values greater than 0.8 were subsequently removed.

To assess BAR’s importance across multiple machine learning models, Python codes were utilized. The machine learning models with importance ranking function consisted of the following eight classifiers: Ada Boost Classifier, Decision Tree Classifier, Naive Bayes Classifier, Gradient Boost Classifier, Light Gradient Boost Classifier, Logistic regression Classifier, Random Forest Classifier, and Extreme Gradient Boost Classifier. Model performance was evaluated using accuracy, area under the curve (AUC), precision, recall rate, and F-score (the python packages and codes are shown in Supplementary Materials; Python version: 3.8.12, Jupyter Notebook version: 6.4.8).

The cut-off values for BAR were determined using the x-tile software [15] (X-Tile Software version 3.6.1 https://medicine.yale.edu/lab/rimm/research/software/ (accessed on 25 December 2023)) based on the Kaplan–Meier method. Shapiro–Wilk tests were utilized to evaluate variable conformity to a normal distribution. The Levene tests were utilized to evaluate homogeneity of variances. Normally distributed data are presented as mean ± standard deviation (SD), while non-normally distributed variables are reported as median and interquartile range (IQR). The Kruskal–Wallis tests were employed to determine significant differences between two groups of non-normally distributed continuous variables, while Student *t*-tests were used for two groups of normally distributed data. Wilcoxon tests assessed the statistical significance of differences among multiple heteroscedastic datasets, while analysis of variance tests were employed for homoscedastic data. Categorical variables were expressed as numbers and proportions, with significance determined using chi-squared or Fisher’s exact tests. Kaplan–Meier method and log-rank tests analyzed survival differences among groups (R version 4.2.1, function: aov, packages: car, tableone; SPSS 20.0).

Univariate and multivariate analyses were conducted utilizing the logistic and Cox risk proportion models (R version 4.2.1 function: glm, packages: survival). Factors with *p* < 0.05 and hazard ratios (HRs) ≤0.95 or ≥1.05 in univariate analysis were included in the multivariate analysis, with results reported as HRs, odds ratios (ORs), and 95% confidence intervals (CIs).

Receiver operating characteristic (ROC) curves were generated, and AUC values (R version 4.2.1 package qROC) were calculated to evaluate the predictive value of BAR.

## 3. Results

### 3.1. Patient Characteristics and Data Pre-Processing

**Figure 1:**
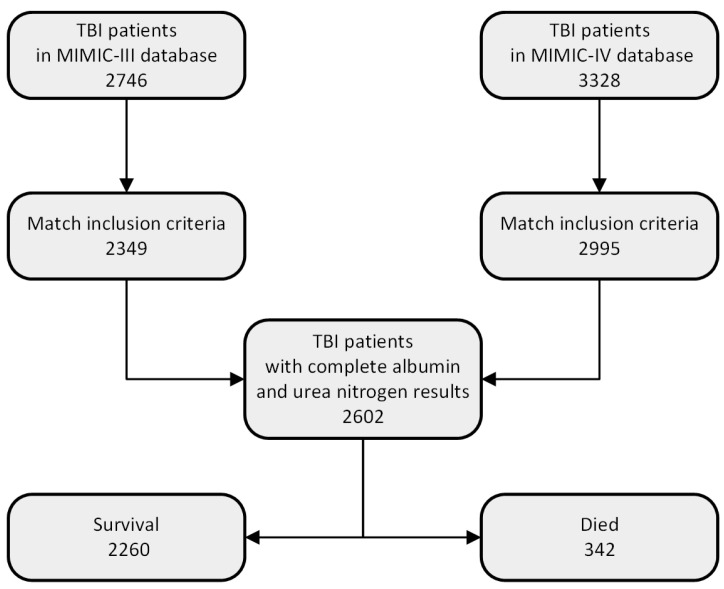
Flowchart showing inclusion and exclusion criteria.

Herein, we identified patients with TBI through initial screening using ICD-9/10 codes. A total of 2746 and 3328 patients were selected from the MIMIC-III and IV databases, respectively. A total of 2602 patients with TBI who satisfied the inclusion criteria were screened, with 2260 surviving and 342 experiencing in-hospital mortality, with one-year follow-up data being available (Figure 1).

The K-nearest neighbor method was used to insert the missing values. Then, we calculated r values by performing correlation tests on continuous variables and removing variables with r values above 0.8 (Hematocrit, INR) for further analysis. This resulted in a final set of 51 variables, including the outcome, for consideration in this study. Tables S1 and S2 present the missing rate of data, and a correlation matrix of continuous variables is shown in Supplementary S1.

Significant differences were observed between surviving and non-surviving patients across various characteristics, including age, sex, vital signs, blood counts, electrolytes, biochemicals, coagulation indexes, scores for different diseases, percentage of chronic diseases, and trauma types (Table 1).

### 3.2. BAR Importance Ranks High among All Included Factors Affecting In-Hospital Mortality in TBI Patients

The significance of the impact factor, and its contribution to our intended outcomes, can be quantified via methods of machine learning [16]. To explore the importance of BAR relative to other mortality-related factors, we utilized machine learning methods to construct predictive models using 43 variables as influential factors, with in-hospital mortality (0 for survival, 1 for death) as the outcome. A ten-fold cross-validation method [17] was employed to improve the efficacy of the models. The evaluation metrics of each model (accuracy, area under the curve (AUC), Recall Rate, Precision, and F1-Score) [18] are demonstrated in Table 2.

**Figure 2:**
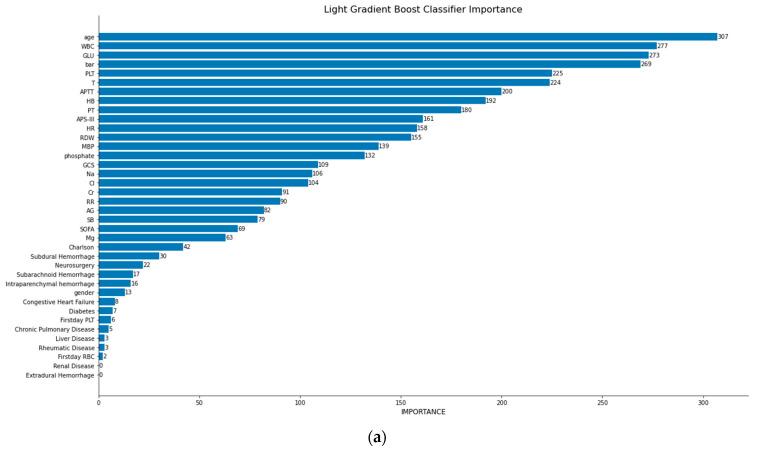

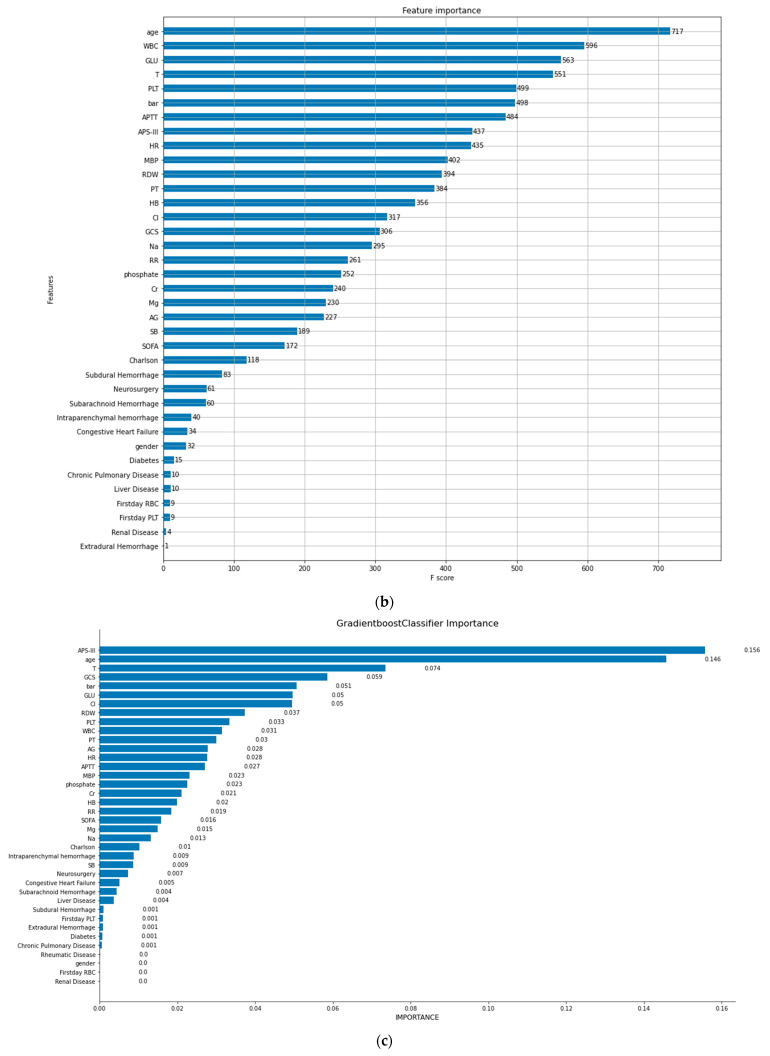
Three machine learning models and ranking of feature importance: (**a**) Light Gradient Boost Classifier model; (**b**) Extreme Gradient Boost (XGBoost) Classifier model; (**c**) Gradient Boost Classifier model. RDW, red blood cell distribution width; BAR, blood urea nitrogen-to-albumin ratio; INR, International normalized ratio; PT, prothrombin time; APTT, activated partial thromboplastin time; GCS, Glasgow coma scale; APS-III, acute physiology score III; SOFA, sequential organ failure assessment; RBC, red blood cell; PLT, platelet; MBP, average arterial pressure; T, temperature; RR, respiratory rate; HR, heart rate; AG, anion gap; SB, bicarbonate.

The top three models in terms of effectiveness, primarily judged by accuracy and AUC, were Light Gradient Boost Classifier, Extreme Gradient Boost (XGBoost) Classifier, and Gradient Boost Classifier. Their variable importance rankings are demonstrated in Figure 2. Among these three models, BAR ranked fourth, sixth, and fifth, respectively. Thus, the variable BAR holds significant importance among all the parameters impacting in-hospital mortality in patients with TBI (importance ranking and ROC curves of all models are shown in Figures S1~S16).

### 3.3. High-BAR Group Mortality Is Significantly Higher than That of Low-BAR Group

In order to enhance the significance of the relationship between BAR and the mortality rate, we employed a segmentation method [15] to group patients based on BAR levels. All patients were divided into three categories according to their BAR values: 0–4.9 mg/g, 4.9–10.5 mg/g, and greater than or equal to 10.5 mg/g (using x-tile software version 3.6.1). Significant differences were observed between these categories in various parameters, including vital signs (heart rate, mean blood pressure, respiratory rate, and oxygen saturation), various blood tests, and laboratory tests (blood glucose, white blood cells, red blood cells, platelets, coagulation indices). Additionally, differences were identified in trauma types (epidural hematoma, subdural hematoma, parenchymal brain hemorrhage), disease scores (SOFA, APSIII), mortality rate, and length of stay in hospital and ICU. BAR segmentation reveals a notable disparity in mortality among different groups (Table 3).

### 3.4. BAR-Increased Group of TBI Patients Has a Higher Death Risk

After categorizing the data according to the BAR groups, we aimed to further explore the potential risk of increased BAR on the mortality of patients. Herein, we utilized the Cox risk-proportional regression model to assess the relationship between BAR and survival in TBI patients. In univariate Cox analysis, higher-value BAR segments were associated with higher one-month mortality compared to lower-value segments (HR_21_ 95%CI 2.13 (1.68–2.70), HR_31_ 95%CI 4.90 (3.77–6.38), *p* < 0.001), and the BAR segments were independently associated with higher one-month mortality in multivariate analysis (HR_21_ 95%CI 1.77 (1.37–2.30), HR_31_ 95%CI 3.17 (2.17–4.62), *p* < 0.001) (Table 4). Similar patterns were observed for the relationship between BAR and three-month and one-year mortality, with *p*-values less than 0.01 in both cases (Tables S3 and S4).

**Figure 3:**
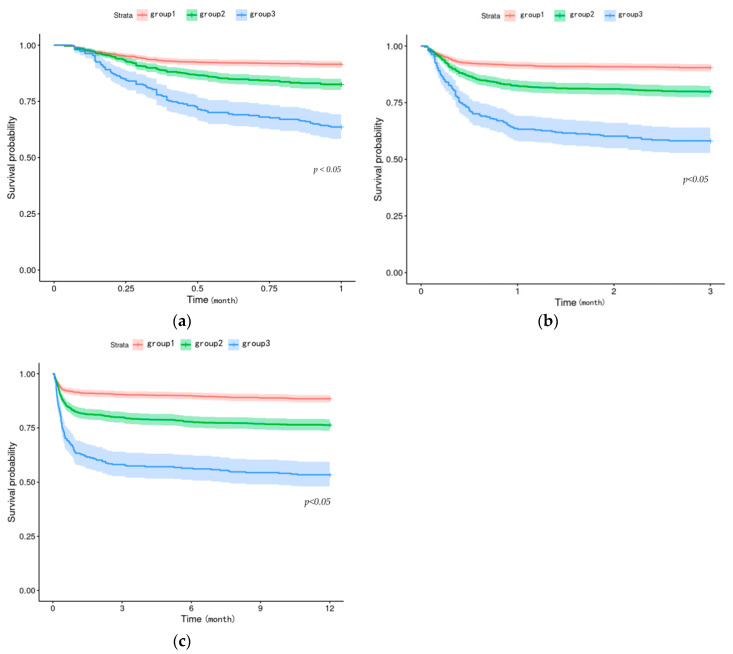
The relationship between BAR and all-cause mortality in patients with TBI was described using Kaplan-Meier survival curves to characterize the survival probability for patients with different BAR levels, *p*-values were acquired using the log-rank test, and graphs were drawn to show (**a**) survival curves for patients at one month; (**b**) three months; (**c**) one year.

The Kaplan–Meier curves illustrated the relationship between BAR levels and survival in TBI patients, demonstrating that higher BAR levels were associated with higher mortality rates in TBI patients (Figure 3).

To further corroborate our findings, we conducted a stepwise logistic regression analysis to examine the association between BAR and in-hospital mortality in patients with TBI. Consistent with our previous analysis, elevated BAR levels were significantly associated with an increased risk of all-cause mortality (*p* < 0.01) (Table S5). This reinforces the link between higher BAR and negative outcomes in TBI patients.

### 3.5. Prognostic Effectiveness of BAR Is as Good as SOFA Score in TBI Patients

**Figure 4:**
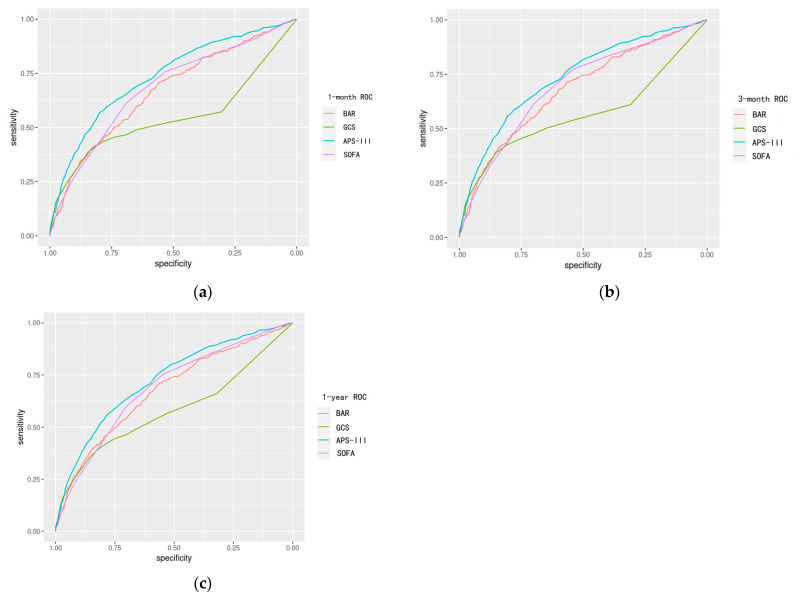
The receiver operating characteristic (ROC) curves were used to assess the predictive value of BAR and compare them to other predictors. ROC curve for (**a**) 1-month; (**b**) 3-month and (**c**) 1-year all-cause mortality; APS-III, acute physiology score III; SOFA, sequential organ failure assessment; GCS, Glasgow coma scale; BAR, Blood Urea Nitrogen-to-Albumin Ratio.

Finally, we evaluated the prognostic value of BAR compared to traditional clinical scores. Through ROC curve analysis, we assessed BAR’s predictive performance for one-month, three-month, and one-year mortality, with AUC values (95%CI) of 0.67 (0.64–0.70), 0.68 (0.65–0.70), and 0.68 (0.65–0.70), respectively. Notably, the AUC value of SOFA did not significantly differ from that of BAR (Figure 4, Table S6). This suggests that BAR demonstrates comparable prognostic value to SOFA in TBI patients.

## 4. Discussion

This study establishes a robust and independent association between BAR and both mortality and survival in patients with TBI. Currently, few succinct, easily accessible, and influential predictors of TBI patient’s outcomes exist, with most relying on complex scoring systems or numerous factors. Herein, we found statistically significant HRs and ORs for BAR segments, demonstrating their independent relationship with one-month, three-month, and one-year mortality. Notably, BAR also demonstrated superior performance as a feature in the Light Gradient Boost Classifier, Extreme Gradient Boost Classifier, and Gradient Boost Classifier, ranking fourth, sixth, and fifth and proving marginally more significant than the APS-III, GCS, and SOFA score in the Light Gradient Boost Classifier and Extreme Gradient Boost Classifier, proving more significant than the GCS and SOFA score in the Gradient Boost model. The other factors ranked ahead of the model, including age, temperature, blood glucose, platelets, white blood cells, APS-III score, were not as clinically valuable or succinct as BAR. Comparable levels of predicting effectiveness are achieved by BAR and SOFA based on ROC curves. From various perspectives, various models developed in this study demonstrate that BAR is a robust predictor of survival and mortality in TBI patients.

Albumin, a liver-synthesized protein belonging to the globulin family, reflects both nutritional status and liver function [19,20,21]. Reduced serum albumin directly lowers plasma colloid osmotic pressure, causing fluid leakage into tissue [22]. Patients in acute, critical, and high-nutritional-risk conditions are more likely to experience hypoproteinemia [23], which signifies a worse prognosis. In severe trauma, with massive wound exudation and stress, albumin levels are more likely to be drastically reduced, indicating a poor outcome. Urea nitrogen, a protein–amino acid metabolite excreted by the kidneys, increases in situations of increased catabolism, hemorrhage, or renal dysfunction [24]. This elevation may occur in patients with severe TBI. Urea nitrogen has been used as a predictor in various critical illnesses [25,26], including ischemic stroke [27], acute myocardial infarction [28], and cardiogenic shock [29]. Herein, the BAR further amplifies the predictive value of urea nitrogen and albumin in trauma as well as acute and critical illnesses [30]. Furthermore, BAR has been demonstrated as a reliable predictor of mortality in elderly emergency patients [31] and post-surgical patients [7,32]. Additionally, its efficacy in forecasting the prognosis of patients undergoing cardiac transplants [33], those affected by COVID-19 [34], and individuals with acute ischemic stroke [35], has been confirmed. The BAR is comparable to the Acute Physiology and Chronic Health Evaluation-II and SOFA scores when applied to patients with sepsis [36], and it is more concise and easily accessible than these intricate scores.

Informed by relevant literature [37], we selected specific machine learning models based on their suitability for importance ranking: Light Gradient Boost Classifier, Extreme Gradient Boost Classifier, Gradient Boost Classifier, Random Forest Classifier, Ada Boost Classifier, Logistic Regression Classifier, Decision Tree Classifier, and Naive Bayes Classifier. Our results indicate that the Gradient boost family of models achieved slightly better performance. The Gradient Boost model operates by sequentially generating multiple weak learners. Each weak learner aims to fit the negative gradient of the loss function of the previous cumulative model. This process ensures that the loss of the cumulative model decreases in the direction of the negative gradient when the weak learner is added. The Boosted Tree program’s capacity to rapidly assess potential predictability, along with its robustness, renders it a valuable preprocessing tool for handling imbalanced data [16,38]. As for the Light Gradient Boost Model, the best performer in our results, it significantly outperforms the Extreme Gradient Boost Model in terms of computational speed and memory consumption [39].

As for regression models, we usually utilize established functions to apply models to the data, but the accuracy of the fit is not always guaranteed. Logistic regression was also represented in the results of this work. The decision between regression and machine learning is primarily determined by our intended objective [40]. For purely predictive purposes, prioritizing model accuracy often leads us toward machine learning approaches. However, if we are specifically interested in understanding the impact of a variable and conducting a descriptive study, estimating HRs or ORs to assess survival and mortality risks, then Cox proportional hazard regression becomes a more suitable option.

All information used in this analysis was extracted from the MIMIC database, which is susceptible to selection bias. Additionally, while the Cox model used in this study assumes a constant HR throughout the disease progression, it is important to acknowledge that HRs can be dynamic [41]. Consequently, the HR of BAR can only serve as a reference and trend, with less precision compared to machine learning approaches. However, the machine learning approach is a classifier that solely considers mortality as an outcome variable, rendering it incapable of reflecting long-term survival. Furthermore, severe traumatic complications, such as traumatic coagulopathy, systemic inflammatory response syndrome (SIRS), and acute respiratory distress syndrome (ARDS), as well as infections and sepsis that may develop in conjunction with the disease’s progression, were excluded from this study. Integrating these complications into future models becomes an important avenue for future research.

## 5. Conclusions

We first constructed prediction models to obtain the importance ranking of BAR for the outcome. And, then, we explored how mortality and death risk change with rising BAR stages. Finally, we compared ROC curves to evaluate the independent predictive value of BAR with mainstream disease severity scores. The following conclusions are drawn:

1. Among all considered variables, BAR stands out as highly important, significantly contributing to the mortality rates of patients with TBI.
2. Stratifying patients by BAR levels reveals a marked disparity in mortality across the strata, with individuals in the high-BAR group facing a considerably higher risk of all-cause mortality compared to those in the low-BAR group.
3. For predicting all-cause mortality in TBI patients, BAR outperforms the GCS score, performs comparably to the SOFA score, and falls slightly behind the APS-III score in independent predictive ability.
4. The BAR ratio emerges as a potentially strong predictor of mortality in patients with TBI.

## Figures and Tables

**Table 1:** Baseline characteristics in patients with traumatic brain injury.

Characteristic	Survival (2260)	Died (342)	*p*
Age (years)	61.53 [44.05, 78.68]	72.02 [56.59, 84.66]	<0.001
Male sex (%)	1497 (66.24)	206 (60.23)	0.0344
Average arterial pressure(mmHg)	62.00 [55.00, 70.00]	58.00 [49.00, 65.00]	<0.001
Heart rate (beats/min)	101.00 [89.00, 115.00]	104.00 [90.25, 123.75]	0.0031
Respiratory rate (beats/min)	25.00 [22.00, 28.00]	26.00 [23.00, 30.00]	<0.001
Blood oxygen saturation (%)	94.00 [92.00, 96.00]	95.00 [92.00, 97.75]	0.0285
Temperature (°C)	37.61 [37.17, 38.11]	37.94 [37.38, 38.61]	<0.001
White blood cell (10^9^/L)	12.40 [9.10, 16.50]	14.40 [9.93, 19.45]	<0.001
Hemoglobin (10^12^/L)	11.10 [9.50, 12.40]	9.75 [8.33, 11.08]	<0.001
Hematocrit (%)	32.30 [27.90, 36.40]	28.85 [24.43, 32.30]	<0.001
Platelet (10^9^/L)	188.00 [140.00, 235.00]	159.50 [114.50, 213.00]	<0.001
RDW (%)	13.80 [13.10, 14.70]	14.40 [13.50, 15.80]	<0.001
Sodium (mmol/L)	141.00 [139.00, 143.00]	142.00 [140.00, 146.00]	<0.001
Potassium (mmol/L)	3.70 [3.40, 4.00]	3.60 [3.20, 4.00]	0.0021
Chloride (mmol/L)	106.00 [103.00, 110.00]	109.00 [104.00, 113.00]	<0.001
Calcium (mmol/L)	8.30 [7.80, 8.80]	8.20 [7.50, 8.70]	0.0068
Phosphate (mmol/L)	3.30 [2.80, 3.80]	3.40 [2.80, 4.10]	0.0258
Magnesium (mmol/L)	1.90 [1.60, 2.10]	2.00 [1.70, 2.10]	<0.001
Anion gap (mmol/L)	16.00 [14.00, 18.00]	17.00 [15.00, 20.00]	<0.001
BAR	4.62 [3.24, 6.69]	6.36 [4.38, 10.68]	<0.001
Urea nitrogen (mg/dL)	16.00 [12.00, 22.00]	21.00 [14.00, 33.00]	<0.001
Albumin (g/dL)	3.50 [3.10, 3.90]	3.20 [2.80, 3.70]	<0.001
Creatinine (mg/dL)	0.90 [0.70, 1.20]	1.10 [0.80, 1.60]	<0.001
Bicarbonate (mmol/L)	23.00 [20.00, 25.00]	21.00 [18.00, 24.00]	<0.001
Glucose (mmol/L)	151.00 [124.00, 186.00]	182.00 [150.00, 233.00]	<0.001
INR	1.20 [1.10, 1.30]	1.30 [1.10, 1.70]	<0.001
PT (s)	13.32 [12.40, 14.80]	14.30 [13.03, 17.88]	<0.001
APTT (s)	28.00 [25.50, 31.60]	30.30 [26.23, 35.98]	<0.001
GCS score	14.00 [10.00, 15.00]	13.50 [7.00, 15.00]	0.1367
APS-III score	36.00 [28.00, 47.00]	54.50 [39.00, 73.00]	<0.001
SOFA score	3.00 [2.00, 5.00]	5.00 [4.00, 8.00]	<0.001
Charlson	4.00 [1.00, 5.00]	5.00 [3.00, 6.00]	<0.001
Congestive heart failure	269 (11.90%)	85 (24.85%)	<0.001
Chronic pulmonary disease	259 (11.46%)	36 (10.53%)	0.6773
Rheumatic disease	39 (1.73%)	8 (2.34%)	0.5645
Renal disease	179 (7.92%)	48 (14.04%)	<0.001
Diabetes	392 (17.35%)	88 (25.73%)	<0.001
Liver disease	175 (7.74%)	36 (10.53%)	0.0988
Intraparenchymal hemorrhage	174 (7.70%)	43 (12.57%)	0.0034
Extradural hemorrhage	40 (1.77%)	8 (2.34%)	0.6076
Subdural hemorrhage	835 (36.95%)	129 (37.72%)	0.8293
Subarachnoid hemorrhage	462 (20.44%)	91 (26.61%)	0.0115
Neurosurgery	617 (27.30%)	130 (38.01%)	<0.001
First-day RBC infusion	288 (12.74%)	83 (24.27%)	<0.001
First-day PLT infusion	207 (9.16%)	59 (17.25%)	<0.001

Variable represented as the median (quartiles) or the number of patients (%). RDW, red blood cell distribution width; BAR, blood urea nitrogen-to-albumin ratio; INR, International normalized ratio; PT, prothrombin time; APTT, activated partial thromboplastin time; GCS, Glasgow coma scale; APS-III, acute physiology score III; SOFA, sequential organ failure assessment; RBC, red blood cell; PLT, platelet.

**Table 2:** Eight machine learning models of in-hospital mortality and their evaluation metrics.

Model	Accuracy	AUC	F Score	Recall Rate	Precision
	(Mean ± SD)				
Light Gradient Boost Classifier	0.905 ± 0.016	0.888	0.560	0.459	0.717
Extreme Gradient Boost Classifier	0.903 ± 0.016	0.895	0.532	0.421	0.724
Gradient Boost Classifier	0.898 ± 0.021	0.872	0.536	0.447	0.668
Random Forest Classifier	0.894 ± 0.008	0.892	0.361	0.228	0.867
Ada Boost Classifier	0.877 ± 0.011	0.817	0.441	0.368	0.550
Logistic Regression Classifier	0.873 ± 0.008	0.756	0.206	0.126	0.573
Decision Tree Classifier	0.847 ± 0.016	0.656	0.405	0.398	0.413
Naive Bayes Classifier	0.806 ± 0.018	0.755	0.372	0.439	0.323

AUC: area under the curve.

**Table 3:** Patient characteristics after BAR stratification.

BAR Level	<4.9 1340	4.9~10.5 968	≥10.5 294	*p*
Age (years)	52.61 [37.11, 66.94]	73.93 [56.33, 84.01]	77.70 [66.47, 85.63]	<0.001
Male sex (%)	895 (66.79%)	613 (63.33%)	195 (66.33%)	0.213
Average arterial pressure(mmHg)	64.00 [57.00, 71.00]	60.00 [52.00, 67.00]	57.00 [49.00, 63.00]	<0.001
Heart rate (beats/min)	103.00 [92.00, 117.00]	98.00 [86.00, 114.00]	101.00 [87.00, 114.00]	<0.001
Respiratory rate (beats/min)	25.00 [22.00, 28.00]	25.00 [22.00, 29.00]	27.00 [23.00, 31.00]	<0.001
Blood oxygen saturation (%)	95.00 [92.00, 97.00]	94.00 [91.00, 96.00]	93.00 [90.00, 95.75]	<0.001
Temperature (°C)	37.72 [37.22, 38.22]	37.61 [37.17, 38.11]	37.42 [37.06, 38.00]	<0.001
White blood cell (10^9^/L)	12.40 [9.10, 16.70]	12.90 [9.50, 16.80]	12.80 [8.83, 17.78]	0.092
Hemoglobin (10^12^/L)	11.50 [10.10, 12.90]	10.40 [8.90, 11.80]	9.20 [8.10, 10.40]	<0.001
Hematocrit (%)	33.50 [29.40, 37.40]	30.40 [26.10, 34.93]	27.60 [24.35, 31.50]	<0.001
Platelet (10^9^/L)	196.00 [149.00, 243.00]	173.50 [126.00, 222.00]	151.50 [106.25, 207.75]	<0.001
RDW (%)	13.60 [13.00, 14.43]	13.90 [13.20, 15.00]	15.25 [14.20, 16.70]	<0.001
Sodium (mmol/L)	141.00 [138.00, 143.00]	141.00 [139.00, 143.00]	142.00 [139.00, 145.00]	0.027
Potassium (mmol/L)	3.60 [3.30, 3.90]	3.70 [3.40, 4.10]	3.90 [3.50, 4.40]	<0.001
Chloride (mmol/L)	106.00 [103.00, 109.00]	107.00 [103.00, 111.00]	107.50 [102.00, 112.00]	<0.001
Calcium (mmol/L)	8.30 [7.80, 8.80]	8.30 [7.70, 8.80]	8.20 [7.40, 8.80]	0.162
Phosphate (mmol/L)	3.20 [2.70, 3.70]	3.30 [2.80, 3.80]	3.85 [3.20, 5.00]	<0.001
Magnesium (mmol/L)	1.80 [1.60, 2.00]	1.90 [1.70, 2.10]	2.10 [1.80, 2.28]	<0.001
Anion gap (mmol/L)	16.00 [14.00, 18.00]	15.00 [13.00, 18.00]	18.00 [16.00, 21.00]	<0.001
Urea nitrogen (mg/dL)	12.00 [10.00, 15.00]	22.00 [18.75, 25.00]	46.00 [37.00, 58.00]	<0.001
Albumin (g/dL)	3.70 [3.40, 4.10]	3.30 [2.88, 3.70]	3.10 [2.70, 3.48]	<0.001
Creatinine (mg/dL)	0.80 [0.70, 1.00]	1.00 [0.90, 1.30]	2.00 [1.50, 2.88]	<0.001
Bicarbonate (mmol/L)	23.00 [21.00, 25.00]	23.00 [20.00, 25.00]	21.00 [18.00, 24.00]	<0.001
Glucose (mmol/L)	144.00 [121.00, 173.25]	166.00 [137.00, 203.25]	182.00 [143.25, 235.00]	<0.001
INR	1.20 [1.10, 1.30]	1.20 [1.10, 1.50]	1.30 [1.10, 1.80]	<0.001
PT (s)	13.15 [12.30, 14.30]	13.70 [12.70, 15.88]	14.50 [12.90, 18.78]	<0.001
APTT (s)	27.60 [25.40, 30.80]	28.60 [25.88, 32.90]	30.85 [26.80, 35.75]	<0.001
GCS score	14.00 [10.00, 15.00]	14.00 [9.00, 15.00]	14.00 [9.00, 15.00]	0.533
APS-III score	32.00 [24.00, 42.00]	41.50 [33.00, 55.00]	56.00 [45.00, 69.00]	<0.001
SOFA score	3.00 [2.00, 4.00]	4.00 [2.00, 6.00]	6.00 [4.00, 8.00]	<0.001
Charlson score	2.00 [1.00, 4.00]	4.00 [3.00, 6.00]	6.00 [5.00, 8.00]	<0.001
Congestive heart failure	63 (4.70%)	167 (17.25%)	124 (42.18%)	<0.001
Chronic pulmonary disease	111 (8.28%)	137 (14.15%)	47 (15.99%)	<0.001
Rheumatic disease	19 (1.42%)	18 (1.86%)	10 (3.40%)	0.068
Renal disease	19 (1.42%)	75 (7.75%)	133 (45.24%)	<0.001
Diabetes	155 (11.57%)	208 (21.49%)	117 (39.80%)	<0.001
Liver disease	95 (7.09%)	70 (7.23%)	46 (15.65%)	<0.001
Intraparenchymal hemorrhage	98 (7.31%)	91 (9.40%)	28 (9.52%)	0.149
Extradural hemorrhage	31 (2.31%)	13 (1.34%)	4 (1.36%)	0.187
Subdural hemorrhage	440 (32.84%)	394 (40.70%)	130 (44.22%)	<0.001
Subarachnoid hemorrhage	245 (18.28%)	228 (23.55%)	80 (27.21%)	<0.001
Neurosurgery	401 (29.93%)	292 (30.17%)	54 (18.37%)	<0.001
First-day RBC infusion	107 (7.99%)	195 (20.14%)	69 (23.47%)	<0.001
First-day PLT infusion	94 (7.01%)	125 (12.91%)	47 (15.99%)	<0.001
Mortality	102 (7.61%)	147 (15.19%)	93 (31.63%)	<0.001
Length of stay in ICU	2.56 [1.40, 5.81]	3.69 [1.77, 8.74]	3.56 [1.77, 7.84]	<0.001
Length of stay in hospital	7.43 [4.05, 14.71]	9.52 [5.27, 18.14]	9.49 [5.55, 16.75]	<0.001
1-month mortality	114 (8.51%)	169 (17.46%)	107 (36.39%)	<0.001
3-month mortality	128 (9.55%)	195 (20.14%)	123 (41.84%)	<0.001
1-year mortality	155 (11.57%)	230 (23.76%)	137 (46.60%)	<0.001

Variable represented as the median (quartiles) or the number of patients (%). RDW, red blood cell distribution width; BAR, blood urea nitrogen-to-albumin ratio; INR, International normalized ratio; PT, prothrombin time; APTT, activated partial thromboplastin time; GCS, Glasgow coma scale; APS-III, acute physiology score III; SOFA, sequential organ failure assessment; RBC, red blood cell; PLT, platelet.

**Table 4:** Univariate and multivariate Cox risk-proportional model.

Characteristic	Univariate Model		Multivariate Model	
	HR 95%CI	*p*	HR 95%CI	*p*
Age (years)	1.00 (1.00–1.01)	<0.001		
Male sex (%)	0.82 (0.67–1.00)	0.050	0.82 (0.66–1.03)	0.088
Average arterial pressure(mmHg)	0.98 (0.97–0.98)	<0.001	Not selected	
Heart rate (beats/min)	1.01 (1.00–1.01)	<0.001	Not selected	
Respiratory rate (beats/min)	1.04 (1.02–1.06)	<0.001	Not selected	
Blood oxygen saturation (%)	1.00 (0.98–1.01)	0.733	-	
Temperature (°C)	1.45 (1.27–1.66)	<0.001	1.42 (1.24–1.64)	<0.001
White blood cell (10^9^/L)	1.00 (1.00–1.00)	<0.001	Not selected	
Hemoglobin (10^12^/L)	0.81 (0.77–0.84)	<0.001	0.92 (0.86–0.97)	0.005
Platelet (10^9^/L)	1.00 (1.00–1.00)	<0.001	Not selected	
RDW (%)	1.17 (1.12–1.22)	<0.001	1.04 (0.98–1.11)	0.176
Sodium (mmol/L)	1.10 (1.08–1.12)	<0.001	1.11 (1.07–1.15)	<0.001
Potassium (mmol/L)	0.90 (0.74–1.10)	0.302	-	
Chloride (mmol/L)	1.06 (1.05–1.08)	<0.001	0.95 (0.92–0.98)	0.001
Calcium (mmol/L)	0.93 (0.83–1.04)	0.199	-	
Phosphate (mmol/L)	1.21 (1.11–1.32)	<0.001	0.97 (0.88–1.08)	0.597
Magnesium (mmol/L)	1.76 (1.33–2.33)	<0.001	1.71 (1.28–2.27)	<0.001
Anion gap (mmol/L)	1.07 (1.05–1.09)	<0.001	0.98 (0.95–1.01)	0.145
Creatinine (mg/dL)	1.14 (1.07–1.22)	<0.001	0.83 (0.72–0.95)	0.008
Bicarbonate (mmol/L)	0.91 (0.89–0.93)	<0.001	0.91 (0.87–0.95)	<0.001
Glucose (mmol/L)	1.00 (1.00–1.00)	<0.001	Not selected	
PT (s)	1.01 (1.01–1.02)	<0.001	Not selected	
APTT (s)	1.01 (1.01–1.02)	<0.001	Not selected	
GCS score	0.89 (0.87–0.92)	<0.001	0.94 (0.91–0.97)	<0.001
APS-III score	1.03 (1.03–1.03)	<0.001	Not selected	
SOFA score	1.18 (1.15–1.21)	<0.001	1.03 (0.98–1.08)	0.207
Charlson	1.13 (1.1–1.17)	<0.001	1.07 (1.02–1.13)	0.009
Congestive heart failure	2.16 (1.72–2.72)	<0.001	1.28 (0.95–1.71)	0.103
Chronic pulmonary disease	0.99 (0.73–1.35)	0.951	-	
Rheumatic disease	1.31 (0.68–2.53)	0.428	-	
Renal disease	2.03 (1.55–2.66)	<0.001	0.82 (0.55–1.22)	0.332
Diabetes	1.57 (1.25–1.97)	<0.001	1.00 (0.76–1.31)	0.998
Liver disease	1.19 (0.85–1.67)	0.310	-	
Intraparenchymal hemorrhage	1.52 (1.12–2.07)	0.008	1.54 (1.13–2.11)	0.007
Extradural hemorrhage	1.31 (0.67–2.53)	0.429	-	
Subdural hemorrhage	1.00 (0.81–1.23)	0.989	-	
Subarachnoid hemorrhage	1.35 (1.08–1.69)	0.009	1.26 (0.99–1.60)	0.057
Neurosurgery	1.40 (1.13–1.72)	0.002	1.41 (1.13–1.78)	0.003
First-day RBC infusion	2.04 (1.61–2.57)	<0.001	1.03 (0.76–1.40)	0.853
First-day PLT infusion	1.76 (1.34–2.30)	<0.001	0.96 (0.70–1.31)	0.795
BAR group1	Reference	-	Reference	-
BAR group2	2.13 (1.68–2.70)	<0.001	1.77 (1.37–2.30)	<0.001
BAR group3	4.90 (3.77–6.38)	<0.001	3.17 (2.17–4.62)	<0.001

Cox risk-proportional model results represented as the HR (95%CI). RDW, red blood cell distribution width; BAR, blood urea nitrogen-to-albumin ratio; INR, International normalized ratio; PT, prothrombin time; APTT, activated partial thromboplastin time; GCS, Glasgow coma scale; APS-III, acute physiology score III; SOFA, sequential organ failure assessment; RBC, red blood cell; PLT, platelet.

## Data Availability

Data in the study come from MIMIC-III and MIMIC-IV databases; MIMIC-III: https://physionet.org/content/mimiciii/1.4/ (accessed on 25 December 2023); MIMIC-IV: https://physionet.org/content/mimiciv/2.2/ (accessed on 25 December 2023).

## Supplementary Materials

The following supporting information can be downloaded at: https://www.mdpi.com/article/10.3390/bioengineering11010049/s1, Table S1: Missing value of data from the MIMIC-III database; Table S2: Missing value of data from the MIMIC-IV database; Table S3: Cox regression analysis of three month; Table S4: Cox regression analysis of one year; Table S5: Logistic regression analysis; Table S6: AUC of ROC; Along with the ICD codes used in the study; Tables S7–S14: Machine learning model efficacy; Figures S1~S8: Machine learning feature importance; Figures S9~S16: ROC curves of machine learning classifier; Along with python codes, structured query language (SQL) codes used in the study. Supplementary S1: A correlation matrix of continuous variables (correlation martix.xlsx).
