# Supplementary material for: Blood Urea Nitrogen-to-Albumin Ratio May Predict Mortality in Patients with Traumatic Brain Injury from the MIMIC Database: A Retrospective Study
Source: Bioengineering (Basel). 2024 Jan 2;11(1):49. doi: 10.3390/bioengineering11010049 (PMC10812946; doi:10.3390/bioengineering11010049)
Supplement: Supplementary file 1 [file bioengineering-11-00049-s001.zip › bioengineering-2740069-supplementary/Supplemental Materials.pdf]

Contents

Diagnosis of traumatic brain injury......2

ICD Codes for neurosurgical procedures......6

Missing value of data from the MIMIC-III database table S1......9

Missing value of data from the MIMIC-IV database table S2......11

Cox regression analysis of three month table S3......13

Cox regression analysis of one year table S4......14

Logistic regression analysis table S5......15

AUC of ROC table S6......16

Machine learning Model Efficacy tables S7~S14......17

Machine Learning feature importance Figures S1~S8......20

ROC curves of machine learning classifier Figures S9~S16......24

Python codes......28

SQL codes......41

Diagnosis of traumatic brain injury

The TRAUMATIC BRAIN INJURY (TBI) is from ‘DoD Standard Surveillance Case Definition for TBI Adapted for AFHSB Use’
the icd codes are:
--TRAUMATIC BRAIN INJURY (TBI)
--DoD Standard Surveillance Case Definition for TBI Adapted for AFHSB Use
--In the U.S. Military Health System (MHS), traumatic brain injury (TBI) is defined as “traumatically
--induced structural injury and/or physiological disruption of brain function as a result of an external
--force that is indicated by new onset or worsening of at least one of the following clinical signs,
--immediately following the event: any period of loss of or decreased level of consciousness; any loss of
--memory for events immediately before or after the injury; any alteration in mental state at the time of
--the injury (confusion, disorientation, slowed thinking, etc.); neurological deficits (weakness, loss of
--balance, change in vision, praxis, paresis/plegia, sensory loss, aphasia, etc.) that may or may not be
--transient; intracranial lesion.”

'F0781' --Other Personality and Behavioral Disorders Due to Known Physiological Condition
,'S0402XA' --Injury to Optic Chiasm and Optic Tract
,'S04031A','S04032A','S04039A'
,'S04041A','S04042A','S04049A'
,'S060X0A','S060X1A','S060X9A' --Concussion
,'S061X0A','S061X1A','S061X2A','S061X3A','S061X4A','S061X5A','S061X6A','S061X7A','S061X8A','S061X9A' --Traumatic Cerebral Edema
,'S062X0A','S062X1A','S062X2A','S062X3A','S062X4A','S062X5A','S062X6A','S062X7A','S062X8A','S062X9A' --Diffuse Traumatic Brain Injury
,'S06300A','S06301A','S06302A','S06303A','S06304A','S06305A','S06306A','S06307A','S06308A','S06309A' --unspecified focal traumatic brain injury
,'S06310A','S06311A','S06312A','S06313A','S06314A','S06315A','S06316A','S06317A','S06318A','S06319A' --contusion and laceration of right cerebrum
,'S06320A','S06321A','S06322A','S06323A','S06324A','S06325A','S06326A','S06327A','S06328A','S06329A' --contusion and laceration of left cerebrum
,'S06330A','S06331A','S06332A','S06333A','S06334A','S06335A','S06336A','S06337A','S06338A','S06339A' --contusion and laceration of cerebrum, unspecified
,'S06340A','S06341A','S06342A','S06343A','S06344A','S06345A','S06346A','S06347A','S06348A','S06349A' --traumatic hemorrhage of right cerebrum
,'S06350A','S06351A','S06352A','S06353A','S06354A','S06355A','S06356A','S06357A','S06358A','S

06359A' --traumatic hemorrhage of left cerebrum
,'S06360A','S06361A','S06362A','S06363A','S06364A','S06365A','S06366A','S06367A','S06368A','S06369A' --traumatic hemorrhage of cerebrum, unspecified
,'S06370A','S06371A','S06372A','S06373A','S06374A','S06375A','S06376A','S06377A','S06378A','S06379A' --contusion, laceration, and hemorrhage of cerebellum
,'S06380A','S06381A','S06382A','S06383A','S06384A','S06385A','S06386A','S06387A','S06388A','S06389A' --contusion, laceration, and hemorrhage of brainstem
,'S064X0A','S064X1A','S064X2A','S064X3A','S064X4A','S064X5A','S064X6A','S064X7A','S064X8A','S064X9A' --epidural hemorrhage
,'S065X0A','S065X1A','S065X2A','S065X3A','S065X4A','S065X5A','S065X6A','S065X7A','S065X8A','S065X9A' --traumatic subdural hemorrhage
,'S066X0A','S066X1A','S066X2A','S066X3A','S066X4A','S066X5A','S066X6A','S066X7A','S066X8A','S066X9A' --traumatic subarachnoid hemorrhage
,'S06890A','S06891A','S06892A','S06893A','S06894A','S06895A','S06896A','S06897A','S06898A','S06899A' --other specified intracranial injury
,'S069X0A','S069X1A','S069X2A','S069X3A','S069X4A','S069X5A','S069X6A','S069X7A','S069X8A','S069X9A','S069X9S' --unspecified intracranial injury
,'S020XXA','S020XXB' --Fracture of Vault of Skull
,'S02101A','S02102A','S02109A','S02101B','S02102B','S02109B' --Fracture of Base of Skull
,'S02110A','S0211AA','S0211BA','S02110B','S0211AB','S0211BB' --type I occipital condyle fracture
,'S02111A','S0211CA','S0211DA','S02111B','S0211CB','S0211DB' --type II occipital condyle fracture
,'S02112A','S0211EA','S0211FA','S02112B','S0211EB','S0211FB' --type III occipital condyle fracture
,'S02113A','S02113B' --unspecified occipital condyle fracture
,'S02118A','S0211GA','S0211HA','S02118B','S0211GB','S0211HB' --other fracture of occiput
,'S02119A','S02119B' --unspecified fracture of occiput
,'S0219XA','S0219XB' --other fracture of base of skull
,'S0280XA','S0281XA','S0282XA','S0280XB','S0281XB','S0282XB'--Fracture of Other Specified Skull and Facial Bones
,'S0291XA','S0291XB' --Fracture of Unspecified Skull and Facial Bones
,'S071XXA' --crushing injury of skull, initial encounter

'3102' --Other Personality and Behavioral Disorders due to Known Physiological Condition
,'8500','8502','8503','8504','8505','8509' --Concussion
,'85011','85012'
,'85100','85101','85102','85103','85104','85105','85106','85109' --Cerebral Laceration and Contusion Without Mention of Open Intracranial Wound
,'85120','85121','85122','85123','85124','85125','85126','85129'
,'85140','85141','85142','85143','85144','85145','85146','85149'
,'85160','85161','85162','85163','85164','85165','85166','85169'
,'85180','85181','85182','85183','85184','85185','85186','85189'
,'85200','85201','85202','85203','85204','85205','85206','85209' --Subarachnoid Subdural and

Extradural Hemorrhage Following Injury Without Mention of Open Intracranial Wound
,'85220','85221','85222','85223','85224','85225','85226','85229'
,'85240','85241','85242','85243','85244','85245','85246','85249'
,'85300','85301','85302','85303','85304','85305','85306','85309' --Other and Unspecified Intracranial Hemorrhage Following Injury
,'85400','85401','85402','85403','85404','85405','85406','85409'
,'85110','85111','85112','85113','85114','85115','85116','85119' --Cerebral Laceration and Contusion With Open Intracranial Wound
,'85130','85131','85132','85133','85134','85135','85136','85139'
,'85150','85151','85152','85153','85154','85155','85156','85159'
,'85170','85171','85172','85173','85174','85175','85176','85179'
,'85190','85191','85192','85193','85194','85195','85196','85199'
,'85210','85211','85212','85213','85214','85215','85216','85219' --Subarachnoid Subdural and Extradural Hemorrhage Following Injury With Open Intracranial Wound
,'85230','85231','85232','85233','85234','85235','85236','85239'
,'85250','85251','85252','85253','85254','85255','85256','85259'
,'85310','85311','85312','85313','85314','85315','85316','85319' --Other and Unspecified Intracranial Hemorrhage With Open Intracranial Wound
,'85410','85411','85412','85413','85414','85415','85416','85419' --Intracranial Injury of Other and Unspecified Nature With Open Intracranial Wound
,'80000','80001','80002','80003','80004','80005','80006','80009' --Fracture of Vault of Skull
,'80010','80011','80012','80013','80014','80015','80016','80019'
,'80020','80021','80022','80023','80024','80025','80026','80029'
,'80030','80031','80032','80033','80034','80035','80036','80039'
,'80040','80041','80042','80043','80044','80045','80046','80049'
,'80050','80051','80052','80053','80054','80055','80056','80059'
,'80060','80061','80062','80063','80064','80065','80066','80069'
,'80070','80071','80072','80073','80074','80075','80076','80079'
,'80080','80081','80082','80083','80084','80085','80086','80089'
,'80090','80091','80092','80093','80094','80095','80096','80099'
,'80100','80101','80102','80103','80104','80105','80106','80109' --Fracture of Base of Skull
,'80110','80111','80112','80113','80114','80115','80116','80119'
,'80120','80121','80122','80123','80124','80125','80126','80129'
,'80130','80131','80132','80133','80134','80135','80136','80139'
,'80140','80141','80142','80143','80144','80145','80146','80149'
,'80150','80151','80152','80153','80154','80155','80156','80159'
,'80160','80161','80162','80163','80164','80165','80166','80169'
,'80170','80171','80172','80173','80174','80175','80176','80179'
,'80180','80181','80182','80183','80184','80185','80186','80189'
,'80190','80191','80192','80193','80194','80195','80196','80199'
,'80300','80301','80302','80303','80304','80305','80306','80309' --Other and Unqualified Skull Fractures
,'80310','80311','80312','80313','80314','80315','80316','80319'

,'80320','80321','80322','80323','80324','80325','80326','80329'
,'80330','80331','80332','80333','80334','80335','80336','80339'
,'80340','80341','80342','80343','80344','80345','80346','80349'
,'80350','80351','80352','80353','80354','80355','80356','80359'
,'80360','80361','80362','80363','80364','80365','80366','80369'
,'80370','80371','80372','80373','80374','80375','80376','80379'
,'80380','80381','80382','80383','80384','80385','80386','80389'
,'80390','80391','80392','80393','80394','80395','80396','80399'
,'80400','80401','80402','80403','80404','80405','80406','80409' --Multiple Fractures Involving Skull or Face With Other Bones
,'80410','80411','80412','80413','80414','80415','80416','80419'
,'80420','80421','80422','80423','80424','80425','80426','80429'
,'80430','80431','80432','80433','80434','80435','80436','80439'
,'80440','80441','80442','80443','80444','80445','80446','80449'
,'80450','80451','80452','80453','80454','80455','80456','80459'
,'80460','80461','80462','80463','80464','80465','80466','80469'
,'80470','80471','80472','80473','80474','80475','80476','80479'
,'80480','80481','80482','80483','80484','80485','80486','80489'
,'80490','80491','80492','80493','80494','80495','80496','80499'
,'9501','9502','9503' --Injury to Optic chiasm and Optic Tract
,'9070','95901' --other

ICD Codes for neurosurgical procedures

The Codes for neurosurgical procedures are from NHSN website https://www.cdc.gov/nhsn/xls/icd10-pcs-pcm-nhsn-opc.xlsx

'00160KB','00160ZB','00163KB','00163ZB','00164KB','00164ZB','00500ZZ','00503ZZ'
,'00504ZZ','00510ZZ','00513ZZ','00514ZZ','00520ZZ','00523ZZ','00524ZZ','00560ZZ'
,'00563ZZ','00564ZZ','00570ZZ','00573ZZ','00574ZZ','00580ZZ','00583ZZ','00584ZZ'
,'00590ZZ','00593ZZ','00594ZZ','005A0ZZ','005A3ZZ','005A4ZZ','005B0ZZ','005B3ZZ'
,'005B4ZZ','005C0ZZ','005C3ZZ','005C4ZZ','005D0ZZ','005D3ZZ','005D4ZZ','00760ZZ'
,'00763ZZ','00764ZZ','00800ZZ','00803ZZ','00804ZZ','00870ZZ','00873ZZ','00874ZZ'
,'00880ZZ','00883ZZ','00884ZZ','009000Z','00900ZZ','009030Z','00903ZZ','009040Z'
,'00904ZZ','009100Z','00910ZZ','009130Z','00913ZZ','009140Z','00914ZZ','009200Z'
,'00920ZZ','009230Z','00923ZZ','009240Z','00924ZZ','009300Z','00930ZZ','009330Z'
,'00933ZZ','009340Z','00934ZZ','009400Z','00940ZZ','009430Z','00943ZZ','009440Z'
,'00944ZZ','009500Z','00950ZZ','009530Z','00953ZZ','009540Z','00954ZZ','009600Z'
,'00960ZZ','009630Z','00963ZZ','009640Z','00964ZZ','009700Z','00970ZZ','009730Z'
,'00973ZZ','009740Z','00974ZZ','009800Z','00980ZZ','009830Z','00983ZZ','009840Z'
,'00984ZZ','009900Z','00990ZZ','009930Z','00993ZZ','009940Z','00994ZZ','009A00Z'
,'009A0ZZ','009A30Z','009A3ZZ','009A40Z','009A4ZZ','009B00Z','009B0ZZ','009B30Z'
,'009B3ZZ','009B40Z','009B4ZZ','009C00Z','009C0ZZ','009C30Z','009C3ZZ','009C40Z'
,'009C4ZZ','009D00Z','009D0ZZ','009D30Z','009D3ZZ','009D40Z','009D4ZZ','00B00ZZ'
,'00B03ZZ','00B04ZZ','00B10ZZ','00B13ZZ','00B14ZZ','00B20ZZ','00B23ZZ','00B24ZZ'
,'00B60ZZ','00B63ZZ','00B64ZZ','00B70ZZ','00B73ZZ','00B74ZZ','00B80ZZ','00B83ZZ'
,'00B84ZZ','00B90ZZ','00B93ZZ','00B94ZZ','00BA0ZZ','00BA3ZZ','00BA4ZZ','00BB0ZZ'
,'00BB3ZZ','00BB4ZZ','00BC0ZZ','00BC3ZZ','00BC4ZZ','00BD0ZZ','00BD3ZZ','00C00ZZ'
,'00C03ZZ','00C04ZZ','00C10ZZ','00C13ZZ','00C14ZZ','00C20ZZ','00C23ZZ','00C24ZZ'
,'00C30ZZ','00C33ZZ','00C34ZZ','00C40ZZ','00C43ZZ','00C44ZZ','00C50ZZ','00C53ZZ'
,'00C54ZZ','00C60ZZ','00C63ZZ','00C64ZZ','00C70ZZ','00C73ZZ','00C74ZZ','00C80ZZ'
,'00C83ZZ','00C84ZZ','00C90ZZ','00C93ZZ','00C94ZZ','00CA0ZZ','00CA3ZZ','00CA4ZZ'
,'00CB0ZZ','00CB3ZZ','00CB4ZZ','00CC0ZZ','00CC3ZZ','00CC4ZZ','00CD0ZZ','00CD3ZZ'
,'00CD4ZZ','00D00ZZ','00D03ZZ','00D04ZZ','00D10ZZ','00D13ZZ','00D14ZZ','00D20ZZ'
,'00D23ZZ','00D24ZZ','00D70ZZ','00D73ZZ','00D74ZZ','00F30ZZ','00F33ZZ','00F34ZZ'
,'00F40ZZ','00F43ZZ','00F44ZZ','00F50ZZ','00F53ZZ','00F54ZZ','00F60ZZ','00F63ZZ'
,'00F64ZZ','00H002Z','00H003Z','00H004Z','00H00MZ','00H00YZ','00H032Z','00H033Z'
,'00H03MZ','00H03YZ','00H042Z','00H043Z','00H04MZ','00H04YZ','00H602Z','00H603Z'
,'00H60MZ','00H60YZ','00H632Z','00H633Z','00H63MZ','00H63YZ','00H642Z','00H643Z'
,'00H64MZ','00H64YZ','00J00ZZ','00J03ZZ','00J04ZZ','00K00ZZ','00K03ZZ','00K04ZZ'
,'00K70ZZ','00K73ZZ','00K74ZZ','00K80ZZ','00K83ZZ','00K84ZZ','00K90ZZ','00K93ZZ'
,'00K94ZZ','00KA0ZZ','00KA3ZZ','00KA4ZZ','00KB0ZZ','00KB3ZZ','00KB4ZZ','00KC0ZZ'
,'00KC3ZZ','00KC4ZZ','00KD0ZZ','00KD3ZZ','00KD4ZZ','00N00ZZ','00N03ZZ','00N04ZZ'
,'00N10ZZ','00N13ZZ','00N14ZZ','00N20ZZ','00N23ZZ','00N24ZZ','00N60ZZ','00N63ZZ'
,'00N64ZZ','00N70ZZ','00N73ZZ','00N74ZZ','00N80ZZ','00N83ZZ','00N84ZZ','00N90ZZ'

,'00N93ZZ','00N94ZZ','00NA0ZZ','00NA3ZZ','00NA4ZZ','00NB0ZZ','00NB3ZZ','00NB4ZZ'
,'00NC0ZZ','00NC3ZZ','00NC4ZZ','00ND0ZZ','00ND3ZZ','00ND4ZZ','00P000Z','00P002Z'
,'00P003Z','00P007Z','00P00JZ','00P00KZ','00P00MZ','00P00YZ','00P030Z','00P032Z'
,'00P033Z','00P037Z','00P03JZ','00P03KZ','00P03MZ','00P03YZ','00P040Z','00P042Z'
,'00P043Z','00P047Z','00P04JZ','00P04KZ','00P04MZ','00P04YZ','00P600Z','00P602Z'
,'00P603Z','00P60MZ','00P60YZ','00P630Z','00P632Z','00P633Z','00P63MZ','00P63YZ'
,'00P640Z','00P642Z','00P643Z','00P64MZ','00P64YZ','00Q00ZZ','00Q03ZZ','00Q04ZZ'
,'00Q10ZZ','00Q13ZZ','00Q14ZZ','00Q20ZZ','00Q23ZZ','00Q24ZZ','00Q60ZZ','00Q63ZZ'
,'00Q64ZZ','00Q70ZZ','00Q73ZZ','00Q74ZZ','00Q80ZZ','00Q83ZZ','00Q84ZZ','00Q90ZZ'
,'00Q93ZZ','00Q94ZZ','00QA0ZZ','00QA3ZZ','00QA4ZZ','00QB0ZZ','00QB3ZZ','00QB4ZZ'
,'00QC0ZZ','00QC3ZZ','00QC4ZZ','00QD0ZZ','00QD3ZZ','00QD4ZZ','00R107Z','00R10JZ'
,'00R10KZ','00R147Z','00R14JZ','00R14KZ','00R207Z','00R20JZ','00R20KZ','00R247Z'
,'00R24JZ','00R24KZ','00R607Z','00R60JZ','00R60KZ','00R647Z','00R64JZ','00R64KZ'
,'00T70ZZ','00T73ZZ','00T74ZZ','00U107Z','00U10JZ','00U10KZ','00U137Z','00U13JZ'
,'00U13KZ','00U147Z','00U14JZ','00U14KZ','00U207Z','00U20JZ','00U20KZ','00U237Z'
,'00U23JZ','00U23KZ','00U247Z','00U24JZ','00U24KZ','00U607Z','00U60JZ','00U60KZ'
,'00U637Z','00U63JZ','00U63KZ','00U647Z','00U64JZ','00U64KZ','00W000Z','00W002Z'
,'00W003Z','00W007Z','00W00JZ','00W00KZ','00W00MZ','00W00YZ','00W030Z','00W032Z'
,'00W033Z','00W037Z','00W03JZ','00W03KZ','00W03MZ','00W03YZ','00W040Z','00W042Z'
,'00W043Z','00W047Z','00W04JZ','00W04KZ','00W04MZ','00W04YZ','00W600Z','00W602Z'
,'00W603Z','00W60MZ','00W60YZ','00W630Z','00W632Z','00W633Z','00W63MZ','00W63YZ'
,'00W640Z','00W642Z','00W643Z','00W64MZ','00W64YZ','031G09G','031G0AG','031G0JG'
,'031G0KG','031G0ZG','031S09G','031S0AG','031S0JG','031S0KG','031S0ZG','031T09G'
,'031T0AG','031T0JG','031T0KG','031T0ZG','035G0ZZ','035G3ZZ','035G4ZZ','037G04Z'
,'037G05Z','037G06Z','037G07Z','037G0DZ','037G0EZ','037G0FZ','037G0GZ','037G0ZZ'
,'037G34Z','037G35Z','037G36Z','037G37Z','037G3DZ','037G3EZ','037G3FZ','037G3GZ'
,'037G3ZZ','037G44Z','037G45Z','037G46Z','037G47Z','037G4DZ','037G4EZ','037G4FZ'
,'037G4GZ','037G4ZZ','039G00Z','039G0ZZ','039G30Z','039G3ZZ','039G40Z','039G4ZZ'
,'03BG0ZZ','03BG3ZZ','03BG4ZZ','03CG0ZZ','03CG3Z7','03CG3ZZ','03CG4ZZ','03LG0BZ'
,'03LG0CZ','03LG0DZ','03LG0ZZ','03LG3CZ','03LG3ZZ','03LG4BZ','03LG4CZ','03LG4DZ'
,'03LG4ZZ','03RG07Z','03RG0JZ','03RG0KZ','03RG47Z','03RG4JZ','03RG4KZ','03UG07Z'
,'03UG0JZ','03UG0KZ','03UG37Z','03UG3JZ','03UG3KZ','03UG47Z','03UG4JZ','03UG4KZ'
,'03VG0BZ','03VG0CZ','03VG0DZ','03VG0HZ','03VG0ZZ','03VG3BZ','03VG3CZ','03VG3DZ'
,'03VG3HZ','03VG3ZZ','03VG4BZ','03VG4CZ','03VG4DZ','03VG4HZ','03VG4ZZ','055L0ZZ'
,'055L3ZZ','055L4ZZ','05BL0ZZ','05BL3ZZ','05BL4ZZ','05LL0CZ','05LL0DZ','05LL0ZZ'
,'05LL3CZ','05LL3ZZ','05LL4CZ','05LL4DZ','05LL4ZZ','05RL07Z','05RL0JZ','05RL0KZ'
,'05RL47Z','05RL4JZ','05RL4KZ','0G500ZZ','0G503ZZ','0G504ZZ','0G510ZZ','0G513ZZ'
,'0G514ZZ','0G800ZZ','0G803ZZ','0G804ZZ','0G9000Z','0G900ZZ','0G9030Z','0G903ZZ'
,'0G9040Z','0G904ZZ','0G9100Z','0G910ZZ','0G9130Z','0G913ZZ','0G9140Z','0G914ZZ'
,'0GB00ZZ','0GB03ZZ','0GB04ZZ','0GB10ZZ','0GB13ZZ','0GB14ZZ','0GC00ZZ','0GC03ZZ'
,'0GC04ZZ','0GC10ZZ','0GC13ZZ','0GC14ZZ','0GJ00ZZ','0GJ03ZZ','0GJ04ZZ','0GJ10ZZ'
,'0GJ13ZZ','0GJ14ZZ','0GN00ZZ','0GN03ZZ','0GN04ZZ','0GN10ZZ','0GN13ZZ','0GN14ZZ'
,'0GP000Z','0GP030Z','0GP040Z','0GP100Z','0GP130Z','0GP140Z','0GQ00ZZ','0GQ03ZZ'

,'0GQ04ZZ','0GQ10ZZ','0GQ13ZZ','0GQ14ZZ','0GT00ZZ','0GT04ZZ','0GT10ZZ','0GT14ZZ'
,'0GW000Z','0GW030Z','0GW040Z','0GW100Z','0GW130Z','0GW140Z','0W110J9','0W110JB'
,'0W110JG','0W110JJ','0W9100Z','0W910ZZ','0W9130Z','0W913ZZ','0W9140Z','0W914ZZ'
,'0WC10ZZ','0WC13ZZ','0WC14ZZ','0WF10ZZ','0WF13ZZ','0WF14ZZ','0WH101Z','0WH103Z'
,'0WH10YZ','0WH131Z','0WH133Z','0WH13YZ','0WH141Z','0WH143Z','0WH14YZ','0WJ10ZZ'
,'0WJ13ZZ','0WJ14ZZ','0WP100Z','0WP101Z','0WP103Z','0WP10JZ','0WP10YZ','0WP130Z'
,'0WP131Z','0WP133Z','0WP13JZ','0WP13YZ','0WP140Z','0WP141Z','0WP143Z','0WP14JZ'
,'0WP14YZ','0WW100Z','0WW101Z','0WW103Z','0WW10JZ','0WW10YZ','0WW130Z','0WW131Z'
,'0WW133Z','0WW13JZ','0WW13YZ','0WW140Z','0WW141Z','0WW143Z','0WW14JZ','0WW14YZ'
,'3E0Q004','3E0Q005','3E0Q00M','3E0Q028','3E0Q029','3E0Q03Z','3E0Q06Z','3E0Q07Z'
,'3E0Q0AZ','3E0Q0BZ','3E0Q0E0','3E0Q0E1','3E0Q0GC','3E0Q0HZ','3E0Q0KZ','3E0Q0NZ'
,'3E0Q0SF','3E0Q0TZ','0101','0102','0109','0110','0111','0112','0113','0114'
,'0115','0116','0117','0118','0119','0120','0121','0122'
,'0123','0124','0125','0126','0127','0128','0129','0131'
,'0132','0139','0141','0142','0151','0152','0153','0159'
,'016' ,'0201','0202','0203','0204','0205','0206','0207'
,'0211','0212','0213','0214','022' ,'0221','0222','0231'
,'0232','0233','0234','0235','0239','0241','0242','0243'
,'0291','0292','0293','0294','0295','0296','0299'

Missing value of data from the MIMIC-III database table S1

skim_variable	n_missing	complete_rate
hadm_id	0	100%
icustay_id	0	100%
gender	0	100%
age	0	100%
bun_max	20	99%
alb	1055	55%
hr_max	17	99%
mbp_min	17	99%
rr_max	19	99%
t_max	18	99%
spo2_min	17	99%
glu_max	31	99%
sb_min	34	99%
hct_min	19	99%
hb_min	21	99%
plt_min	20	99%
wbc_max	20	99%
ag_max	36	99%
cr_max	20	99%
ca_min	208	91%
cl_max	18	99%
na_max	19	99%
k_min	17	99%
inr_max	95	96%
pt_max	95	96%
ptt_max	102	96%
phosphate	196	92%
mg	123	95%
rdw	27	99%
sofa	0	100%
apsiii	0	100%
congestive_heart_failure	0	100%
chronic_pulmonary_disease	0	100%
rheumatic_disease	0	100%
renal_disease	0	100%
diabetes	0	100%
liver_disease	0	100%

charlson	0	100%
gcs_min	17	99%
intraparenchymal_hemorrhage	0	100%
extradural_hemorrhage	0	100%
subdural_hemorrhage	0	100%
subarachnoi_hemorrhage	0	100%
firstday_rbc	0	100%
firstday_plt	0	100%
neurosurgery	0	100%
sepsis	0	100%
flag	0	100%
los_icu	0	100%
los_hospital	0	100%
onemouthsurvival	0	100%
onemonthmortality	0	100%
threemonthsurvival	0	100%
threemonthmortality	0	100%
oneyearsurvival	0	100%
oneyearmortality	0	100%
ph_min	1046	56%
po2_min	1046	56%
pco2_max	1046	56%
aado2_calc_max	1288	45%
pao2fio2_min	1288	45%
lactate_max	1391	41%
bilirubin_max	1747	26%
neutrophils_abs_max	1213	48%
lymphocytes_abs_min	1213	48%
sii	1217	48%
ferritin_max	2317	1%

Table S1 missing value of data from the MIMIC-III database

Missing value of data from the MIMIC-IV database table S2

skim_variable	n_missing	complete_rate
hadm_id	0	100%
icustay_id	0	100%
gender	0	100%
age	0	100%
los_icu	0	100%
los_hospital	0	100%
height	1883	37%
weight	1883	37%
bmi	1883	37%
hr_max	4	100%
mbp_min	6	100%
rr_max	12	100%
t_max	15	100%
spo2_min	4	100%
glu_max	66	98%
ph_min	1705	43%
po2_min	1705	43%
pco2_max	1705	43%
aado2_max	1997	33%
pf_ratio_min	1997	33%
sb_min	46	99%
lac_max	2093	30%
hct_min	20	99%
hb_min	21	99%
plt_min	21	99%
wbc_max	21	99%
alb	1687	44%
ag_max	46	99%
bun_max	23	99%
cr_max	20	99%
ca_min	135	96%
cl_max	44	99%
na_max	44	99%
k_min	43	99%
lymabs_min	1368	54%
neutro_max	1368	54%
sii	1371	54%
inr_max	147	95%
pt_max	147	95%

ptt_max	153	95%
alt_max	1903	37%
ast_max	1901	37%
alp_max	1957	35%
bili_max	1978	34%
ldh_max	2503	16%
phosphate	54	98%
mg	56	98%
rdw	168	94%
sofa	0	100%
apsiii	0	100%
congestive_heart_failure	0	100%
chronic_pulmonary_disease	0	100%
rheumatic_disease	0	100%
renal_disease	0	100%
diabetes	0	100%
liver_disease	0	100%
charlson	0	100%
gcs_min	5	100%
ferritin	2757	8%
intraparenchymal_heorrhage	0	100%
extradural_hemorrhage	0	100%
subdural_hemorrhage	0	100%
subarachnoi_hemorrhage	0	100%
firstday_rbc	0	100%
firstday_plt	0	100%
neurosurgery	0	100%
sepsis	0	100%
flag	0	100%

Table S2 missing value of data from the MIMIC-IV database

Cox regression analysis of three month table S3

Characteristic	Univariate model		Multivariate model	
	HR 95%CI	p	HR 95%CI	p
Age (years)	1.00 (1.00-1.01)	<0.001		
Male sex (%)	0.81 (0.67-0.98)	0.032	0.82 (0.67-1.01)	0.068
**Viatl signs**				
Average arterial pressure(mmHg)	0.98 (0.97-0.98)	<0.001	Not selected	
Heart rate (beats/minute)	1.01 (1.00-1.01)	<0.001	Not selected	
Respiratory rate (beats/minute)	1.04 (1.02-1.05)	<0.001	Not selected	
Blood oxygen saturation (%)	0.99 (0.98-1.01)	0.365	-	
Temperature (℃)	1.36 (1.20-1.54)	<0.001	**1.34 (1.17-1.53)**	**<0.001**
**Blood differential**				
white blood cell (10^9/L)	1.00 (1.00-1.00)	<0.001	Not selected	
Hemoglobin (10^{12}/L)	0.81 (0.78-0.84)	<0.001	**0.92 (0.87-0.97)**	**0.003**
Platelet (10^9/L)	1.00 (1.00-1.00)	<0.001	Not selected	
RDW (%)	1.18 (1.13-1.23)	<0.001	1.04 (0.99-1.10)	0.116
**Electrolyte**				
Sodium (mmol/L)	1.08 (1.06-1.11)	<0.001	**1.10 (1.06-1.14)**	**<0.001**
Potassium (mmol/L)	0.96 (0.80-1.15)	0.656	-	
Chloride (mmol/L)	1.05 (1.04-1.07)	<0.001	**0.95 (0.92-0.98)**	**0.001**
Calcium (mmol/L)	0.94 (0.85-1.04)	0.217	-	
Phosphate (mmol/L)	1.20 (1.10-1.30)	<0.001	0.95 (0.87-1.05)	0.320
Magnesium (mmol/L)	1.60 (1.23-2.09)	<0.001	**1.54 (1.17-2.01)**	**0.002**
Anion gap (mmol/L)	1.07 (1.05-1.09)	<0.001	0.98 (0.95-1.01)	0.257
**Serum biochemical**				
Creatinine (mg/dL)	1.17 (1.11-1.24)	<0.001	**0.86 (0.77-0.97)**	**0.013**
Bicarbonate (mmol/L)	0.91 (0.89-0.93)	<0.001	**0.92 (0.88-0.95)**	**<0.001**
Glucose (mmol/L)	1.00 (1.00-1.00)	<0.001	Not selected	
**Coagulation**			-	
PT (s)	1.01 (1.01-1.02)	<0.001	Not selected	
APTT (s)	1.01 (1.01-1.02)	<0.001	Not selected	
**Score**				
GCS score	0.89 (0.87-0.91)	<0.001	**0.94 (0.91-0.96)**	**<0.001**
APS-III score	1.03 (1.03-1.03)	<0.001	Not selected	
SOFA score	1.19 (1.16-1.22)	<0.001	1.04 (1.00-1.08)	0.080
**Comorbidity**				
Charlson	1.16 (1.13-1.20)	<0.001	**1.09 (1.03-1.14)**	**0.001**
Congestive heart failure	2.40 (1.95-2.97)	<0.001	1.29 (0.99-1.69)	0.060
Chronic pulmonary disease	1.07 (0.81-1.42)	0.622	-	
Rheumatic disease	1.27 (0.68-2.38)	0.452	-	
Renal disease	2.48 (1.95-3.15)	<0.001	0.92 (0.64-1.33)	0.665
Diabetes	1.69 (1.37-2.09)	<0.001	0.99 (0.77-1.28)	0.962
Liver disease	1.25 (0.92-1.71)	0.159		
**Type of trauma**				
Intraparenchymal heorrhage	1.48 (1.10-1.98)	0.009	**1.47 (1.09-1.98)**	**0.011**
Extradural hemorrhage	1.27 (0.68-2.38)	0.455	-	
Subdural hemorrhage	1.11 (0.92-1.35)	0.266	-	
Subarachnoid hemorrhage	1.33 (1.08-1.64)	0.008	1.23 (0.99-1.54)	0.065
Neurosurgery	1.38 (1.13-1.67)	0.001	**1.41 (1.14-1.75)**	**0.002**
**Blood products**				
Firstday RBC infusion	1.92 (1.53-2.40)	<0.001	0.99 (0.74-1.32)	0.955
Firstday PLT infusion	1.71 (1.33-2.22)	<0.001	0.94 (0.70-1.26)	0.668
**BAR**				
Group1	Reference	-	-	
Group2	2.21 (1.77-2.76)	0.000	**1.77 (1.39-2.25)**	**<0.001**
Group3	5.18 (4.05-6.64)	0.000	**2.94 (2.06-4.18)**	**<0.001**

RDW, red blood cell distribution width; BAR, blood urea nitrogen-to-albumin ratio; INR, International normalized ratio; PT, prothrombin time; APTT, activated partial thromboplastin time; GCS, glasgow coma scale; APS-III, acute physiology score III; SOFA, sequential organ failure assessment; RBC, red blood cell; PLT, platelet;

Table S3: Cox regression analysis of three month

Cox risk-proportional model results represented as the HR (95%CI).

Cox regression analysis of one year table S4

Characteristic	Univariate model		Multivariate model	
	HR 95%CI	p	HR 95%CI	p
Age (years)	1.00 (1.00-1.01)	<0.001	Not selected	
Male sex (%)	0.77 (0.65-0.92)	0.004	0.80 (0.66-0.97)	0.024
**Viatl signs**				
Average arterial pressure(mmHg)	0.98 (0.98-0.99)	<0.001	Not selected	
Heart rate (beats/minute)	1.01 (1.00-1.01)	0.002	Not selected	
Respiratory rate (beats/minute)	1.04 (1.02-1.05)	<0.001	Not selected	
Blood oxygen saturation (%)	0.99 (0.98-1.01)	0.256	-	
Temperature (℃)	1.24 (1.10-1.39)	<0.001	1.24 (1.09-1.41)	0.001
**Blood differential**				
white blood cell (10^9/L)	1.00 (1.00-1.00)	0.001	Not selected	
Hemoglobin (10^{12}/L)	0.82 (0.79-0.85)	<0.001	0.92 (0.87-0.97)	0.002
Platelet (10^9/L)	1.00 (1.00-1.00)	<0.001	Not selected	
RDW (%)	1.19 (1.15-1.24)	<0.001	1.06 (1.01-1.11)	0.020
**Electrolyte**				
Sodium (mmol/L)	1.07 (1.05-1.09)	<0.001	1.10 (1.07-1.14)	<0.001
Potassium (mmol/L)	1.05 (0.89-1.25)	0.554	-	
Chloride (mmol/L)	1.04 (1.02-1.05)	<0.001	0.94 (0.91-0.97)	<0.001
Calcium (mmol/L)	0.96 (0.87-1.06)	0.405	-	
Phosphate (mmol/L)	1.17 (1.08-1.26)	<0.001	0.94 (0.86-1.03)	0.194
Magnesium (mmol/L)	1.42 (1.11-1.82)	0.006	1.40 (1.09-1.80)	0.009
Anion gap (mmol/L)	1.07 (1.05-1.09)	<0.001	0.98 (0.96-1.01)	0.294
**Serum biochemical**				
Creatinine (mg/dL)	1.18 (1.12-1.24)	<0.001	0.87 (0.79-0.97)	0.011
Bicarbonate (mmol/L)	0.93 (0.91-0.95)	<0.001	0.93 (0.89-0.96)	<0.001
Glucose (mmol/L)	1.00 (1.00-1.00)	<0.001	Not selected	
**Coagulation**				
PT (s)	1.01 (1.01-1.02)	<0.001	Not selected	
APTT (s)	1.01 (1.01-1.02)	<0.001	Not selected	
**Score**				
GCS score	0.89 (0.87-0.91)	<0.001	0.93 (0.91-0.96)	<0.001
APS-III score	1.03 (1.03-1.03)	<0.001	Not selected	
SOFA score	1.18 (1.15-1.21)	<0.001	1.04 (1.00-1.09)	0.032
**Comorbidity**				
Charlson	1.18 (1.15-1.21)	<0.001	1.11 (1.07-1.17)	<0.001
Congestive heart failure	2.45 (2.02-2.98)	<0.001	1.21 (0.95-1.55)	0.125
Chronic pulmonary disease	1.17 (0.91-1.51)	0.217	-	
Rheumatic disease	1.20 (0.66-2.18)	0.553	-	
Renal disease	2.43 (1.94-3.04)	<0.001	0.79 (0.56-1.11)	0.169
Diabetes	1.81 (1.50-2.19)	<0.001	1.05 (0.84-1.32)	0.662
Liver disease	1.27 (0.96-1.69)	0.100	-	
**Type of trauma**				
Intraparenchymal heorrhage	1.41 (1.07-1.86)	0.014	1.34 (1.01-1.79)	0.042
Extradural hemorrhage	1.19 (0.66-2.17)	0.561	-	
Subdural hemorrhage	1.21 (1.02-1.44)	0.031	0.88 (0.72-1.07)	0.184
Subarachnoid hemorrhage	1.34 (1.10-1.63)	0.003	1.20 (0.98-1.47)	0.081
Neurosurgery	1.31 (1.09-1.57)	0.004	1.43 (1.17-1.76)	0.001
**Blood products**				
Firstday RBC infusion	1.78 (1.44-2.20)	<0.001	0.96 (0.73-1.26)	0.780
Firstday PLT infusion	1.69 (1.33-2.14)	<0.001	0.92 (0.70-1.21)	0.541
**BAR**				
Group1	Reference	-	Reference	-
Group2	2.18 (1.78-2.67)	<0.001	1.72 (1.38-2.14)	<0.001
Group3	4.98 (3.95-6.26)	<0.001	2.83 (2.04-3.93)	<0.001

RDW, red blood cell distribution width; BAR, blood urea nitrogen-to-albumin ratio; INR, International normalized ratio; PT, prothrombin time; APTT, activated partial thromboplastin time; GCS, glasgow coma scale; APS-III, acute physiology score III; SOFA, sequential organ failure assessment; RBC, red blood cell; PLT, platelet;

Table S4: Cox regression analysis of one year

Cox risk-proportional model results represented as the HR (95%CI).

Logistic regression analysis table S5

Characteristic	Univariate model		Multivariate model	
	OR 95%CI	p	OR 95%CI	p
Age (years)	1.01 (1.00-1.01)	< 0.001	Not selected	
Male sex (%)	0.77 (0.61-0.98)	0.03	0.76 (0.58-1.00)	0.054
**Viatl signs**				
Average arterial pressure(mmHg)	0.97 (0.96-0.98)	< 0.001	Not selected	
Heart rate (beats/minute)	1.01 (1.01-1.02)	< 0.001	Not selected	
Respiratory rate (beats/minute)	1.04 (1.02-1.06)	< 0.001	Not selected	
Blood oxygen saturation (%)	0.99 (0.98-1.01)	0.37	-	
Temperature (℃)	1.62 (1.39-1.88)	< 0.001	1.65 (1.38-1.97)	< 0.001
**Blood differential**				
white blood cell (10^9/L)	1.05 (1.03-1.06)	< 0.001	1.02 (1.00-1.04)	0.020
Hemoglobin (10^{12}/L)	0.78 (0.74-0.83)	< 0.001	0.92 (0.85-0.99)	0.035
Platelet (10^9/L)	1.00 (1.00-1.00)	< 0.001	Not selected	
RDW (%)	1.23 (1.16-1.3)	< 0.001	1.12 (1.04-1.21)	0.002
**Electrolyte**				
Sodium (mmol/L)	1.09 (1.07-1.12)	< 0.001	1.09 (1.03-1.14)	< 0.001
Potassium (mmol/L)	0.75 (0.60-0.94)	0.01	0.83 (0.64-1.08)	0.168
Chloride (mmol/L)	1.07 (1.05-1.09)	< 0.001	0.97 (0.92-1.01)	0.153
Calcium (mmol/L)	0.93 (0.83-1.05)	0.26	-	
Phosphate (mmol/L)	1.23 (1.11-1.37)	< 0.001	0.99 (0.87-1.13)	0.869
Magnesium (mmol/L)	1.73 (1.24-2.4)	< 0.001	2.01 (1.38-2.91)	< 0.001
Anion gap (mmol/L)	1.09 (1.06-1.12)	< 0.001	1.01 (0.96-1.05)	0.835
**Serum biochemical**				
Creatinine (mg/dL)	1.16 (1.06-1.27)	< 0.001	0.76 (0.63-0.92)	0.005
Bicarbonate (mmol/L)	0.89 (0.87-0.92)	< 0.001	0.92 (0.87-0.98)	0.005
Glucose (mmol/L)	1.00 (1.00-1.00)	< 0.001	Not selected	
**Coagulation**				
PT (s)	1.02 (1.01-1.03)	< 0.001	Not selected	
APTT (s)	1.02 (1.01-1.02)	< 0.001	Not selected	
**Score**				
GCS score	0.90 (0.87-0.93)	< 0.001	0.96 (0.92-1.00)	0.035
APS-III score	1.04 (1.03-1.04)	< 0.001	Not selected	
SOFA score	1.22 (1.18-1.27)	< 0.001	1.06 (1.00-1.12)	0.042
**Comorbidity**				
Charlson	1.14 (1.1-1.19)	< 0.001	1.11 (1.03-1.19)	0.004
Congestive heart failure	2.45 (1.86-3.23)	< 0.001	1.48 (1.02-2.15)	0.041
Chronic pulmonary disease	0.91 (0.63-1.31)	0.61	-	
Rheumatic disease	1.36 (0.63-2.94)	0.43	-	
Renal disease	1.9 (1.35-2.67)	< 0.001	0.7 (0.42-1.19)	0.186
Diabetes	1.65 (1.27-2.15)	< 0.001	1.12 (0.79-1.58)	0.532
Liver disease	1.4 (0.96-2.05)	0.08	-	
**Type of trauma**				
Intraparenchymal heorrhage	1.72 (1.21-2.46)	< 0.001	1.89 (1.27-2.83)	0.002
Extradural hemorrhage	1.33 (0.62-2.86)	0.47	-	
Subdural hemorrhage	1.03 (0.82-1.31)	0.78	-	
Subarachnoid hemorrhage	1.41 (1.09-1.83)	0.01	1.41 (1.05-1.9)	0.023
Neurosurgery	1.63 (1.29-2.07)	< 0.001	1.79 (1.35-2.38)	< 0.001
**Blood products**				
Firstday RBC infusion	2.19 (1.66-2.89)	< 0.001	0.90 (0.60-1.35)	0.616
Firstday PLT infusion	2.07 (1.51-2.83)	< 0.001	1.05 (0.70-1.56)	0.826
**BAR**				
Group1	Reference	-	-	-
Group2	2.17 (1.66-2.84)	< 0.001	1.75 (1.28-2.40)	< 0.001
Group3	5.62 (4.09-7.72)	< 0.001	3.50 (2.14-5.72)	< 0.001

RDW, red blood cell distribution width; BAR, blood urea nitrogen-to-albumin ratio; INR, International normalized ratio; PT, prothrombin time; APTT, activated partial thromboplastin time; GCS, glasgow coma scale; APS-III, acute physiology score III; SOFA, sequential organ failure assessment; RBC, red blood cell; PLT, platelet;

Table S5: Logistic regression analysis

Logistic regression model results represented as the OR (95%CI).

AUC of ROC table S6

	AUC (95% CI)			
	BAR	GCS	SOFA	APS-III
**1-month**	0.67 (0.64-0.70)	0.55 (0.51-0.59)	0.68 (0.65-0.71)	0.73 (0.71-0.76)
**3-month**	0.68 (0.65-0.71)	0.57 (0.53-0.60)	0.69 (0.67-0.72)	0.74 (0.71-0.76)
**1-year**	0.68 (0.65-0.70)	0.58 (0.55-0.61)	0.69 (0.66-0.71)	0.73 (0.70-0.75)

Table S6. AUC of BAR, GCS, SOFA, APS-III with mortality of one month, three month, and one year

Machine learning Model Efficacy tables S7~S14

model	acc	auc	f	recall	precision
adaboost classifier	0.87739464	0.817122	0.441331	0.368421	0.550218
	0.8697318				
	0.86153846				
	0.87307692				
	0.87307692				
	0.9				
	0.88461538				
	0.88076923				
	0.88846154				
	0.86538462				
avg	0.877404951				
sd	0.011492355				

Table S7. Modeling Efficacy:Adaboost classifier

model	acc	auc	f	recall	precision
decision tree classifier	0.83908046	0.656131	0.405365	0.397661	0.413374
	0.8467433				
	0.81153846				
	0.84615385				
	0.85				
	0.84230769				
	0.86538462				
	0.83846154				
	0.86153846				
	0.86538462				
avg	0.8466593				
sd	0.016024771				

Table S8. Modeling Efficacy: decision tree classifier

model	acc	auc	f	recall	precision
GaussianNB	0.81609195	0.754521	0.372208	0.438596	0.323276
	0.81226054				
	0.8				
	0.78461538				
	0.81538462				
	0.80769231				
	0.78076923				
	0.78076923				
	0.82692308				

	0.83076923
avg	0.805527557

Table S9. Modeling Efficacy: Navie Bayes classifier

model	acc	auc	f	recall	precision
gradientboost	0.89272031	0.872394	0.535902	0.447368	0.668122
	0.87356322				
	0.86923077				
	0.89230769				
	0.90769231				
	0.88846154				
	0.93076923				
	0.90769231				
	0.93076923				
	0.88846154				
avg	0.898166815				
sd	0.021087951				

Table S10. Modeling Efficacy: gradientboost classifier

model	acc	auc	f	recall	precision
light gradient boost	0.90804598	0.888017	0.559715	0.459064	0.716895
	0.88888889				
	0.89230769				
	0.90384615				
	0.88461538				
	0.91538462				
	0.92692308				
	0.91153846				
	0.93076923				
	0.88846154				
avg	0.905078102				
sd	0.016382683				

Table S11. Modeling Efficacy: light gradient boost classifier

model	acc	auc	f	recall	precision
logistic reregression	0.86590038	0.755992	0.206235	0.125731	0.573333
	0.87739464				
	0.87307692				
	0.88461538				
	0.87307692				
	0.86538462				
	0.87307692				
	0.85769231				

	0.87692308
	0.88076923
avg	0.87279104
sd	0.00797417

Table S12. Modeling Efficacy: logistic reregression classifier

model	acc	auc	f	recall	precision
random forest classifier	0.88122605	0.891884	0.361111	0.22807	0.866667
	0.88888889				
	0.89230769				
	0.89615385				
	0.89230769				
	0.9				
	0.90769231				
	0.89230769				
	0.90384615				
	0.88461538				
avg	0.89393457				
sd	0.008239352				

Table S13. Modeling Efficacy: random forest classifier

model	acc	auc	f	recall	precision
XGboost	0.90038314	0.895087	0.532348	0.421053	0.723618
	0.89655172				
	0.88076923				
	0.90769231				
	0.88846154				
	0.9				
	0.91538462				
	0.91923077				
	0.93076923				
	0.88846154				
avg	0.90277041				
sd	0.015577072				

Table S14. Modeling Efficacy: XGboost classifier

Machine Learning feature importance Figures S1~S8

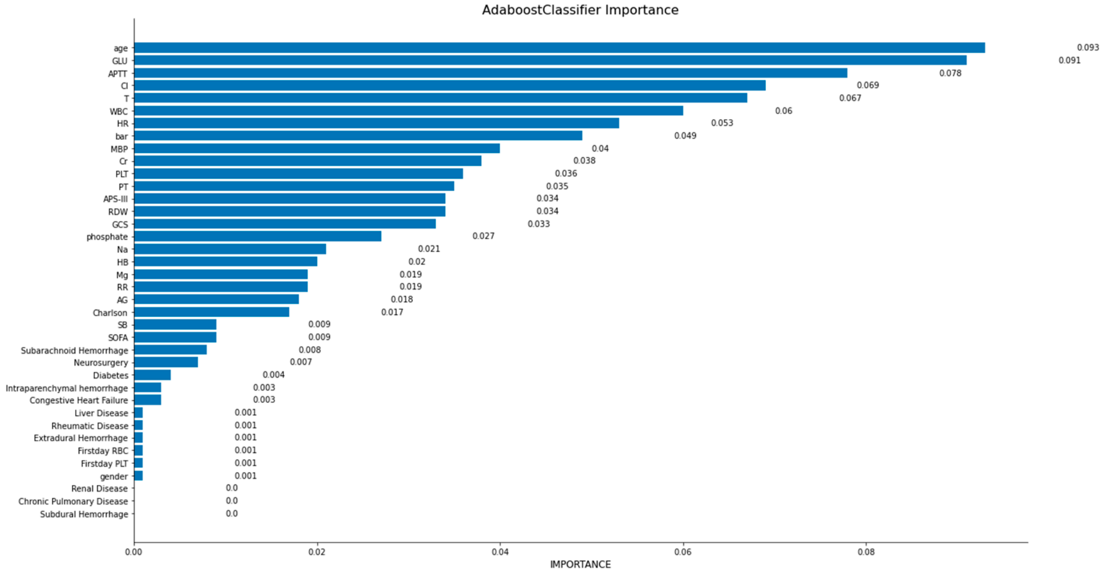

Figure S1: Importance ranking of adaboost classifier

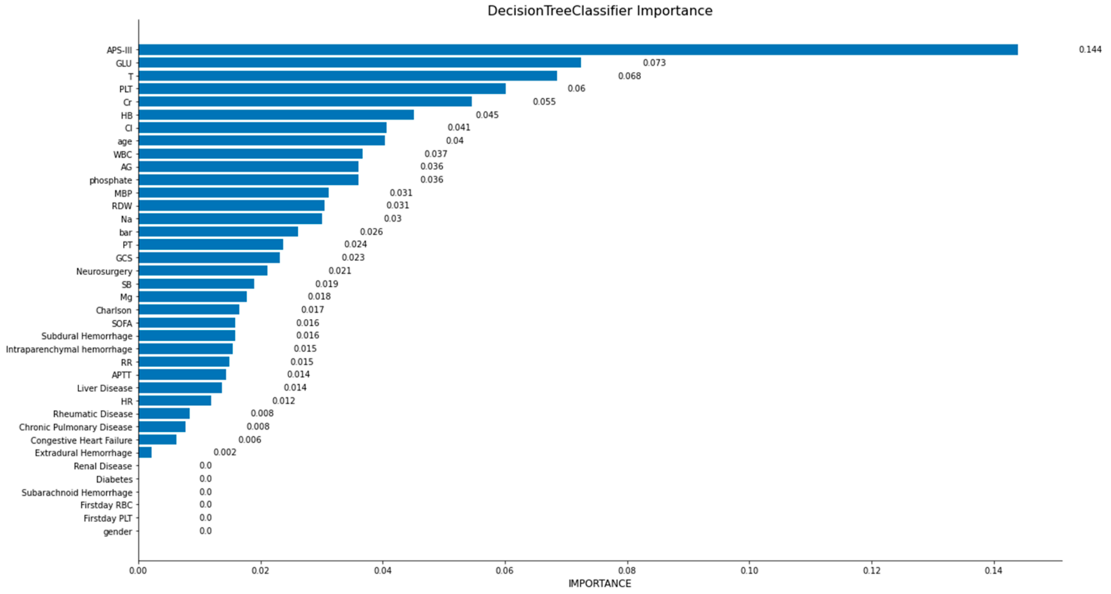

Figure S2: Importance ranking of Decision tree classifier

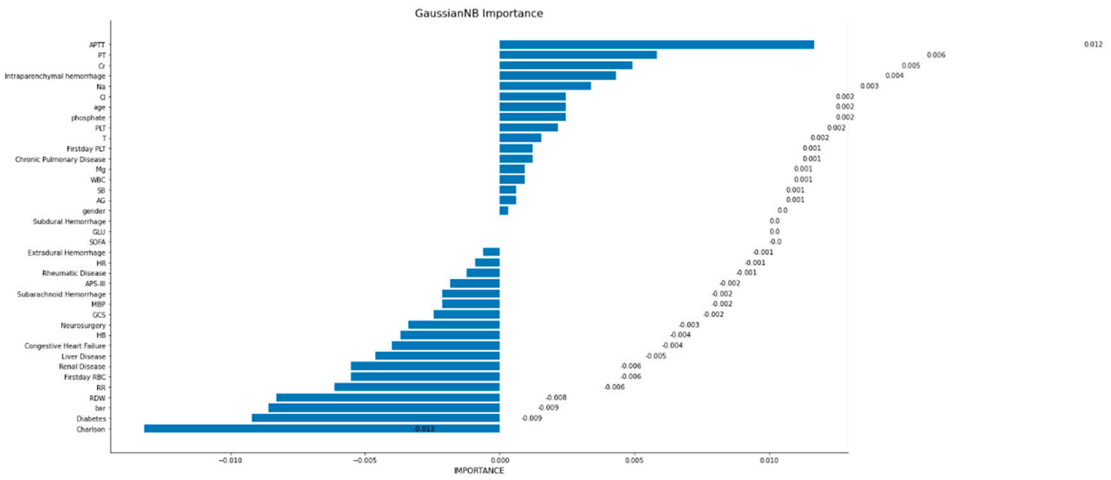

Figure S3: Importance ranking of Navie Bayes classifier

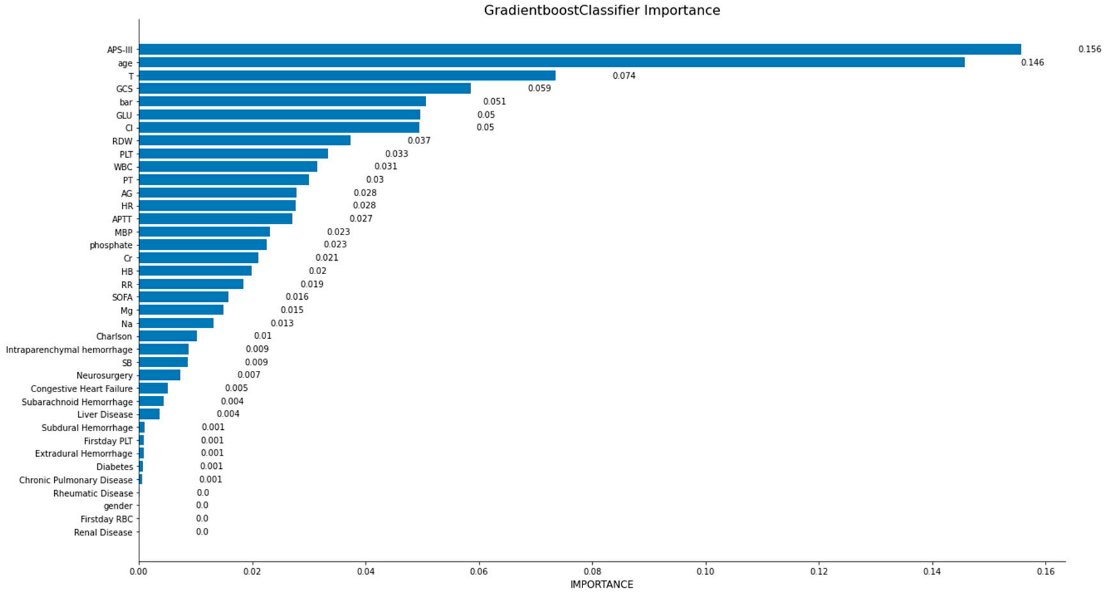

Figure S4: Importance ranking of Gradient Boost classifier

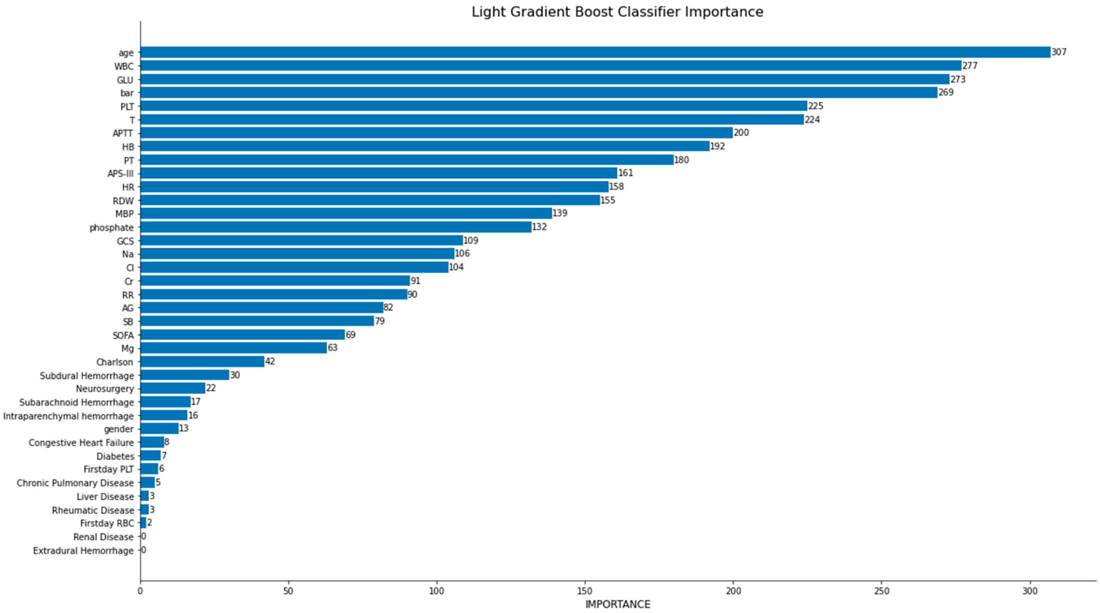

Figure S5: Importance ranking of Light Gradient boost classifier

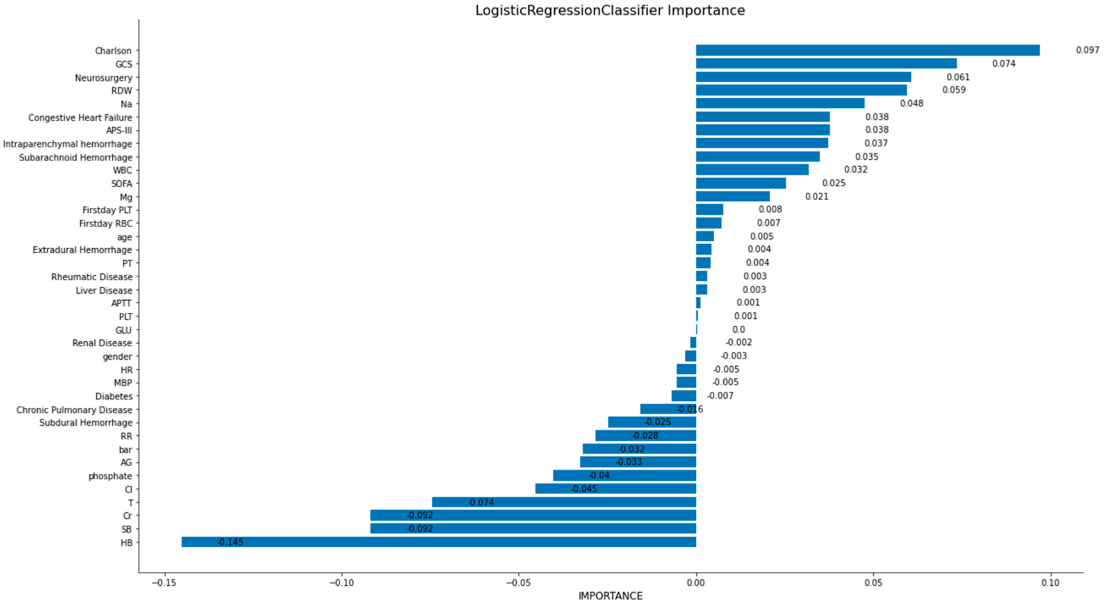

Figure S6: Importance ranking of Logistic regression classifier

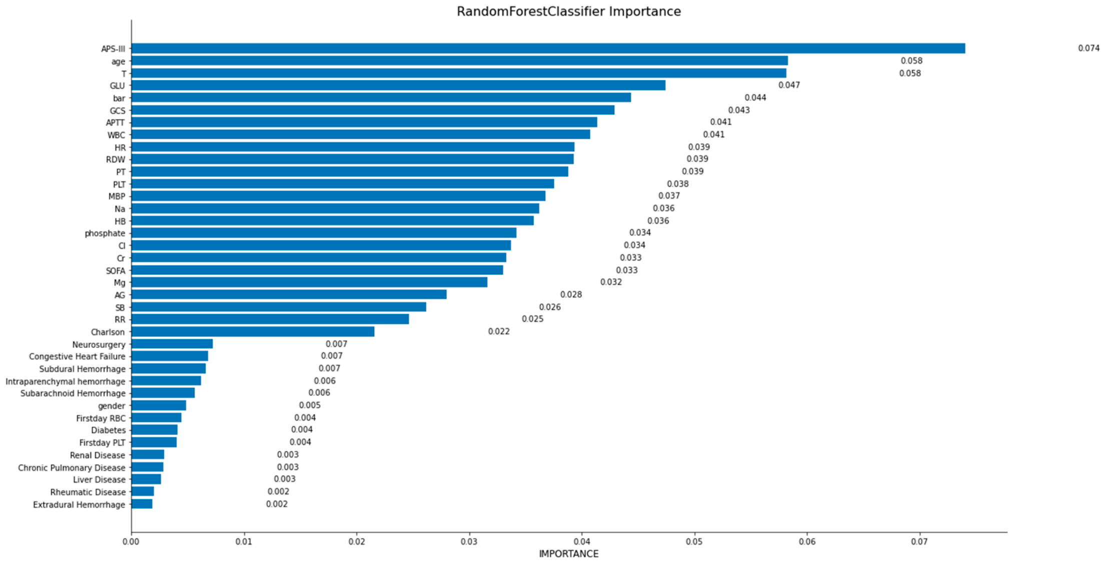

Figure S7: Importance ranking of random forest classifier

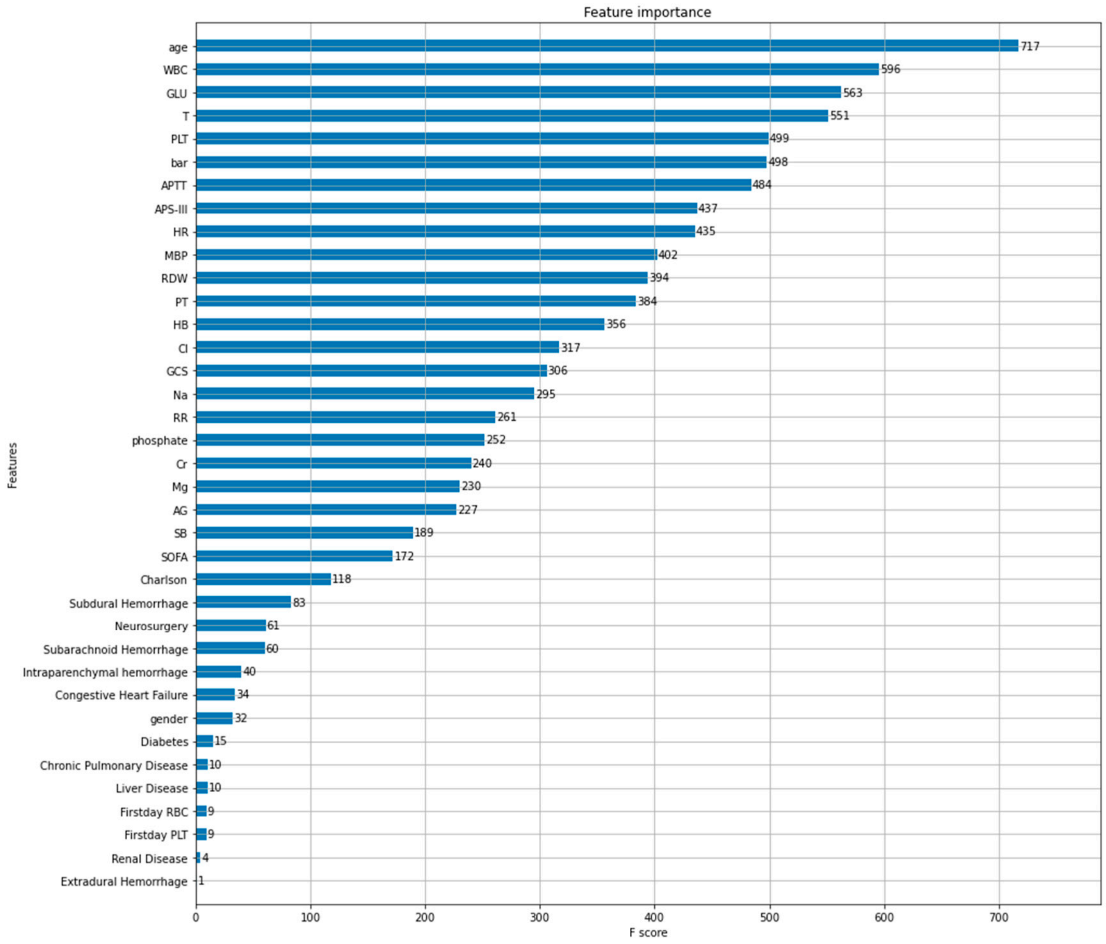

Figure S8: Importance ranking of Xgboost classifier

ROC curves of machine learning classifier Figures S9~S15

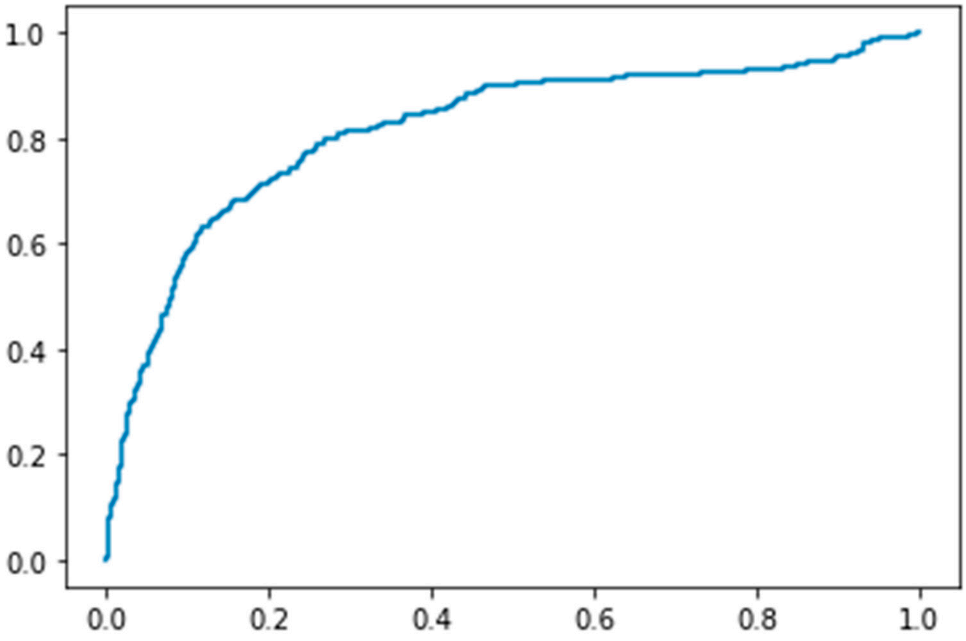

Figure S9: ROC curve of adaboost classifier

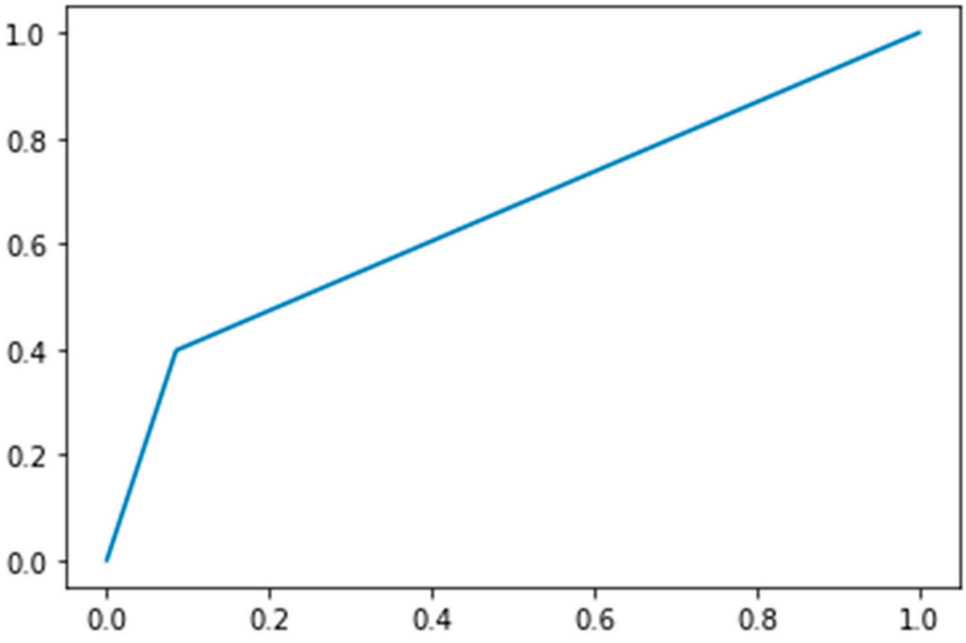

Figure S10: ROC curve of decision tree classifier

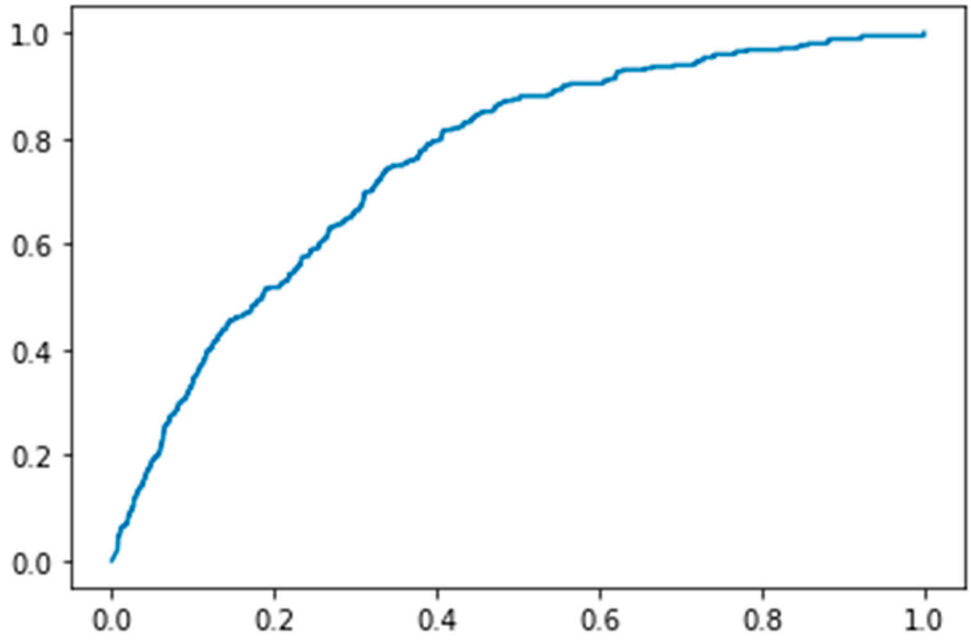

Figure S11: ROC curve of Navie bayes classifier

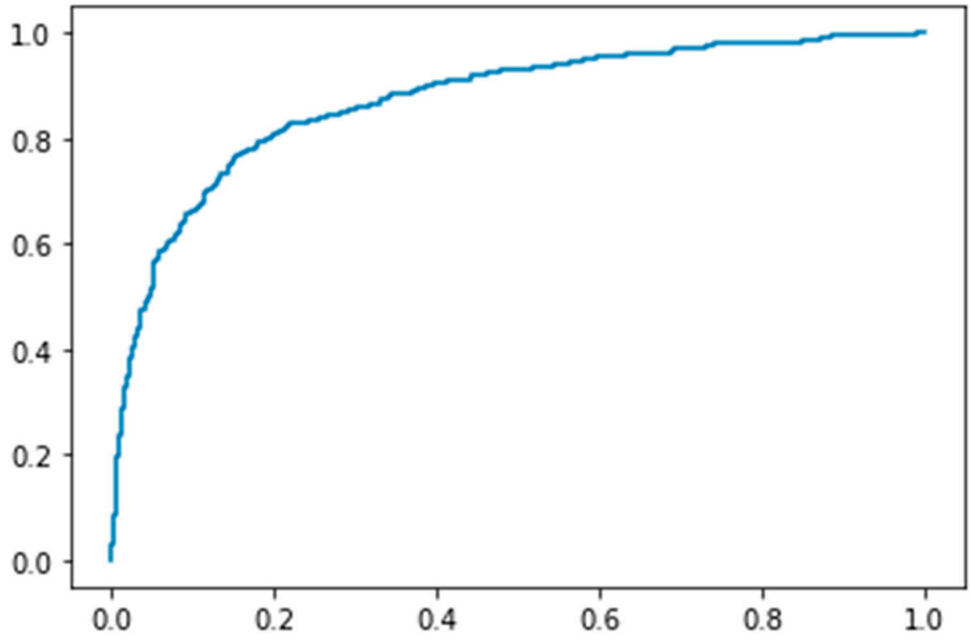

Figure S12: ROC curve of gradient boost classifier

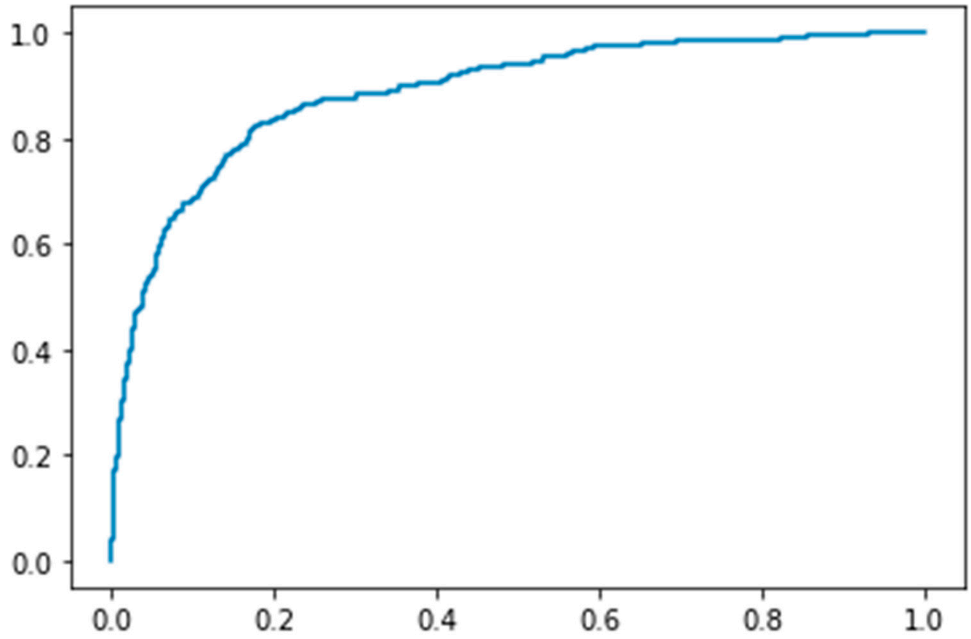

Figure S13: ROC curve of light gradient boost classifier

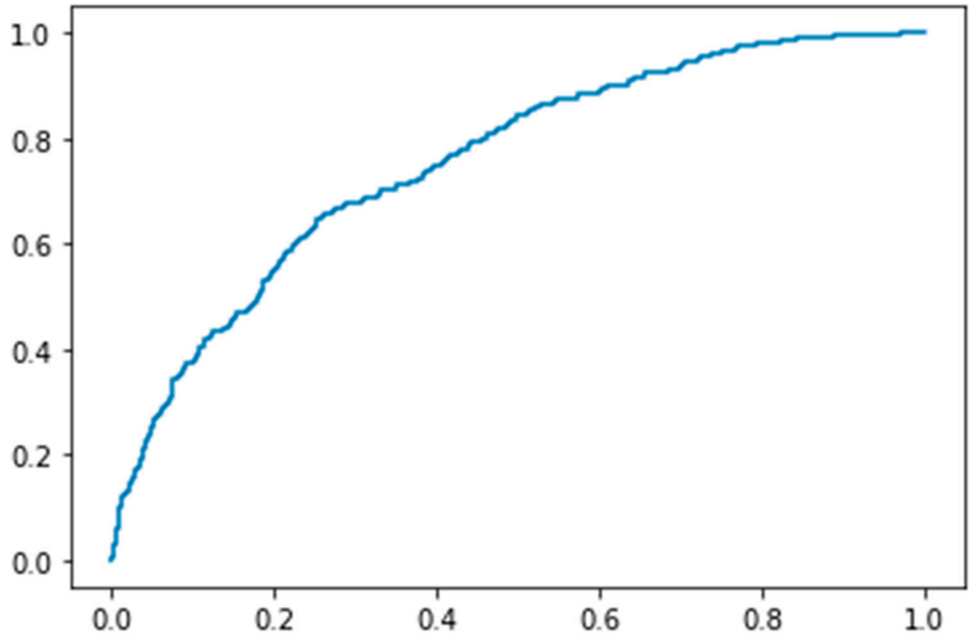

Figure S14: ROC curve of logistic regression classifier

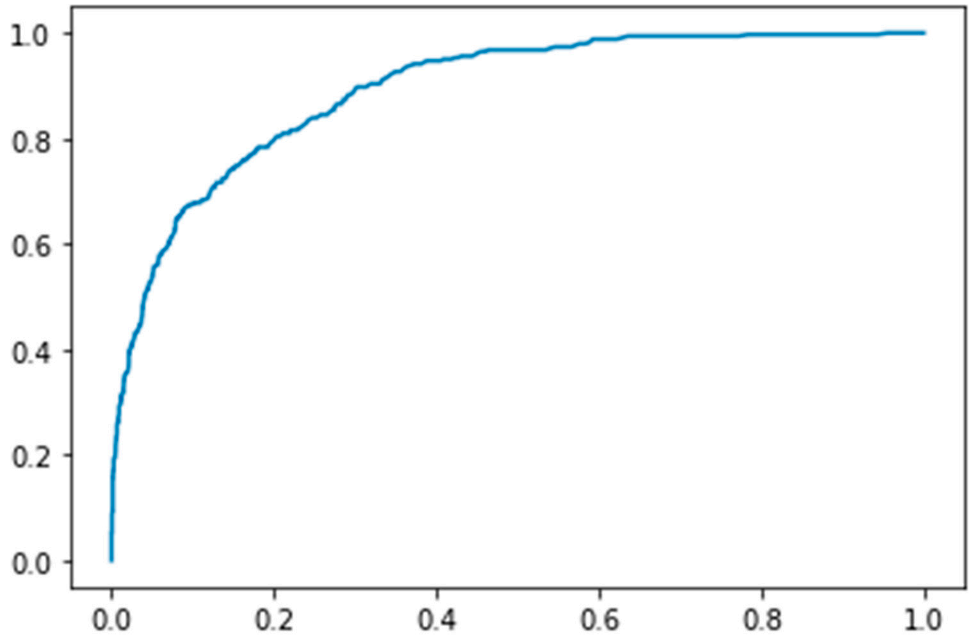

Figure S15: ROC curve of random forest classifier

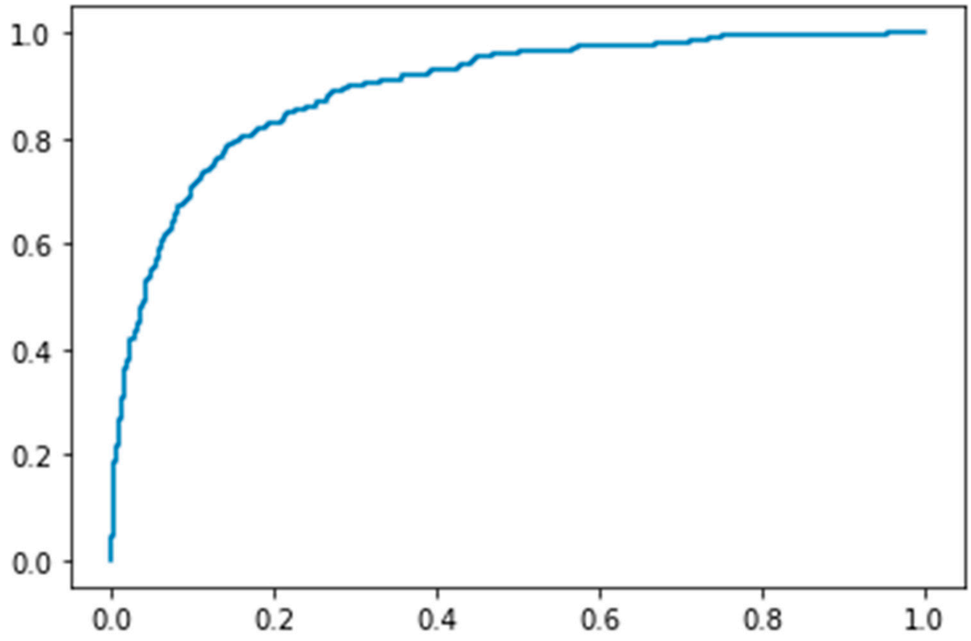

Figure S16: ROC curve of xgboost classifier

Python codes

```
from sklearn.ensemble import AdaBoostClassifier
import pandas as pd
import numpy as np
import matplotlib.pyplot as plt
from sklearn.model_selection import train_test_split
from sklearn.model_selection import cross_val_score
from sklearn.model_selection import cross_val_predict
from sklearn.metrics import auc
from sklearn.metrics import roc_curve
from sklearn.metrics import accuracy_score
from sklearn.metrics import precision_score
from sklearn.metrics import recall_score
from sklearn.metrics import f1_score
tbi =  pd.read_csv('/home/guoyr/Desktop/project/trauma/uniontable/ML/tbiml.csv')
X = tbi.loc[:,'gender':'Neurosurgery']
Y = tbi.flag
X_train, X_test, y_train, y_test = train_test_split(X, Y, stratify=Y, random_state=42)
model = AdaBoostClassifier(n_estimators=1000,learning_rate = 0.5,random_state=0)
cross_val_score(model,X,Y,cv=10,scoring="accuracy")
y_probas_ada = cross_val_predict(model,X,Y,cv=10,method="predict_proba")
y_pred_ada = np.around(y_scores_ada,0).astype(int)
precision_score(Y,y_pred_ada)
recall_score(Y,y_pred_ada)
f1_score(Y,y_pred_ada)
auc(fpr_ada,tpr_ada)
y_probas_ada_cc = y_probas_ada[:,1]
fpr_ada, tpr_ada, threshold_ada = roc_curve(Y,y_probas_ada_cc)
plt.plot(fpr_ada,tpr_aada)
model.fit(X_train, y_train)
feature_importances = model.feature_importances_
# 创建特征名列表
feature_names = list(X.columns)
# 创建一个 DataFrame，包含特征名和其重要性得分
feature_importances_df = pd.DataFrame({'feature': feature_names, 'importance':
feature_importances})
# 对特征重要性得分进行排序
feature_importances_df = feature_importances_df.sort_values('importance', ascending=False)

# 颜色映射
colors = plt.cm.viridis(np.linspace(0, 1, len(feature_names)))
```

```
# 可视化特征重要性
fig, ax = plt.subplots(figsize=(20, 12))
ax.barh(feature_importances_df['feature'], feature_importances_df['importance'])
ax.invert_yaxis()  # 翻转 y 轴，使得最大的特征在最上面
ax.set_xlabel('IMPORTANCE', fontsize=12)  # 图形的 x 标签
ax.set_title('AdaboostClassifier Importance',fontsize=16)
for i, v in enumerate(feature_importances_df['importance']):
    ax.text(v + 0.01, i, str(round(v, 3)), va='center', fontname='Times New Roman', fontsize=10)

## 设置图形样式
# plt.style.use('default')
ax.spines['top'].set_visible(False)  # 去掉上边框
ax.spines['right'].set_visible(False)  # 去掉右边框
# ax.spines['left'].set_linewidth(0.5)#左边框粗细
# ax.spines['bottom'].set_linewidth(0.5)#下边框粗细
# ax.tick_params(width=0.5)
# ax.set_facecolor('white')#背景色为白色
# ax.grid(False)#关闭内部网格线

# 保存图形
plt.savefig('./特征重要性.jpg', dpi=400, bbox_inches='tight')



from sklearn import tree
import pandas as pd
import numpy as np
import matplotlib.pyplot as plt
from sklearn.model_selection import train_test_split
from sklearn.model_selection import cross_val_score
from sklearn.model_selection import cross_val_predict
from sklearn.metrics import auc
from sklearn.metrics import roc_curve
from sklearn.metrics import accuracy_score
from sklearn.metrics import precision_score
from sklearn.metrics import recall_score
from sklearn.metrics import f1_score
tbi =  pd.read_csv('/home/guoyr/Desktop/project/trauma/uniontable/ML/tbiml.csv')
X = tbi.loc[:,'gender':'Neurosurgery']
Y = tbi.flag
X_train, X_test, y_train, y_test = train_test_split(X, Y, stratify=Y, random_state=42)
clf=tree.DecisionTreeClassifier(random_state=0)
cross_val_score(clf,X,Y,cv=10,scoring="accuracy")
y_probas_clf = cross_val_predict(clf,X,Y,cv=10,method="predict_proba")
```

```
y_probas_clf_cc = y_probas_clf[:,1]
y_pred_clf = np.around(y_probas_clf_cc,0).astype(int)
precision_score(Y,y_pred_clf)
recall_score(Y,y_pred_clf)
f1_score(Y,y_pred_clf)
fpr_clf, tpr_clf, threshold_clf = roc_curve(Y,y_probas_clf_cc)
plt.plot(fpr_clf,tpr_clf)
auc(fpr_clf,tpr_clf)
clf.fit(X_train, y_train)
feature_importances = clf.feature_importances_
# 创建特征名列表
feature_names = list(X.columns)
# 创建一个 DataFrame，包含特征名和其重要性得分
feature_importances_df = pd.DataFrame({'feature': feature_names, 'importance':
feature_importances})
# 对特征重要性得分进行排序
feature_importances_df = feature_importances_df.sort_values('importance', ascending=False)

# 颜色映射
colors = plt.cm.viridis(np.linspace(0, 1, len(feature_names)))

# 可视化特征重要性
fig, ax = plt.subplots(figsize=(20, 12))
ax.barh(feature_importances_df['feature'], feature_importances_df['importance'])
ax.invert_yaxis()  # 翻转 y 轴，使得最大的特征在最上面
ax.set_xlabel('IMPORTANCE', fontsize=12)  # 图形的 x 标签
ax.set_title('DecisionTreeClassifier Importance',fontsize=16)
for i, v in enumerate(feature_importances_df['importance']):
    ax.text(v + 0.01, i, str(round(v, 3)), va='center', fontname='Times New Roman', fontsize=10)

# # 设置图形样式
# plt.style.use('default')
ax.spines['top'].set_visible(False)  # 去掉上边框
ax.spines['right'].set_visible(False)  # 去掉右边框
# ax.spines['left'].set_linewidth(0.5)#左边框粗细
# ax.spines['bottom'].set_linewidth(0.5)#下边框粗细
# ax.tick_params(width=0.5)
# ax.set_facecolor('white')#背景色为白色
# ax.grid(False)#关闭内部网格线

# 保存图形
plt.savefig('./特征重要性.jpg', dpi=400, bbox_inches='tight')
```

```
from sklearn.ensemble import GradientBoostingClassifier
from sklearn.inspection import permutation_importance
from sklearn.naive_bayes import GaussianNB
import pandas as pd
import numpy as np
import matplotlib.pyplot as plt
from sklearn.model_selection import train_test_split
from sklearn.model_selection import cross_val_score
from sklearn.model_selection import cross_val_predict
from sklearn.metrics import auc
from sklearn.metrics import roc_curve
from sklearn.metrics import accuracy_score
from sklearn.metrics import precision_score
from sklearn.metrics import recall_score
from sklearn.metrics import f1_score
tbi =   pd.read_csv('/home/guoyr/Desktop/project/trauma/uniontable/ML/tbiml.csv')
X = tbi.loc[:,'gender':'Neurosurgery']
Y = tbi.flag
X_train, X_test, y_train, y_test = train_test_split(X, Y, stratify=Y, random_state=42)
model = GaussianNB(priors=None)
cross_val_score(model,X,Y,cv=10,scoring="accuracy")
y_probas_ada = cross_val_predict(model,X,Y,cv=10,method="predict_proba")
y_probas_ada_cc = y_probas_ada[:,1]
y_pred_ada = np.around(y_probas_ada_cc,0).astype(int)
precision_score(Y,y_pred_ada)
recall_score(Y,y_pred_ada)
f1_score(Y,y_pred_ada)
fpr_ada, tpr_ada, threshold_ada = roc_curve(Y,y_probas_ada_cc)
plt.plot(fpr_ada,tpr_ada)
auc(fpr_ada,tpr_ada)
model.fit(X_train, y_train)
imps = permutation_importance(model, X_test, y_test)
feature_importances = imps.importances_mean.reshape(-1)
# 创建特征名列
feature_names = list(X.columns)
# 创建一个 DataFrame，包含特征名和其重要性得分
feature_importances_df = pd.DataFrame({'feature': feature_names, 'importance':
feature_importances})
# 对特征重要性得分进行排序
feature_importances_df = feature_importances_df.sort_values('importance', ascending=False)

# 颜色映射
```

```
colors = plt.cm.viridis(np.linspace(0, 1, len(feature_names)))

# 可视化特征重要性
fig, ax = plt.subplots(figsize=(20, 12))
ax.barh(feature_importances_df['feature'], feature_importances_df['importance'])
ax.invert_yaxis()  # 翻转 y 轴，使得最大的特征在最上面
ax.set_xlabel('IMPORTANCE', fontsize=12)  # 图形的 x 标签
ax.set_title('GaussianNB Importance',fontsize=16)
for i, v in enumerate(feature_importances_df['importance']):
    ax.text(v + 0.01, i, str(round(v, 3)), va='center', fontname='Times New Roman', fontsize=10)

# # 设置图形样式
# plt.style.use('default')
ax.spines['top'].set_visible(False)  # 去掉上边框
ax.spines['right'].set_visible(False)  # 去掉右边框
# ax.spines['left'].set_linewidth(0.5)#左边框粗细
# ax.spines['bottom'].set_linewidth(0.5)#下边框粗细
# ax.tick_params(width=0.5)
# ax.set_facecolor('white')#背景色为白色
# ax.grid(False)#关闭内部网格线

# 保存图形
plt.savefig('./特征重要性.jpg', dpi=400, bbox_inches='tight')


from sklearn.ensemble import GradientBoostingClassifier
import pandas as pd
import numpy as np
import matplotlib.pyplot as plt
from sklearn.model_selection import train_test_split
from sklearn.model_selection import cross_val_score
from sklearn.model_selection import cross_val_predict
from sklearn.metrics import auc
from sklearn.metrics import roc_curve
from sklearn.metrics import accuracy_score
from sklearn.metrics import precision_score
from sklearn.metrics import recall_score
from sklearn.metrics import f1_score
tbi =  pd.read_csv('/home/guoyr/Desktop/project/trauma/uniontable/ML/tbiml.csv')
X = tbi.loc[:,'gender':'Neurosurgery']
Y = tbi.flag
X_train, X_test, y_train, y_test = train_test_split(X, Y, stratify=Y, random_state=42)
model = GradientBoostingClassifier(n_estimators=1000,learning_rate = 0.5,random_state=0)
```

```
cross_val_score(model,X,Y,cv=10,scoring="accuracy")
y_probas_ada = cross_val_predict(model,X,Y,cv=10,method="predict_proba")
y_probas_ada_cc = y_probas_ada[:,1]
y_pred_ada = np.around(y_probas_ada_cc,0).astype(int)
precision_score(Y,y_pred_ada)
recall_score(Y,y_pred_ada)
f1_score(Y,y_pred_ada)
fpr_ada, tpr_ada, threshold_ada = roc_curve(Y,y_probas_ada_cc)
plt.plot(fpr_ada,tpr_ada)
auc(fpr_ada,tpr_ada)
model.fit(X_train, y_train)
feature_importances = model.feature_importances_
# 创建特征名列表
feature_names = list(X.columns)
# 创建一个 DataFrame，包含特征名和其重要性得分
feature_importances_df = pd.DataFrame({'feature': feature_names, 'importance':
feature_importances})
# 对特征重要性得分进行排序
feature_importances_df = feature_importances_df.sort_values('importance', ascending=False)

# 颜色映射
colors = plt.cm.viridis(np.linspace(0, 1, len(feature_names)))

# 可视化特征重要性
fig, ax = plt.subplots(figsize=(20, 12))
ax.barh(feature_importances_df['feature'], feature_importances_df['importance'])
ax.invert_yaxis()  # 翻转 y 轴，使得最大的特征在最上面
ax.set_xlabel('IMPORTANCE', fontsize=12)  # 图形的 x 标签
ax.set_title('GradientboostClassifier Importance',fontsize=16)
for i, v in enumerate(feature_importances_df['importance']):
    ax.text(v + 0.01, i, str(round(v, 3)), va='center', fontname='Times New Roman', fontsize=10)

## 设置图形样式
# plt.style.use('default')
ax.spines['top'].set_visible(False)  # 去掉上边框
ax.spines['right'].set_visible(False)  # 去掉右边框
# ax.spines['left'].set_linewidth(0.5)#左边框粗细
# ax.spines['bottom'].set_linewidth(0.5)#下边框粗细
# ax.tick_params(width=0.5)
# ax.set_facecolor('white')#背景色为白色
# ax.grid(False)#关闭内部网格线

# 保存图形
```

```
plt.savefig('./特征重要性.jpg', dpi=400, bbox_inches='tight')
import lightgbm as lgb
import pandas as pd
import numpy as np
import matplotlib.pyplot as plt
from sklearn.model_selection import train_test_split
from sklearn.model_selection import cross_val_score
from sklearn.model_selection import cross_val_predict
from sklearn.metrics import auc
from sklearn.metrics import roc_curve
from sklearn.metrics import accuracy_score
from sklearn.metrics import precision_score
from sklearn.metrics import recall_score
from sklearn.metrics import f1_score
tbi =   pd.read_csv('/home/guoyr/Desktop/project/trauma/uniontable/ML/tbiml.csv')
X = tbi.loc[:,'gender':'Neurosurgery']
Y = tbi.flag
X_train, X_test, y_train, y_test = train_test_split(X, Y, stratify=Y, random_state=42)
model = lgb.LGBMClassifier(n_estimators=1000,learning_rate = 0.5,random_state=0)
cross_val_score(model,X,Y,cv=10,scoring="accuracy")
y_probas_ada = cross_val_predict(model,X,Y,cv=10,method="predict_proba")
y_probas_ada_cc = y_probas_ada[:,1]
y_pred_ada = np.around(y_probas_ada_cc,0).astype(int)
precision_score(Y,y_pred_ada)
recall_score(Y,y_pred_ada)
f1_score(Y,y_pred_ada)
fpr_ada, tpr_ada, threshold_ada = roc_curve(Y,y_probas_ada_cc)
plt.plot(fpr_ada,tpr_ada)
auc(fpr_ada,tpr_ada)
model.fit(X_train, y_train)
feature_importances = model.feature_importances_
# 创建特征名列表
feature_names = list(X.columns)
# 创建一个 DataFrame，包含特征名和其重要性得分
feature_importances_df = pd.DataFrame({'feature': feature_names, 'importance':
feature_importances})
# 对特征重要性得分进行排序
feature_importances_df = feature_importances_df.sort_values('importance', ascending=False)

# 颜色映射
colors = plt.cm.viridis(np.linspace(0, 1, len(feature_names)))

# 可视化特征重要性
```

```
fig, ax = plt.subplots(figsize=(20, 12))
ax.barh(feature_importances_df['feature'], feature_importances_df['importance'])
ax.invert_yaxis()  # 翻转 y 轴，使得最大的特征在最上面
ax.set_xlabel('IMPORTANCE', fontsize=12)  # 图形的 x 标签
ax.set_title('Light Gradient Boost Classifier Importance',fontsize=16)
for i, v in enumerate(feature_importances_df['importance']):
    ax.text(v + 0.01, i, str(round(v, 3)), va='center', fontname='Times New Roman', fontsize=10)

# # 设置图形样式
# plt.style.use('default')
ax.spines['top'].set_visible(False)  # 去掉上边框
ax.spines['right'].set_visible(False)  # 去掉右边框
# ax.spines['left'].set_linewidth(0.5)#左边框粗细
# ax.spines['bottom'].set_linewidth(0.5)#下边框粗细
# ax.tick_params(width=0.5)
# ax.set_facecolor('white')#背景色为白色
# ax.grid(False)#关闭内部网格线

# 保存图形
plt.savefig('./特征重要性.jpg', dpi=400, bbox_inches='tight')

from sklearn.linear_model import LogisticRegression
import pandas as pd
import numpy as np
import matplotlib.pyplot as plt
from sklearn.model_selection import train_test_split
from sklearn.model_selection import cross_val_score
from sklearn.model_selection import cross_val_predict
from sklearn.metrics import auc
from sklearn.metrics import roc_curve
from sklearn.metrics import accuracy_score
from sklearn.metrics import precision_score
from sklearn.metrics import recall_score
from sklearn.metrics import f1_score
tbi =   pd.read_csv('/home/guoyr/Desktop/project/trauma/uniontable/ML/tbiml.csv')
X = tbi.loc[:,'gender':'Neurosurgery']
Y = tbi.flag
X_train, X_test, y_train, y_test = train_test_split(X, Y, stratify=Y, random_state=42)
model=LogisticRegression()
cross_val_score(model,X,Y,cv=10,scoring="accuracy")
y_probas_ada = cross_val_predict(model,X,Y,cv=10,method="predict_proba")
y_probas_ada_cc = y_probas_ada[:,1]
y_pred_ada = np.around(y_probas_ada_cc,0).astype(int)
```

```
precision_score(Y,y_pred_ada)
recall_score(Y,y_pred_ada)
f1_score(Y,y_pred_ada)
fpr_ada, tpr_ada, threshold_ada = roc_curve(Y,y_probas_ada_cc)
plt.plot(fpr_ada,tpr_ada)
auc(fpr_ada,tpr_ada)
model.fit(X_train, y_train)
model.coef_
coef = model.coef_.reshape(-1)
feature_importances = coef
# 创建特征名列表
feature_names = list(X.columns)
# 创建一个 DataFrame，包含特征名和其重要性得分
feature_importances_df = pd.DataFrame({'feature': feature_names, 'importance':
feature_importances})
# 对特征重要性得分进行排序
feature_importances_df = feature_importances_df.sort_values('importance', ascending=False)

# 颜色映射
colors = plt.cm.viridis(np.linspace(0, 1, len(feature_names)))

# 可视化特征重要性
fig, ax = plt.subplots(figsize=(20, 12))
ax.barh(feature_importances_df['feature'], feature_importances_df['importance'])
ax.invert_yaxis()  # 翻转 y 轴，使得最大的特征在最上面
ax.set_xlabel('IMPORTANCE', fontsize=12)  # 图形的 x 标签
ax.set_title('LogisticRegressionClassifier Importance',fontsize=16)
for i, v in enumerate(feature_importances_df['importance']):
    ax.text(v + 0.01, i, str(round(v, 3)), va='center', fontname='Times New Roman', fontsize=10)

## 设置图形样式
# plt.style.use('default')
ax.spines['top'].set_visible(False)  # 去掉上边框
ax.spines['right'].set_visible(False)  # 去掉右边框
# ax.spines['left'].set_linewidth(0.5)#左边框粗细
# ax.spines['bottom'].set_linewidth(0.5)#下边框粗细
# ax.tick_params(width=0.5)
# ax.set_facecolor('white')#背景色为白色
# ax.grid(False)#关闭内部网格线

# 保存图形
plt.savefig('./特征重要性.jpg', dpi=400, bbox_inches='tight')
```

```
from sklearn.ensemble import RandomForestClassifier        #引入随机森林分类器
import pandas as pd
import numpy as np
import matplotlib.pyplot as plt
from sklearn.model_selection import train_test_split
from sklearn.model_selection import cross_val_score
from sklearn.model_selection import cross_val_predict
from sklearn.metrics import auc
from sklearn.metrics import roc_curve
from sklearn.metrics import accuracy_score
from sklearn.metrics import precision_score
from sklearn.metrics import recall_score
from sklearn.metrics import f1_score
tbi =   pd.read_csv('/home/guoyr/Desktop/project/trauma/uniontable/ML/tbiml.csv')
X = tbi.loc[:,'gender':'Neurosurgery']
Y = tbi.flag
X_train, X_test, y_train, y_test = train_test_split(X, Y, stratify=Y, random_state=42)

model = RandomForestClassifier(n_estimators=1000, random_state=0)
cross_val_score(model,X,Y,cv=10,scoring="accuracy")
y_probas_ada = cross_val_predict(model,X,Y,cv=10,method="predict_proba")
y_probas_ada_cc = y_probas_ada[:,1]
y_pred_ada = np.around(y_probas_ada_cc,0).astype(int)
precision_score(Y,y_pred_ada)
recall_score(Y,y_pred_ada)
f1_score(Y,y_pred_ada)
fpr_ada, tpr_ada, threshold_ada = roc_curve(Y,y_probas_ada_cc)
plt.plot(fpr_ada,tpr_ada)
auc(fpr_ada,tpr_ada)
model.fit(X_train, y_train)
feature_importances = model.feature_importances_
# 创建特征名列表
feature_names = list(X.columns)
# 创建一个 DataFrame，包含特征名和其重要性得分
feature_importances_df = pd.DataFrame({'feature': feature_names, 'importance':
feature_importances})
# 对特征重要性得分进行排序
feature_importances_df = feature_importances_df.sort_values('importance', ascending=False)

# 颜色映射
#colors = plt.cm.viridis(np.linspace(0, 1, len(feature_names)))

# 可视化特征重要性
```

```
fig, ax = plt.subplots(figsize=(20, 12))
ax.barh(feature_importances_df['feature'], feature_importances_df['importance'])
ax.invert_yaxis()  # 翻转 y 轴，使得最大的特征在最上面
ax.set_xlabel('IMPORTANCE', fontsize=12)  # 图形的 x 标签
ax.set_title('RandomForestClassifier Importance',fontsize=16)
for i, v in enumerate(feature_importances_df['importance']):
    ax.text(v + 0.01, i, str(round(v, 3)), va='center', fontname='Times New Roman', fontsize=10)

# # 设置图形样式
# plt.style.use('default')
ax.spines['top'].set_visible(False)  # 去掉上边框
ax.spines['right'].set_visible(False)  # 去掉右边框
# ax.spines['left'].set_linewidth(0.5)#左边框粗细
# ax.spines['bottom'].set_linewidth(0.5)#下边框粗细
# ax.tick_params(width=0.5)
# ax.set_facecolor('white')#背景色为白色
# ax.grid(False)#关闭内部网格线

# 保存图形
plt.savefig('./特征重要性.jpg', dpi=400, bbox_inches='tight')

from xgboost import XGBClassifier
from xgboost import plot_importance
import pandas as pd
import numpy as np
import matplotlib.pyplot as plt
from sklearn.model_selection import train_test_split
from sklearn.model_selection import cross_val_score
from sklearn.model_selection import cross_val_predict
from sklearn.metrics import auc
from sklearn.metrics import roc_curve
from sklearn.metrics import accuracy_score
from sklearn.metrics import precision_score
from sklearn.metrics import recall_score
from sklearn.metrics import f1_score
tbi =  pd.read_csv('/home/guoyr/Desktop/project/trauma/uniontable/ML/tbiml.csv')
X = tbi.loc[:,'gender':'Neurosurgery']
Y = tbi.flag
X_train, X_test, y_train, y_test = train_test_split(X, Y, stratify=Y, random_state=42)
model = XGBClassifier(learning_rate=0.1,
                      n_estimators=1000,          # 树的个数--100 棵树建立 xgboost
                                   # 树的深度
                      min_child_weight = 1,       # 叶子节点最小权重
```

```
                    gamma=0.,                # 惩罚项中叶子结点个数前的参数
                    subsample=0.8,           # 随机选择 80%样本建立决策树
                    colsample_btree=0.8,     # 随机选择 80%特征建立决策树
                                            # 指定损失函数
                    scale_pos_weight=1,      # 解决样本个数不平衡的问题
                    random_state=27          # 随机数
                    )
cross_val_score(model,X,Y,cv=10,scoring="accuracy")
y_probas_ada = cross_val_predict(model,X,Y,cv=10,method="predict_proba")
y_probas_ada_cc = y_probas_ada[:,1]
y_pred_ada = np.around(y_probas_ada_cc,0).astype(int)
precision_score(Y,y_pred_ada)
recall_score(Y,y_pred_ada)
f1_score(Y,y_pred_ada)
fpr_ada, tpr_ada, threshold_ada = roc_curve(Y,y_probas_ada_cc)
plt.plot(fpr_ada,tpr_ada)
auc(fpr_ada,tpr_ada)
model.fit(X_train, y_train)
feature_importances = model.feature_importances_
# 创建特征名列表
feature_names = list(X.columns)
# 创建一个 DataFrame，包含特征名和其重要性得分
feature_importances_df = pd.DataFrame({'feature': feature_names, 'importance':
feature_importances})
# 对特征重要性得分进行排序
feature_importances_df = feature_importances_df.sort_values('importance', ascending=False)

# 颜色映射
colors = plt.cm.viridis(np.linspace(0, 1, len(feature_names)))

# 可视化特征重要性
fig, ax = plt.subplots(figsize=(20, 12))
ax.barh(feature_importances_df['feature'], feature_importances_df['importance'])
ax.invert_yaxis()  # 翻转 y 轴，使得最大的特征在最上面
ax.set_xlabel('IMPORTANCE', fontsize=12)  # 图形的 x 标签
ax.set_title('XGboostClassifier Importance',fontsize=16)
for i, v in enumerate(feature_importances_df['importance']):
    ax.text(v + 0.01, i, str(round(v, 3)), va='center', fontname='Times New Roman', fontsize=10)

## 设置图形样式
# plt.style.use('default')
ax.spines['top'].set_visible(False)  # 去掉上边框
ax.spines['right'].set_visible(False)  # 去掉右边框
```

```
# ax.spines['left'].set_linewidth(0.5)#左边框粗细
# ax.spines['bottom'].set_linewidth(0.5)#下边框粗细
# ax.tick_params(width=0.5)
# ax.set_facecolor('white')#背景色为白色
# ax.grid(False)#关闭内部网格线

# 保存图形
plt.savefig('./特征重要性.jpg', dpi=400, bbox_inches='tight')

### 特征重要性
fig,ax = plt.subplots(figsize=(15,15))
plot_importance(model, height=0.5, ax=ax, importance_type = "weight")
plt.show()

### 预测
y_pred = model.predict(X_test)

### 模型正确率
accuracy = accuracy_score(y_test,y_pred)
print(accuracy)
```

SQL codes

```
-- THIS SCRIPT IS AUTOMATICALLY GENERATED. DO NOT EDIT IT DIRECTLY.
create table alb_first_time as
with tb1 as (SELECT
    hadm_id
  , charttime

  -- Albumin   Blood
  , MAX(CASE WHEN itemid in (50862) THEN valuenum ELSE NULL END) AS alb

FROM mimiciii.labevents le
WHERE le.itemid IN
( 50862 -- Albumin   Blood

)
AND valuenum IS NOT NULL
-- differential values cannot be negative
AND valuenum >= 0
AND hadm_id IS NOT NULL
GROUP BY   hadm_id,charttime
ORDER BY   hadm_id,charttime

--63   50862 Albumin Blood Chemistry
)

,tb2 as (select
hadm_id
,charttime
,alb
,ROW_NUMBER() OVER (PARTITION BY hadm_id ORDER BY charttime)
from tb1)

select
hadm_id
,alb
from tb2
where row_number = 1


DROP TABLE IF EXISTS bg_first_day_art; create table bg_first_day_art as
SELECT
hadm_id
```

```
,icustay_id
--,CAST(charttime as DATE) as chartdate
,MAX(so2) as so2_max
,MIN(so2) as so2_min
,MAX(spo2) as spo2_max
,MIN(spo2) as spo2_min
,MAX(po2) as po2_max
,MIN(po2) as po2_min
,MAX(pco2) as pco2_max
,MIN(pco2) as pco2_min
,MAX(aado2) as aado2_max
,MIN(aado2) as aado2_min
,MAX(aado2_calc) as aado2_calc_max
,MIN(aado2_calc) as aado2_calc_min
,MAX(pao2fio2) as pao2fio2_max
,MIN(pao2fio2) as pao2fio2_min
,MAX(ph) as ph_max
,MIN(ph) as ph_min
,MAX(baseexcess) as baseexcess_max
,MIN(baseexcess) as baseexcess_min
,MAX(bicarbonate) as bicarbonate_max
,MIN(bicarbonate) as bicarbonate_min
,MAX(totalco2) totalco2_max
,MIN(totalco2) totalco2_min
,MAX(hematocrit) hematocrit_max
,MIN(hematocrit) hematocrit_min
,MAX(hemoglobin) hemoglobin_max
,MIN(hemoglobin) hemoglobin_min
,MAX(chloride) as chloride_max
,MIN(chloride) as chloride_min
,MAX(calcium) as calcium_max
,MIN(calcium) as calcium_min
,MAX(temperature) as temperature_max
,MIN(temperature) as temperature_min
,MAX(potassium) as potassium_max
,MIN(potassium) as potassium_min
,MAX(sodium) as sodium_max
,MIN(sodium) as sodium_min
,MAX(lactate) as lactate_max
,MIN(lactate) as lactate_min
from blood_gas_first_day_arterial
group by
hadm_id
```

```
,icustay_id
--,CAST(charttime as DATE)
order by
hadm_id
,icustay_id

-- THIS SCRIPT IS AUTOMATICALLY GENERATED. DO NOT EDIT IT DIRECTLY.
DROP TABLE IF EXISTS blood_differential; CREATE TABLE blood_differential AS
-- For reference, some common unit conversions:
-- 10^9/L == K/uL == 10^3/uL
WITH blood_diff AS
(
SELECT
    subject_id
   ,hadm_id
  , charttime
  -- create one set of columns for percentages, and one set of columns for counts
  -- we harmonize all count units into K/uL == 10^9/L
  -- counts have an "_abs" suffix, percentages do not

  -- absolute counts
  , MAX(CASE WHEN itemid in (51300, 51301) THEN valuenum ELSE NULL END) AS wbc
  , MAX(CASE WHEN itemid = 51133 THEN valuenum   ELSE NULL END) AS lymphocytes_abs
  -- 52074 in K/uL, 51253 in #/uL
  , MAX(CASE WHEN itemid = 51253 THEN valuenum / 1000.0 ELSE NULL END) AS
monocytes_abs
  , MAX(CASE WHEN itemid = 51218 THEN valuenum / 1000.0 ELSE NULL END) AS
granulocytes_abs
  -- percentages, equal to cell count / white blood cell count
  , MAX(CASE WHEN itemid = 51146 THEN valuenum ELSE NULL END) AS basophils
  , MAX(CASE WHEN itemid = 51200 THEN valuenum ELSE NULL END) AS eosinophils
  , MAX(CASE WHEN itemid in (51244, 51245) THEN valuenum ELSE NULL END) AS lymphocytes
  , MAX(CASE WHEN itemid = 51254 THEN valuenum ELSE NULL END) AS monocytes
  , MAX(CASE WHEN itemid = 51256 THEN valuenum ELSE NULL END) AS neutrophils
  , MAX(CASE WHEN itemid = 51277 THEN valuenum ELSE NULL END) AS rdw
  -- other cell count percentages
  , MAX(CASE WHEN itemid = 51143 THEN valuenum ELSE NULL END) AS atypical_lymphocytes
  , MAX(CASE WHEN itemid = 51144 THEN valuenum ELSE NULL END) AS bands
  , MAX(CASE WHEN itemid = 51251 THEN valuenum ELSE NULL END) AS metamyelocytes
  , MAX(CASE WHEN itemid = 51257 THEN valuenum ELSE NULL END) AS nrbc
  -- utility flags which determine whether imputation is possible
  , CASE
    -- WBC is available
```

```
    WHEN MAX(CASE WHEN itemid in (51300, 51301) THEN valuenum ELSE NULL END) > 0
    -- and we have at least one percentage from the diff
    -- sometimes the entire diff is 0%, which looks like bad data
    AND SUM(CASE WHEN itemid IN (51146, 51200, 51244, 51245, 51254, 51256) THEN
valuenum ELSE NULL END) > 0
    THEN 1 ELSE 0 END AS impute_abs
FROM mimiciii.labevents le
WHERE le.itemid IN
(
    51146, -- basophils
    51199, -- Eosinophil Count
    51200, -- Eosinophils
    51244, -- Lymphocytes
    51245, -- Lymphocytes, Percent
    51133, -- Absolute Lymphocyte Count
    51253, -- Monocyte Count
    51254, -- Monocytes
    51256, -- Neutrophils
    51143, -- Atypical lymphocytes
    51144, -- Bands (%)
    51218, -- Granulocyte Count
    51251, -- Metamyelocytes
    51257, -- Nucleated Red Cells
    51277, -- rdw
    -- wbc totals measured in K/uL
    51300, 51301
    -- 52220 (wbcp) is percentage

    -- below are point of care tests which are extremely infrequent and usually low quality
    -- 51697, -- Neutrophils (mmol/L)

    -- below itemid do not have data as of MIMIC-IV v1.0
    -- 51536, -- Absolute Lymphocyte Count
    -- 51537, -- Absolute Neutrophil
    -- 51690, -- Lymphocytes
    -- 52151, -- NRBC
)
AND valuenum IS NOT NULL
-- differential values cannot be negative
AND valuenum >= 0
GROUP BY   subject_id,hadm_id,charttime
)
SELECT
```

```
subject_id, hadm_id, charttime
, wbc
-- impute absolute count if percentage & WBC is available
, ROUND( CAST( CASE
    WHEN   basophils IS NOT NULL AND impute_abs = 1
        THEN basophils * wbc / 100
    ELSE NULL
END as numeric),4) AS basophils_abs
, ROUND( CAST( CASE
    WHEN   eosinophils IS NOT NULL AND impute_abs = 1
        THEN eosinophils * wbc / 100
    ELSE NULL
END as numeric),4) AS eosinophils_abs
, ROUND( CAST( CASE
    WHEN lymphocytes_abs IS NULL AND lymphocytes IS NOT NULL AND impute_abs = 1
        THEN lymphocytes * wbc / 100
    ELSE lymphocytes_abs
END as numeric),4) AS lymphocytes_abs
, ROUND( CAST( CASE
    WHEN monocytes_abs IS NULL AND monocytes IS NOT NULL AND impute_abs = 1
        THEN monocytes * wbc / 100
    ELSE monocytes_abs
END as numeric),4) AS monocytes_abs
, ROUND( CAST( CASE
    WHEN   neutrophils IS NOT NULL AND impute_abs = 1
        THEN neutrophils * wbc / 100
    ELSE NULL
END as numeric),4) AS neutrophils_abs

, basophils
, eosinophils
, lymphocytes
, monocytes
, neutrophils
, rdw

-- impute bands/blasts?
, atypical_lymphocytes
, bands
, metamyelocytes
, nrbc
FROM blood_diff
where hadm_id is not null
```

```
ORDER BY   subject_id,hadm_id,charttime
;

with base as (select ce.hadm_id, ce.icustay_id, ce.charttime
, max(case when itemid in (1127, 861, 1542) then valuenum else null end) as wbc
, max(case when itemid in (797) then valuenum else null end) as eosinophils
, max(case when itemid in (796) then valuenum else null end) as basophils
, max(case when itemid in (798) then valuenum else null end) as lymphocytes
, max(case when itemid in (799) then valuenum else null end) as monocytes
, max(case when itemid in (833) then valuenum else null end) as rbc
, max(case when itemid in (828) then valuenum else null end) as plt
FROM mimiciii.chartevents
where ce.itemid in (
    1127, 861, 1542 --wbc
    ,797 --Differential-Eos
    ,796 --Differential-Basos
    ,798 --Differential-Lymphs
    ,799 --Differential-Monos
    ,833 --RBC
    ,828 --Platelets
    )
AND (ce.error IS NULL OR ce.error != 1)
  group by ce.hadm_id, ce.icustay_id, ce.charttime)

,tb1 as (select b.hadm_id, b.charttime
,wbc
,eosinophils
,basophils
,lymphocytes
,monocytes
,plt)


DROP TABLE IF EXISTS blood_differential_first_time; CREATE table blood_differential_first_time
as
with tb1 as (SELECT
hadm_id
,chartdate
,wbc_max
,wbc_min
,neutrophils_abs_max
,neutrophils_abs_min
,lymphocytes_abs_max
```

```
,lymphocytes_abs_min
,monocytes_abs_max
,monocytes_abs_min
,eosinophils_abs_max
,eosinophils_abs_min
,basophils_abs_max
,basophils_abs_min
,neutrophils_max
,neutrophils_min
,lymphocytes_max
,lymphocytes_min
,monocytes_max
,monocytes_min
,basophils_max
,basophils_min
,eosinophils_max
,eosinophils_min
,rdw_max
,rdw_min
,ROW_NUMBER() OVER (PARTITION BY hadm_id ORDER BY chartdate)
FROM pivoted_blood_differential)

SELECT
hadm_id
,chartdate
,wbc_max
,wbc_min
, neutrophils_abs_max
,neutrophils_abs_min
,lymphocytes_abs_max
,lymphocytes_abs_min
,monocytes_abs_max
,monocytes_abs_min
,eosinophils_abs_max
,eosinophils_abs_min
,basophils_abs_max
,basophils_abs_min
,neutrophils_max
,neutrophils_min
,lymphocytes_max
,lymphocytes_min
,monocytes_max
,monocytes_min
```

```
,basophils_max
,basophils_min
,eosinophils_max
,rdw_max
,rdw_min
FROM tb1
WHERE row_number = 1


DROP TABLE IF EXISTS blood_differential_first_day; create table blood_differential_first_day as
with base as (SELECT
bd.hadm_id
,bd.charttime
,wbc
,neutrophils_abs
,lymphocytes_abs
,monocytes_abs
,eosinophils_abs
,basophils_abs
,neutrophils
,lymphocytes
,monocytes
,basophils
,eosinophils
,rdw
from blood_differential bd, icustay_detail icd
where bd.hadm_id = icd.hadm_id
  and bd.charttime <= DATETIME_ADD(icd.admittime, INTERVAL '1' DAY))

,tb1 as (SELECT
hadm_id
,CAST(charttime AS DATE) as chartdate
,MAX(wbc) as wbc_max
,MIN(wbc) as wbc_min
,MAX(neutrophils_abs) as neutrophils_abs_max
,MIN(neutrophils_abs) as neutrophils_abs_min
,MAX(lymphocytes_abs) as lymphocytes_abs_max
,MIN(lymphocytes_abs) as lymphocytes_abs_min
,MAX(monocytes_abs) as monocytes_abs_max
,MIN(monocytes_abs) as monocytes_abs_min
,MAX(eosinophils_abs) as eosinophils_abs_max
,MIN(eosinophils_abs) as eosinophils_abs_min
,MAX(basophils_abs) as basophils_abs_max
```

```
,MIN(basophils_abs) as basophils_abs_min
,MAX(neutrophils) as neutrophils_max
,MIN(neutrophils) as neutrophils_min
,MAX(lymphocytes) as lymphocytes_max
,MIN(lymphocytes) as lymphocytes_min
,MAX(monocytes) as monocytes_max
,MIN(monocytes) as monocytes_min
,MAX(basophils) as basophils_max
,MIN(basophils) as basophils_min
,MAX(eosinophils) as eosinophils_max
,MIN(eosinophils) as eosinophils_min
,MAX(rdw) as rdw_max
,MIN(rdw) as rdw_min
FROM base
GROUP BY hadm_id, CAST(charttime as DATE))

,tb2 as (SELECT
hadm_id
,chartdate
,wbc_max
,wbc_min
,neutrophils_abs_max
,neutrophils_abs_min
,lymphocytes_abs_max
,lymphocytes_abs_min
,monocytes_abs_max
,monocytes_abs_min
,eosinophils_abs_max
,eosinophils_abs_min
,basophils_abs_max
,basophils_abs_min
,neutrophils_max
,neutrophils_min
,lymphocytes_max
,lymphocytes_min
,monocytes_max
,monocytes_min
,basophils_max
,basophils_min
,eosinophils_max
,eosinophils_min
,rdw_max
,rdw_min
```

```
,ROW_NUMBER() OVER (PARTITION BY hadm_id ORDER BY chartdate)
FROM tb1)

SELECT
hadm_id
,chartdate
,wbc_max
,wbc_min
, neutrophils_abs_max
,neutrophils_abs_min
,lymphocytes_abs_max
,lymphocytes_abs_min
,monocytes_abs_max
,monocytes_abs_min
,eosinophils_abs_max
,eosinophils_abs_min
,basophils_abs_max
,basophils_abs_min
,neutrophils_max
,neutrophils_min
,lymphocytes_max
,lymphocytes_min
,monocytes_max
,monocytes_min
,basophils_max
,basophils_min
,eosinophils_max
,rdw_max
,rdw_min
FROM tb2
WHERE row_number = 1


create table blood_product_first_day as
-- plt first day
with tb1 as   (SELECT
icd.icustay_id
FROM icustay_detail icd, plt_transfusion pt
WHERE icd.icustay_id = pt.icustay_id
   and pt.charttime <= DATETIME_ADD(icd.admittime, INTERVAL '1' DAY))

-- rbc first day
,tb2 as (SELECT
```

```
icd.icustay_id
FROM icustay_detail icd, rbc_transfusion rt
WHERE icd.icustay_id = rt.icustay_id
   and rt.charttime <= DATETIME_ADD(icd.admittime, INTERVAL '1' DAY))

select
icustay_id
,case when icustay_id in (SELECT distinct icustay_id from tb1 ) then 1 else 0 end as
plt_transfusion
,case when icustay_id in (SELECT distinct icustay_id from tb2 ) then 1 else 0 end as
rbc_transfusion
FROM icustay_detail
group by icustay_id



-- THIS SCRIPT IS AUTOMATICALLY GENERATED. DO NOT EDIT IT DIRECTLY.
DROP TABLE IF EXISTS electrolyte; CREATE TABLE electrolyte AS

SELECT
    hadm_id
   , charttime

   -- Calcium, Total
   , MAX(CASE WHEN itemid in (50893) THEN valuenum ELSE NULL END) AS ca

   -- Phosphate
   , MAX(CASE WHEN itemid in (50970) THEN valuenum ELSE NULL END) AS phosphate

   -- Magnesium
   , MAX(CASE WHEN itemid in (50960) THEN valuenum ELSE NULL END) AS mg

   -- Ferritin
   , MAX(CASE WHEN itemid in (50924) THEN valuenum ELSE NULL END) AS ferritin

FROM mimiciii.labevents le
WHERE le.itemid IN
( 50893 -- Calcium, Total
,50924 -- Ferritin
,50960 -- Magnesium
,50970 -- Phosphate
)
AND valuenum IS NOT NULL
```

```
-- differential values cannot be negative
AND valuenum >= 0
AND hadm_id IS NOT NULL
GROUP BY   hadm_id,charttime
ORDER BY   hadm_id,charttime
;


DROP TABLE IF EXISTS electrolyte_first_day; create table electrolyte_first_day as
With tb1 as (select
ecl.hadm_id
,charttime
,ca
,phosphate
,mg
,ferritin
from electrolyte ecl,icustay_detail icd
where ecl.hadm_id = icd.hadm_id
  and ecl.charttime <= DATETIME_ADD(icd.admittime, INTERVAL '1' DAY))

,tb2 as(
    select
    hadm_id
    ,CAST(charttime AS DATE) as chartdate
,MAX(ca) as ca_max
,MIN(ca) as ca_min
,MAX(phosphate) as phosphate_max
,MIN(phosphate) as phosphate_min
,MAX(mg) as mg_max
,MIN(mg) as mg_min
,MAX(ferritin) as ferritin_max
,MIN(ferritin) as ferritin_min
FROM tb1
GROUP BY hadm_id, CAST(charttime as DATE))

,tb3 as(SELECT
hadm_id
,chartdate
,ca_max
,ca_min
,phosphate_max
,phosphate_min
,mg_max
```

```
,mg_min
,ferritin_max
,ferritin_min
,ROW_NUMBER() OVER (PARTITION BY hadm_id ORDER BY chartdate)
FROM tb2)

select
hadm_id
,ca_max
,ca_min
,phosphate_max
,phosphate_min
,mg_max
,mg_min
,ferritin_max
,ferritin_min
from tb3
where row_number = 1



CREATE table neurosurgey as
select
distinct hadm_id
from
mimiciii.procedures_icd
where icd9_code in('0101','0102','0109','0110','0111','0112','0113','0114'
,'0115','0116','0117','0118','0119','0120','0121','0122'
,'0123','0124','0125','0126','0127','0128','0129','0131'
,'0132','0139','0141','0142','0151','0152','0153','0159'
,'016' ,'0201','0202','0203','0204','0205','0206','0207'
,'0211','0212','0213','0214','022' ,'0221','0222','0231'
,'0232','0233','0234','0235','0239','0241','0242','0243'
,'0291','0292','0293','0294','0295','0296','0299')



create table normal_patients as
with tb1 as (select
distinct hadm_id
from icustay_detail icd
where first_icu_stay = 't'
EXCEPT
```

```
select
distinct hadm_id
from icustay_detail icd
where first_icu_stay = 'f'
)

select
md.subject_id
,tb1.hadm_id
,icd.icustay_id
, case when icd.gender = 'M' then 1
         else 0
         end as gender
,md.admittime
,icd.admission_age as age
,md.hospital_expire_flag as flag
,md.deathtime as dod
,icd.los_hospital
,icd.los_icu
,CASE when icd.icustay_id in (select distinct icustay_id from sepsis3) then 1
        else 0
        end as sepsis
from mimiciii.admissions md, icustay_detail icd, tb1
where tb1.hadm_id = md.hadm_id
  and tb1.hadm_id = icd.hadm_id
  and icd.admission_age >= 18   --年龄大于 18 岁
  and md.dischtime >= DATETIME_ADD(md.admittime, INTERVAL '1' DAY) --入院时间超过 1 天
  and icd.first_icu_stay = 't' --一次入院只进入过一次 ICU
order by md.subject_id, tb1.hadm_id



--DROP TABLE IF EXISTS pivoted_blood_differential; create table pivoted_blood_differential as
with base as (SELECT
hadm_id
,wbc
,neutrophils_abs
,lymphocytes_abs
,monocytes_abs
,eosinophils_abs
,basophils_abs
,neutrophils
,lymphocytes
```

```
,monocytes
,basophils
,eosinophils
,rdw
from blood_differential bd, icustay_detail icd
where bd.hadm_id = icd.hadm_id
  and bd.charttime <= DATETIME_ADD(icd.admittime, INTERVAL '1' DAY))

tb1 as (SELECT
hadm_id
,CAST(chartdate AS DATE) as chartdate
,MAX(wbc) as wbc_max
,MIN(wbc) as wbc_min
,MAX(neutrophils_abs) as neutrophils_abs_max
,MIN(neutrophils_abs) as neutrophils_abs_min
,MAX(lymphocytes_abs) as lymphocytes_abs_max
,MIN(lymphocytes_abs) as lymphocytes_abs_min
,MAX(monocytes_abs) as monocytes_abs_max
,MIN(monocytes_abs) as monocytes_abs_min
,MAX(eosinophils_abs) as eosinophils_abs_max
,MIN(eosinophils_abs) as eosinophils_abs_min
,MAX(basophils_abs) as basophils_abs_max
,MIN(basophils_abs) as basophils_abs_min
,MAX(neutrophils) as neutrophils_max
,MIN(neutrophils) as neutrophils_min
,MAX(lymphocytes) as lymphocytes_max
,MIN(lymphocytes) as lymphocytes_min
,MAX(monocytes) as monocytes_max
,MIN(monocytes) as monocytes_min
,MAX(basophils) as basophils_max
,MIN(basophils) as basophils_min
,MAX(eosinophils) as eosinophils_max
,MIN(eosinophils) as eosinophils_min
,MAX(rdw) as rdw_max
,MIN(rdw) as rdw_min
FROM base
GROUP BY subject_id, hadm_id, CAST(charttime as DATE))

tb2 as (SELECT
hadm_id
,chartdate
,wbc_max
,wbc_min
```

```
,neutrophils_abs_max
,neutrophils_abs_min
,lymphocytes_abs_max
,lymphocytes_abs_min
,monocytes_abs_max
,monocytes_abs_min
,eosinophils_abs_max
,eosinophils_abs_min
,basophils_abs_max
,basophils_abs_min
,neutrophils_max
,neutrophils_min
,lymphocytes_max
,lymphocytes_min
,monocytes_max
,monocytes_min
,basophils_max
,basophils_min
,eosinophils_max
,eosinophils_min
,rdw_max
,rdw_min
,ROW_NUMBER() OVER (PARTITION BY hadm_id ORDER BY chartdate)
FROM pivoted_blood_differential)

SELECT
hadm_id
,chartdate
,wbc_max
,wbc_min
, neutrophils_abs_max
,neutrophils_abs_min
,lymphocytes_abs_max
,lymphocytes_abs_min
,monocytes_abs_max
,monocytes_abs_min
,eosinophils_abs_max
,eosinophils_abs_min
,basophils_abs_max
,basophils_abs_min
,neutrophils_max
,neutrophils_min
,lymphocytes_max
```

```
,lymphocytes_min
,monocytes_max
,monocytes_min
,basophils_max
,basophils_min
,eosinophils_max
,rdw_max
,rdw_min
FROM tb2
WHERE row_number = 1


-- Retrieves instances of red blood cell transfusions
CREATE TABLE plt_transfusion AS
with raw_plt as (
  SELECT
      CASE
        WHEN amount IS NOT NULL THEN amount
        WHEN stopped IS NOT NULL THEN 0
        -- impute 375 mL when unit is not documented
        ELSE 200
      END AS amount
    , amountuom
    , icustay_id
    , charttime
  FROM mimiciii.inputevents_cv
  WHERE itemid = 30006   -- Platelets
  AND icustay_id IS NOT NULL
  UNION ALL
  SELECT amount
    , amountuom
    , icustay_id
    , endtime AS charttime
  FROM mimiciii.inputevents_mv
  WHERE itemid =   225170     -- Platelets
  AND amount > 0
  AND icustay_id IS NOT NULL
),
pre_icu_plt as (
  SELECT
    sum(amount) as amount, icustay_id
  FROM mimiciii.inputevents_mv
  WHERE itemid IN (
```

```
    227071   -- PACU Platelet Intake
  )
  AND amount > 0
  AND icustay_id IS NOT NULL
  GROUP BY icustay_id
),
cumulative AS (
  SELECT
    sum(amount) over (PARTITION BY icustay_id ORDER BY charttime DESC) AS amount
    , amountuom
    , icustay_id
    , charttime
    , DATETIME_DIFF(lag(charttime) over (PARTITION BY icustay_id ORDER BY charttime ASC),
charttime, 'HOUR') AS delta
  FROM raw_plt
)
-- We consider any transfusions started within 1 hr of the last one
-- to be part of the same event
SELECT
    cm.icustay_id
  , cm.charttime
  , ROUND(CAST(cm.amount AS numeric) - CASE
      WHEN ROW_NUMBER() OVER w = 1 THEN CAST(0 AS numeric)
      ELSE CAST(lag(cm.amount) OVER w AS numeric)
    END, 2) AS amount
  , ROUND(CAST(cm.amount AS numeric) + CASE
      WHEN CAST(pre.amount AS numeric) IS NULL THEN CAST(0 AS numeric)
      ELSE CAST(pre.amount AS numeric)
    END, 2) AS totalamount
  , cm.amountuom
FROM cumulative AS cm
LEFT JOIN pre_icu_plt AS pre
  USING (icustay_id)
WHERE delta IS NULL OR delta < -1
WINDOW w AS (PARTITION BY cm.icustay_id ORDER BY cm.charttime DESC)
ORDER BY icustay_id, charttime;

--所有创伤性脑病病人
with tb1 as (select
distinct hadm_id
from
mimiciii.diagnoses_icd
WHERE icd9_code IN (
```

'3102' --Other Personality and Behavioral Disorders due to Known Physiological Condition
,'8500','8502','8503','8504','8505','8509' --Concussion
,'85011','85012'
,'85100','85101','85102','85103','85104','85105','85106','85109' --Cerebral Laceration and Contusion Without Mention of Open Intracranial Wound
,'85120','85121','85122','85123','85124','85125','85126','85129'
,'85140','85141','85142','85143','85144','85145','85146','85149'
,'85160','85161','85162','85163','85164','85165','85166','85169'
,'85180','85181','85182','85183','85184','85185','85186','85189'
,'85200','85201','85202','85203','85204','85205','85206','85209' --Subarachnoid Subdural and Extradural Hemorrhage Following Injury Without Mention of Open Intracranial Wound
,'85220','85221','85222','85223','85224','85225','85226','85229'
,'85240','85241','85242','85243','85244','85245','85246','85249'
,'85300','85301','85302','85303','85304','85305','85306','85309' --Other and Unspecified Intracranial Hemorrhage Following Injury
,'85400','85401','85402','85403','85404','85405','85406','85409'
,'85110','85111','85112','85113','85114','85115','85116','85119' --Cerebral Laceration and Contusion With Open Intracranial Wound
,'85130','85131','85132','85133','85134','85135','85136','85139'
,'85150','85151','85152','85153','85154','85155','85156','85159'
,'85170','85171','85172','85173','85174','85175','85176','85179'
,'85190','85191','85192','85193','85194','85195','85196','85199'
,'85210','85211','85212','85213','85214','85215','85216','85219' --Subarachnoid Subdural and Extradural Hemorrhage Following Injury With Open Intracranial Wound
,'85230','85231','85232','85233','85234','85235','85236','85239'
,'85250','85251','85252','85253','85254','85255','85256','85259'
,'85310','85311','85312','85313','85314','85315','85316','85319' --Other and Unspecified Intracranial Hemorrhage With Open Intracranial Wound
,'85410','85411','85412','85413','85414','85415','85416','85419' --Intracranial Injury of Other and Unspecified Nature With Open Intracranial Wound
,'80000','80001','80002','80003','80004','80005','80006','80009' --Fracture of Vault of Skull
,'80010','80011','80012','80013','80014','80015','80016','80019'
,'80020','80021','80022','80023','80024','80025','80026','80029'
,'80030','80031','80032','80033','80034','80035','80036','80039'
,'80040','80041','80042','80043','80044','80045','80046','80049'
,'80050','80051','80052','80053','80054','80055','80056','80059'
,'80060','80061','80062','80063','80064','80065','80066','80069'
,'80070','80071','80072','80073','80074','80075','80076','80079'
,'80080','80081','80082','80083','80084','80085','80086','80089'
,'80090','80091','80092','80093','80094','80095','80096','80099'
,'80100','80101','80102','80103','80104','80105','80106','80109' --Fracture of Base of Skull
,'80110','80111','80112','80113','80114','80115','80116','80119'
,'80120','80121','80122','80123','80124','80125','80126','80129'

,'80130','80131','80132','80133','80134','80135','80136','80139'
,'80140','80141','80142','80143','80144','80145','80146','80149'
,'80150','80151','80152','80153','80154','80155','80156','80159'
,'80160','80161','80162','80163','80164','80165','80166','80169'
,'80170','80171','80172','80173','80174','80175','80176','80179'
,'80180','80181','80182','80183','80184','80185','80186','80189'
,'80190','80191','80192','80193','80194','80195','80196','80199'
,'80300','80301','80302','80303','80304','80305','80306','80309' --Other and Unqualified Skull Fractures
,'80310','80311','80312','80313','80314','80315','80316','80319'
,'80320','80321','80322','80323','80324','80325','80326','80329'
,'80330','80331','80332','80333','80334','80335','80336','80339'
,'80340','80341','80342','80343','80344','80345','80346','80349'
,'80350','80351','80352','80353','80354','80355','80356','80359'
,'80360','80361','80362','80363','80364','80365','80366','80369'
,'80370','80371','80372','80373','80374','80375','80376','80379'
,'80380','80381','80382','80383','80384','80385','80386','80389'
,'80390','80391','80392','80393','80394','80395','80396','80399'
,'80400','80401','80402','80403','80404','80405','80406','80409' --Multiple Fractures Involving Skull or Face With Other Bones
,'80410','80411','80412','80413','80414','80415','80416','80419'
,'80420','80421','80422','80423','80424','80425','80426','80429'
,'80430','80431','80432','80433','80434','80435','80436','80439'
,'80440','80441','80442','80443','80444','80445','80446','80449'
,'80450','80451','80452','80453','80454','80455','80456','80459'
,'80460','80461','80462','80463','80464','80465','80466','80469'
,'80470','80471','80472','80473','80474','80475','80476','80479'
,'80480','80481','80482','80483','80484','80485','80486','80489'
,'80490','80491','80492','80493','80494','80495','80496','80499'
,'9501','9502','9503' --Injury to Optic chiasm and Optic Tract
,'9070','95901' --other
))

,tb2 as (select
DISTINCT tb1.hadm_id
,np.icustay_id
,gender
,admittime
,age
,flag
,dod
,los_hospital
,los_icu

```
,sepsis
from tb1 ,normal_patients np
WHERE np.hadm_id = tb1.hadm_id)

SELECT
tb2.hadm_id
,tb2.icustay_id
,gender
,age
,lfd.bun_max
,aft.alb
,vfd.heartrate_max as hr_max
,vfd.meanbp_min as mbp_min
,vfd.resprate_max as rr_max
,vfd.tempc_max t_max
,vfd.spo2_min
,vfd.glucose_max as glu_max
,lfd.bicarbonate_min as sb_min
,lfd.hematocrit_min as hct_min
,lfd.hemoglobin_min as hb_min
,lfd.platelet_min as plt_min
,lfd.wbc_max
,lfd.aniongap_max as ag_max
,lfd.creatinine_max cr_max
,efd.ca_min
,lfd.chloride_max as cl_max
,lfd.sodium_max as na_max
,lfd.potassium_min as k_min
,lfd.inr_max
,lfd.pt_max
,lfd.ptt_max
,efd.phosphate_max as phosphate
,efd.mg_max as mg
,bdfd.rdw_max as rdw
,sofa.sofa
,apsiii.apsiii
,charlson.congestive_heart_failure
,charlson.chronic_pulmonary_disease
,charlson.rheumatic_disease
,charlson.renal_disease
,case when charlson.diabetes_without_cc = 0 and charlson.diabetes_with_cc = 0
        then 0
        else 1
```

```
        end as diabetes
,case when charlson.mild_liver_disease = 0 and severe_liver_disease = 0
        then 0
        else 1
        end as liver_disease
,charlson.charlson_comorbidity_index as charlson
,gfd.mingcs as gcs_min
,case when tb2.hadm_id in (select distinct hadm_id FROM mimiciii.diagnoses_icd
WHERE icd9_code in ('85300','85301','85302','85303','85304','85305','85306','85309' --Other and Unspecified Intracranial Hemorrhage Following Injury
,'85400','85401','85402','85403','85404','85405','85406','85409')) then 1
else 0 end as intraparenchymal_heorrhage

,case when tb2.hadm_id in (select distinct hadm_id FROM mimiciii.diagnoses_icd
WHERE icd9_code in ('85240','85241','85242','85243','85244','85245','85246','85249' --extradural hemorrhage following injury w/o mention of open intracrania wound
,'85250','85251','85252','85253','85254','85255','85256','85259' --extradural hemorrhage following injury with open intracranial wound...
)) then 1
else 0 end as extradural_hemorrhage

,case when tb2.hadm_id in (select distinct hadm_id FROM mimiciii.diagnoses_icd
WHERE icd9_code in ('85220','85221','85222','85223','85224','85225','85226','85229' --subdural hemorrhage following injury w/o mention of open intracranial wound
,'85230','85231','85232','85233','85234','85235','85236','85239' --subdural hemorrhage following injury with open intracranial wound...
)) then 1
else 0 end as subdural_hemorrhage

,case when tb2.hadm_id in (select distinct hadm_id FROM mimiciii.diagnoses_icd
WHERE icd9_code in ('85200','85201','85202','85203','85204','85205','85206','85209'
--subarchnoid hemorrhage following injury w/o mention of open intracranial wound
,'85210','85211','85212','85213','85214','85215','85216','85219' --subarachnoid hemorrhage following injury with open intracranial wound...
)) then 1
else 0 end as subarachnoi_hemorrhage
,bpfd.rbc_transfusion as firstday_rbc
,bpfd.plt_transfusion as firstday_plt
,CASE when tb2.hadm_id in (SELECT distinct hadm_id from neurosurgey ) then 1
        else 0 end as neurosurgery
,sepsis
,flag
,los_icu
```

```
,los_hospital
,admittime
,dod
,CASE when dod is null then 1
	else case when datetime_diff(dod, CAST(admittime AS DATE), 'DAY')/28 < 1 then
datetime_diff(dod, CAST(admittime AS DATE), 'DAY')/28
			when datetime_diff(dod, CAST(admittime AS DATE), 'DAY')/28 >= 1 then 1
			else null end
	end as onemouthsurvival

,CASE when dod is null then 0
	else case when datetime_diff(dod, CAST(admittime AS DATE), 'DAY')/28 < 1 then 1
			when datetime_diff(dod, CAST(admittime AS DATE), 'DAY')/28 >= 1 then 0
			else null end
	end as onemouthmortality

,CASE when dod is null then 3
	else case when datetime_diff(dod, CAST(admittime AS DATE), 'DAY')/28 < 3 then
datetime_diff(dod, CAST(admittime AS DATE), 'DAY')/28
			when datetime_diff(dod, CAST(admittime AS DATE), 'DAY')/28 >= 3 then 3
			else null end
	end as threemouthsurvival

,CASE when dod is null then 0
	else case when datetime_diff(dod, CAST(admittime AS DATE), 'DAY')/28 < 3 1
			when datetime_diff(dod, CAST(admittime AS DATE), 'DAY')/28 >= 3 then 0
			else null end
	end as threemouthmortality

,CASE when dod is null then 12
	else case when datetime_diff(dod, CAST(admittime AS DATE), 'DAY')/28 < 12 then
datetime_diff(dod, CAST(admittime AS DATE), 'DAY')/28
			when datetime_diff(dod, CAST(admittime AS DATE), 'DAY')/28 >= 12 then 12
			else null end
	end as oneyearsurvival

,CASE when dod is null then 0
	else case when datetime_diff(dod, CAST(admittime AS DATE), 'DAY')/28 < 12 then 1
			when datetime_diff(dod, CAST(admittime AS DATE), 'DAY')/28 >= 12 then 0
			else null end
	end as oneyearmortality

,bfd.ph_min
```

```
,bfd.po2_min
,bfd.pco2_max
,bfd.aado2_calc_max
,bfd.pao2fio2_min
,bfd.lactate_max
,lfd.bilirubin_max
,bdfd.neutrophils_abs_max
,bdfd.lymphocytes_abs_min
,case when bdfd.lymphocytes_abs_min is null or bdfd.lymphocytes_abs_min = 0 then null
        else lfd.platelet_min * bdfd.neutrophils_abs_max/bdfd.lymphocytes_abs_min
                        end as sii
,efd.ferritin_max
FROM tb2
left join vitals_first_day vfd on tb2.icustay_id = vfd.icustay_id
left join bg_first_day_art bfd on tb2.icustay_id = bfd.icustay_id
left join labs_first_day lfd     on tb2.hadm_id = lfd.hadm_id
left join charlson                 on tb2.hadm_id = charlson.hadm_id
left join apsiii                   on tb2.hadm_id = apsiii.hadm_id
left join sofa                     on tb2.icustay_id = sofa.icustay_id
left join gcs_first_day   gfd      on tb2.hadm_id = gfd.hadm_id
left join blood_differential_first_day bdfd on tb2.hadm_id = bdfd.hadm_id
left join blood_product_first_day bpfd on tb2.icustay_id = bpfd.icustay_id
left join electrolyte_first_day efd on tb2.hadm_id = efd.hadm_id
left join alb_first_time aft on tb2.hadm_id = aft.hadm_id


-- THIS SCRIPT IS AUTOMATICALLY GENERATED. DO NOT EDIT IT DIRECTLY.
 CREATE TABLE albumin AS
-- extract chemistry labs
-- excludes point of care tests (very rare)
-- blood gas measurements are *not* included in this query
-- instead they are in bg.sql
SELECT
      MAX(subject_id) AS subject_id
   , MAX(hadm_id) AS hadm_id
   , MAX(charttime) AS charttime
   , le.specimen_id
   -- convert from itemid into a meaningful column
   , MAX(CASE WHEN itemid = 50862 AND valuenum <=      10 THEN valuenum ELSE NULL END) AS
albumin
FROM mimiciv_hosp.labevents le
WHERE le.itemid =   50862 -- ALBUMIN | CHEMISTRY | BLOOD | 146697
AND valuenum IS NOT NULL
```

```
-- lab values cannot be 0 and cannot be negative
AND hadm_id is not null
GROUP BY le.specimen_id
;

with tb1 as (select
stay_id as icustay_id
,weight
,ROW_NUMBER()over(partition by stay_id order by starttime)
FROM mimiciv_derived.weight_durations)

,weight as(select
icustay_id
,weight
from tb1
where row_number = 1 )

SELECT
distinct icustay_id
,w.weight
,h.height
,w.weight/((h.height/100)^2) as bmi
from weight w, mimiciv_derived.height h
where w.icustay_id = h.stay_id
order by icustay_id

create table first_day_blood_products as
with tb1 as (SELECT
hadm_id
,stay_id as icustay_id
,starttime
,amount
,itemid
FROM mimiciv_icu.inputevents
where itemid in (
225168,226368,227070,225170,226369,227071)
)

,tb2 as (SELECT
hadm_id
,icustay_id
,starttime
,amount
```

```
,CASE when itemid in (225168,226368,227070) then 'rbc'
        else 'plt'
        end as blood_products
from tb1 )

SELECT
tb2.hadm_id
,icustay_id
,blood_products
,amount
from tb2,mimiciv_hosp.admissions adm
where tb2.hadm_id = adm.hadm_id
   and tb2.starttime <= DATETIME_ADD(adm.admittime, INTERVAL '1' DAY)

create table first_day_gcs_min as
with tb1 as (select
stay_id
, CAST(charttime AS DATE) as chartdate
,gcs
FROM gcs)

,tb2 as (
select
stay_id
,chartdate
,MIN(gcs) as gcs_min
from tb1
group by
stay_id
,chartdate
ORDER BY
stay_id
,chartdate)

,tb3 as (SELECT
stay_id
,chartdate
,gcs_min
,ROW_NUMBER()OVER(PARTITION BY stay_id ORDER BY chartdate)
FROM tb2
order by
stay_id
,chartdate)
```

```
SELECT
stay_id
,chartdate
,gcs_min
from tb3
where row_number = 1
create table first_day_rdw as
with tb1 as (select
hadm_id
,charttime
,rdw
from complete_blood_count
where hadm_id is not null)

,tb2 as (select
hadm_id
,rdw
,ROW_NUMBER()OVER(PARTITION BY hadm_id ORDER BY charttime)
from tb1)

select
hadm_id
,rdw
from tb2
where row_number = 1




create table first_time_alb as
WITH tb1 AS (SELECT
hadm_id
,CAST(charttime as DATE) as chartdate
,albumin
from mimiciv_derived.albumin
order by hadm_id, chartdate
)

,tb2 as (
    SELECT
    hadm_id
    ,chartdate
```

```
    ,albumin
    ,ROW_NUMBER () OVER (PARTITION BY hadm_id ORDER BY chartdate)
    FROM tb1
    order by hadm_id,chartdate
)

SELECT
hadm_id
,albumin
FROM tb2
WHERE row_number = 1
ORDER BY hadm_id

CREATE TABLE first_time_bg_art as
with tb1 as (SELECT
hadm_id
,CAST(charttime as DATE) AS chartdate
,ph
,po2
,pco2
,aado2_calc
,pao2fio2ratio
,lactate
FROM mimiciv_derived.bg
WHERE specimen = 'ART.')

,tb2 as (select
hadm_id
,chartdate
,MIN(ph) as ph_min
,MIN(po2) AS po2_min
,MAX(pco2) AS pco2_max
,MAX(aado2_calc) AS aado2_calc_max
,MIN(pao2fio2ratio) AS pf_ratio_min
,MAX(lactate) AS lactate_max
FROM tb1
GROUP BY hadm_id,chartdate
ORDER BY hadm_id, chartdate)

,tb3 AS (
    SELECT
    hadm_id
    ,chartdate
```

```
    ,ph_min
    ,po2_min
    ,pco2_max
    ,aado2_calc_max
    ,pf_ratio_min
    ,lactate_max
    ,ROW_NUMBER() OVER (PARTITION BY hadm_id ORDER BY chartdate)
    FROM tb2
)
SELECT
hadm_id
    ,chartdate
    ,ph_min
    ,po2_min
    ,pco2_max
    ,aado2_calc_max
    ,pf_ratio_min
    ,lactate_max
FROM tb3
WHERE row_number = 1


with tb1 as (
    SELECT
    hadm_id
    ,CAST(charttime AS DATE) AS chartdate
    ,alt
    ,ast
    ,alp
    ,bilirubin_total
    ,ld_ldh
    FROM mimiciv_derived.enzyme
    WHERE hadm_id is not null
)

,tb2 as (
    SELECT
    hadm_id
    ,chartdate
    ,MAX(alt) AS alt_max
    ,MAX(ast) AS ast_max
    ,MAX(alp) AS alp_max
    ,MAX(bilirubin_total) AS bili_max
```

```
    ,MAX(ld_ldh) AS ldh_max
    FROM tb1
    GROUP BY   hadm_id, chartdate
)

,tb3 AS (
    SELECT
    hadm_id
    ,chartdate
    ,alt_max
    ,ast_max
    ,alp_max
    ,bili_max
    ,ldh_max
    ,ROW_NUMBER() OVER (PARTITION BY hadm_id ORDER BY chartdate)
    FROM tb2
)

SELECT
 hadm_id
    ,chartdate
    ,alt_max
    ,ast_max
    ,alp_max
    ,bili_max
    ,ldh_max
    FROM tb3
        WHERE row_number = 1

create table first_time_mg as
with tb1 as (SELECT
hadm_id
,charttime
,mg
,ROW_NUMBER()OVER(PARTITION BY hadm_id ORDER BY charttime)
from mg)

SELECT
hadm_id
,mg
from tb1
where row_number = 1
```

```
create table first_time_phosphate as
with tb1 as (SELECT
hadm_id
,charttime
,phosphate
,ROW_NUMBER()OVER(PARTITION BY hadm_id ORDER BY charttime)
from phosphate)

SELECT
hadm_id
,phosphate
from tb1
where row_number = 1

CREATE TABLE mg AS
SELECT
      MAX(subject_id) AS subject_id
   , MAX(hadm_id) AS hadm_id
   , MAX(charttime) AS charttime
   , le.specimen_id
   -- convert from itemid into a meaningful column
   , MAX(CASE WHEN itemid = 50960 THEN valuenum ELSE NULL END) AS mg
FROM mimiciv_hosp.labevents le
WHERE le.itemid = 50960
AND valuenum IS NOT NULL
-- lab values cannot be 0 and cannot be negative
AND valuenum > 0
---AND valuenum <> '__'
AND hadm_id IS NOT NULL
GROUP BY le.specimen_id
;
neuro_produces

icd10
'00160KB','00160ZB','00163KB','00163ZB','00164KB','00164ZB','00500ZZ','00503ZZ'
,'00504ZZ','00510ZZ','00513ZZ','00514ZZ','00520ZZ','00523ZZ','00524ZZ','00560ZZ'
,'00563ZZ','00564ZZ','00570ZZ','00573ZZ','00574ZZ','00580ZZ','00583ZZ','00584ZZ'
,'00590ZZ','00593ZZ','00594ZZ','005A0ZZ','005A3ZZ','005A4ZZ','005B0ZZ','005B3ZZ'
,'005B4ZZ','005C0ZZ','005C3ZZ','005C4ZZ','005D0ZZ','005D3ZZ','005D4ZZ','00760ZZ'
,'00763ZZ','00764ZZ','00800ZZ','00803ZZ','00804ZZ','00870ZZ','00873ZZ','00874ZZ'
,'00880ZZ','00883ZZ','00884ZZ','009000Z','00900ZZ','009030Z','00903ZZ','009040Z'
,'00904ZZ','009100Z','00910ZZ','009130Z','00913ZZ','009140Z','00914ZZ','009200Z'
```

,'00920ZZ','009230Z','00923ZZ','009240Z','00924ZZ','009300Z','00930ZZ','009330Z'
,'00933ZZ','009340Z','00934ZZ','009400Z','00940ZZ','009430Z','00943ZZ','009440Z'
,'00944ZZ','009500Z','00950ZZ','009530Z','00953ZZ','009540Z','00954ZZ','009600Z'
,'00960ZZ','009630Z','00963ZZ','009640Z','00964ZZ','009700Z','00970ZZ','009730Z'
,'00973ZZ','009740Z','00974ZZ','009800Z','00980ZZ','009830Z','00983ZZ','009840Z'
,'00984ZZ','009900Z','00990ZZ','009930Z','00993ZZ','009940Z','00994ZZ','009A00Z'
,'009A0ZZ','009A30Z','009A3ZZ','009A40Z','009A4ZZ','009B00Z','009B0ZZ','009B30Z'
,'009B3ZZ','009B40Z','009B4ZZ','009C00Z','009C0ZZ','009C30Z','009C3ZZ','009C40Z'
,'009C4ZZ','009D00Z','009D0ZZ','009D30Z','009D3ZZ','009D40Z','009D4ZZ','00B00ZZ'
,'00B03ZZ','00B04ZZ','00B10ZZ','00B13ZZ','00B14ZZ','00B20ZZ','00B23ZZ','00B24ZZ'
,'00B60ZZ','00B63ZZ','00B64ZZ','00B70ZZ','00B73ZZ','00B74ZZ','00B80ZZ','00B83ZZ'
,'00B84ZZ','00B90ZZ','00B93ZZ','00B94ZZ','00BA0ZZ','00BA3ZZ','00BA4ZZ','00BB0ZZ'
,'00BB3ZZ','00BB4ZZ','00BC0ZZ','00BC3ZZ','00BC4ZZ','00BD0ZZ','00BD3ZZ','00C00ZZ'
,'00C03ZZ','00C04ZZ','00C10ZZ','00C13ZZ','00C14ZZ','00C20ZZ','00C23ZZ','00C24ZZ'
,'00C30ZZ','00C33ZZ','00C34ZZ','00C40ZZ','00C43ZZ','00C44ZZ','00C50ZZ','00C53ZZ'
,'00C54ZZ','00C60ZZ','00C63ZZ','00C64ZZ','00C70ZZ','00C73ZZ','00C74ZZ','00C80ZZ'
,'00C83ZZ','00C84ZZ','00C90ZZ','00C93ZZ','00C94ZZ','00CA0ZZ','00CA3ZZ','00CA4ZZ'
,'00CB0ZZ','00CB3ZZ','00CB4ZZ','00CC0ZZ','00CC3ZZ','00CC4ZZ','00CD0ZZ','00CD3ZZ'
,'00CD4ZZ','00D00ZZ','00D03ZZ','00D04ZZ','00D10ZZ','00D13ZZ','00D14ZZ','00D20ZZ'
,'00D23ZZ','00D24ZZ','00D70ZZ','00D73ZZ','00D74ZZ','00F30ZZ','00F33ZZ','00F34ZZ'
,'00F40ZZ','00F43ZZ','00F44ZZ','00F50ZZ','00F53ZZ','00F54ZZ','00F60ZZ','00F63ZZ'
,'00F64ZZ','00H002Z','00H003Z','00H004Z','00H00MZ','00H00YZ','00H032Z','00H033Z'
,'00H03MZ','00H03YZ','00H042Z','00H043Z','00H04MZ','00H04YZ','00H602Z','00H603Z'
,'00H60MZ','00H60YZ','00H632Z','00H633Z','00H63MZ','00H63YZ','00H642Z','00H643Z'
,'00H64MZ','00H64YZ','00J00ZZ','00J03ZZ','00J04ZZ','00K00ZZ','00K03ZZ','00K04ZZ'
,'00K70ZZ','00K73ZZ','00K74ZZ','00K80ZZ','00K83ZZ','00K84ZZ','00K90ZZ','00K93ZZ'
,'00K94ZZ','00KA0ZZ','00KA3ZZ','00KA4ZZ','00KB0ZZ','00KB3ZZ','00KB4ZZ','00KC0ZZ'
,'00KC3ZZ','00KC4ZZ','00KD0ZZ','00KD3ZZ','00KD4ZZ','00N00ZZ','00N03ZZ','00N04ZZ'
,'00N10ZZ','00N13ZZ','00N14ZZ','00N20ZZ','00N23ZZ','00N24ZZ','00N60ZZ','00N63ZZ'
,'00N64ZZ','00N70ZZ','00N73ZZ','00N74ZZ','00N80ZZ','00N83ZZ','00N84ZZ','00N90ZZ'
,'00N93ZZ','00N94ZZ','00NA0ZZ','00NA3ZZ','00NA4ZZ','00NB0ZZ','00NB3ZZ','00NB4ZZ'
,'00NC0ZZ','00NC3ZZ','00NC4ZZ','00ND0ZZ','00ND3ZZ','00ND4ZZ','00P000Z','00P002Z'
,'00P003Z','00P007Z','00P00JZ','00P00KZ','00P00MZ','00P00YZ','00P030Z','00P032Z'
,'00P033Z','00P037Z','00P03JZ','00P03KZ','00P03MZ','00P03YZ','00P040Z','00P042Z'
,'00P043Z','00P047Z','00P04JZ','00P04KZ','00P04MZ','00P04YZ','00P600Z','00P602Z'
,'00P603Z','00P60MZ','00P60YZ','00P630Z','00P632Z','00P633Z','00P63MZ','00P63YZ'
,'00P640Z','00P642Z','00P643Z','00P64MZ','00P64YZ','00Q00ZZ','00Q03ZZ','00Q04ZZ'
,'00Q10ZZ','00Q13ZZ','00Q14ZZ','00Q20ZZ','00Q23ZZ','00Q24ZZ','00Q60ZZ','00Q63ZZ'
,'00Q64ZZ','00Q70ZZ','00Q73ZZ','00Q74ZZ','00Q80ZZ','00Q83ZZ','00Q84ZZ','00Q90ZZ'
,'00Q93ZZ','00Q94ZZ','00QA0ZZ','00QA3ZZ','00QA4ZZ','00QB0ZZ','00QB3ZZ','00QB4ZZ'
,'00QC0ZZ','00QC3ZZ','00QC4ZZ','00QD0ZZ','00QD3ZZ','00QD4ZZ','00R107Z','00R10JZ'
,'00R10KZ','00R147Z','00R14JZ','00R14KZ','00R207Z','00R20JZ','00R20KZ','00R247Z'
,'00R24JZ','00R24KZ','00R607Z','00R60JZ','00R60KZ','00R647Z','00R64JZ','00R64KZ'

,'00T70ZZ','00T73ZZ','00T74ZZ','00U107Z','00U10JZ','00U10KZ','00U137Z','00U13JZ'
,'00U13KZ','00U147Z','00U14JZ','00U14KZ','00U207Z','00U20JZ','00U20KZ','00U237Z'
,'00U23JZ','00U23KZ','00U247Z','00U24JZ','00U24KZ','00U607Z','00U60JZ','00U60KZ'
,'00U637Z','00U63JZ','00U63KZ','00U647Z','00U64JZ','00U64KZ','00W000Z','00W002Z'
,'00W003Z','00W007Z','00W00JZ','00W00KZ','00W00MZ','00W00YZ','00W030Z','00W032Z'
,'00W033Z','00W037Z','00W03JZ','00W03KZ','00W03MZ','00W03YZ','00W040Z','00W042Z'
,'00W043Z','00W047Z','00W04JZ','00W04KZ','00W04MZ','00W04YZ','00W600Z','00W602Z'
,'00W603Z','00W60MZ','00W60YZ','00W630Z','00W632Z','00W633Z','00W63MZ','00W63YZ'
,'00W640Z','00W642Z','00W643Z','00W64MZ','00W64YZ','031G09G','031G0AG','031G0JG'
,'031G0KG','031G0ZG','031S09G','031S0AG','031S0JG','031S0KG','031S0ZG','031T09G'
,'031T0AG','031T0JG','031T0KG','031T0ZG','035G0ZZ','035G3ZZ','035G4ZZ','037G04Z'
,'037G05Z','037G06Z','037G07Z','037G0DZ','037G0EZ','037G0FZ','037G0GZ','037G0ZZ'
,'037G34Z','037G35Z','037G36Z','037G37Z','037G3DZ','037G3EZ','037G3FZ','037G3GZ'
,'037G3ZZ','037G44Z','037G45Z','037G46Z','037G47Z','037G4DZ','037G4EZ','037G4FZ'
,'037G4GZ','037G4ZZ','039G00Z','039G0ZZ','039G30Z','039G3ZZ','039G40Z','039G4ZZ'
,'03BG0ZZ','03BG3ZZ','03BG4ZZ','03CG0ZZ','03CG3Z7','03CG3ZZ','03CG4ZZ','03LG0BZ'
,'03LG0CZ','03LG0DZ','03LG0ZZ','03LG3CZ','03LG3ZZ','03LG4BZ','03LG4CZ','03LG4DZ'
,'03LG4ZZ','03RG07Z','03RG0JZ','03RG0KZ','03RG47Z','03RG4JZ','03RG4KZ','03UG07Z'
,'03UG0JZ','03UG0KZ','03UG37Z','03UG3JZ','03UG3KZ','03UG47Z','03UG4JZ','03UG4KZ'
,'03VG0BZ','03VG0CZ','03VG0DZ','03VG0HZ','03VG0ZZ','03VG3BZ','03VG3CZ','03VG3DZ'
,'03VG3HZ','03VG3ZZ','03VG4BZ','03VG4CZ','03VG4DZ','03VG4HZ','03VG4ZZ','055L0ZZ'
,'055L3ZZ','055L4ZZ','05BL0ZZ','05BL3ZZ','05BL4ZZ','05LL0CZ','05LL0DZ','05LL0ZZ'
,'05LL3CZ','05LL3ZZ','05LL4CZ','05LL4DZ','05LL4ZZ','05RL07Z','05RL0JZ','05RL0KZ'
,'05RL47Z','05RL4JZ','05RL4KZ','0G500ZZ','0G503ZZ','0G504ZZ','0G510ZZ','0G513ZZ'
,'0G514ZZ','0G800ZZ','0G803ZZ','0G804ZZ','0G9000Z','0G900ZZ','0G9030Z','0G903ZZ'
,'0G9040Z','0G904ZZ','0G9100Z','0G910ZZ','0G9130Z','0G913ZZ','0G9140Z','0G914ZZ'
,'0GB00ZZ','0GB03ZZ','0GB04ZZ','0GB10ZZ','0GB13ZZ','0GB14ZZ','0GC00ZZ','0GC03ZZ'
,'0GC04ZZ','0GC10ZZ','0GC13ZZ','0GC14ZZ','0GJ00ZZ','0GJ03ZZ','0GJ04ZZ','0GJ10ZZ'
,'0GJ13ZZ','0GJ14ZZ','0GN00ZZ','0GN03ZZ','0GN04ZZ','0GN10ZZ','0GN13ZZ','0GN14ZZ'
,'0GP000Z','0GP030Z','0GP040Z','0GP100Z','0GP130Z','0GP140Z','0GQ00ZZ','0GQ03ZZ'
,'0GQ04ZZ','0GQ10ZZ','0GQ13ZZ','0GQ14ZZ','0GT00ZZ','0GT04ZZ','0GT10ZZ','0GT14ZZ'
,'0GW000Z','0GW030Z','0GW040Z','0GW100Z','0GW130Z','0GW140Z','0W110J9','0W110JB'
,'0W110JG','0W110JJ','0W9100Z','0W910ZZ','0W9130Z','0W913ZZ','0W9140Z','0W914ZZ'
,'0WC10ZZ','0WC13ZZ','0WC14ZZ','0WF10ZZ','0WF13ZZ','0WF14ZZ','0WH101Z','0WH103Z'
,'0WH10YZ','0WH131Z','0WH133Z','0WH13YZ','0WH141Z','0WH143Z','0WH14YZ','0WJ10ZZ'
,'0WJ13ZZ','0WJ14ZZ','0WP100Z','0WP101Z','0WP103Z','0WP10JZ','0WP10YZ','0WP130Z'
,'0WP131Z','0WP133Z','0WP13JZ','0WP13YZ','0WP140Z','0WP141Z','0WP143Z','0WP14JZ'
,'0WP14YZ','0WW100Z','0WW101Z','0WW103Z','0WW10JZ','0WW10YZ','0WW130Z','0WW131Z'
,'0WW133Z','0WW13JZ','0WW13YZ','0WW140Z','0WW141Z','0WW143Z','0WW14JZ','0WW14YZ'
,'3E0Q004','3E0Q005','3E0Q00M','3E0Q028','3E0Q029','3E0Q03Z','3E0Q06Z','3E0Q07Z'
,'3E0Q0AZ','3E0Q0BZ','3E0Q0E0','3E0Q0E1','3E0Q0GC','3E0Q0HZ','3E0Q0KZ','3E0Q0NZ'
,'3E0Q0SF','3E0Q0TZ'

```
'0101','0102','0109','0110','0111','0112','0113','0114'
,'0115','0116','0117','0118','0119','0120','0121','0122'
,'0123','0124','0125','0126','0127','0128','0129','0131'
,'0132','0139','0141','0142','0151','0152','0153','0159'
,'016' ,'0201','0202','0203','0204','0205','0206','0207'
,'0211','0212','0213','0214','022' ,'0221','0222','0231'
,'0232','0233','0234','0235','0239','0241','0242','0243'
,'0291','0292','0293','0294','0295','0296','0299'


CREATE table neurosurgey as
select
distinct hadm_id
from
mimiciv_hosp.procedures_icd
where icd_code
in('00160KB','00160ZB','00163KB','00163ZB','00164KB','00164ZB','00500ZZ','00503ZZ'
,'00504ZZ','00510ZZ','00513ZZ','00514ZZ','00520ZZ','00523ZZ','00524ZZ','00560ZZ'
,'00563ZZ','00564ZZ','00570ZZ','00573ZZ','00574ZZ','00580ZZ','00583ZZ','00584ZZ'
,'00590ZZ','00593ZZ','00594ZZ','005A0ZZ','005A3ZZ','005A4ZZ','005B0ZZ','005B3ZZ'
,'005B4ZZ','005C0ZZ','005C3ZZ','005C4ZZ','005D0ZZ','005D3ZZ','005D4ZZ','00760ZZ'
,'00763ZZ','00764ZZ','00800ZZ','00803ZZ','00804ZZ','00870ZZ','00873ZZ','00874ZZ'
,'00880ZZ','00883ZZ','00884ZZ','009000Z','00900ZZ','009030Z','00903ZZ','009040Z'
,'00904ZZ','009100Z','00910ZZ','009130Z','00913ZZ','009140Z','00914ZZ','009200Z'
,'00920ZZ','009230Z','00923ZZ','009240Z','00924ZZ','009300Z','00930ZZ','009330Z'
,'00933ZZ','009340Z','00934ZZ','009400Z','00940ZZ','009430Z','00943ZZ','009440Z'
,'00944ZZ','009500Z','00950ZZ','009530Z','00953ZZ','009540Z','00954ZZ','009600Z'
,'00960ZZ','009630Z','00963ZZ','009640Z','00964ZZ','009700Z','00970ZZ','009730Z'
,'00973ZZ','009740Z','00974ZZ','009800Z','00980ZZ','009830Z','00983ZZ','009840Z'
,'00984ZZ','009900Z','00990ZZ','009930Z','00993ZZ','009940Z','00994ZZ','009A00Z'
,'009A0ZZ','009A30Z','009A3ZZ','009A40Z','009A4ZZ','009B00Z','009B0ZZ','009B30Z'
,'009B3ZZ','009B40Z','009B4ZZ','009C00Z','009C0ZZ','009C30Z','009C3ZZ','009C40Z'
,'009C4ZZ','009D00Z','009D0ZZ','009D30Z','009D3ZZ','009D40Z','009D4ZZ','00B00ZZ'
,'00B03ZZ','00B04ZZ','00B10ZZ','00B13ZZ','00B14ZZ','00B20ZZ','00B23ZZ','00B24ZZ'
,'00B60ZZ','00B63ZZ','00B64ZZ','00B70ZZ','00B73ZZ','00B74ZZ','00B80ZZ','00B83ZZ'
,'00B84ZZ','00B90ZZ','00B93ZZ','00B94ZZ','00BA0ZZ','00BA3ZZ','00BA4ZZ','00BB0ZZ'
,'00BB3ZZ','00BB4ZZ','00BC0ZZ','00BC3ZZ','00BC4ZZ','00BD0ZZ','00BD3ZZ','00C00ZZ'
,'00C03ZZ','00C04ZZ','00C10ZZ','00C13ZZ','00C14ZZ','00C20ZZ','00C23ZZ','00C24ZZ'
,'00C30ZZ','00C33ZZ','00C34ZZ','00C40ZZ','00C43ZZ','00C44ZZ','00C50ZZ','00C53ZZ'
,'00C54ZZ','00C60ZZ','00C63ZZ','00C64ZZ','00C70ZZ','00C73ZZ','00C74ZZ','00C80ZZ'
,'00C83ZZ','00C84ZZ','00C90ZZ','00C93ZZ','00C94ZZ','00CA0ZZ','00CA3ZZ','00CA4ZZ'
,'00CB0ZZ','00CB3ZZ','00CB4ZZ','00CC0ZZ','00CC3ZZ','00CC4ZZ','00CD0ZZ','00CD3ZZ'
,'00CD4ZZ','00D00ZZ','00D03ZZ','00D04ZZ','00D10ZZ','00D13ZZ','00D14ZZ','00D20ZZ'
```

,'00D23ZZ','00D24ZZ','00D70ZZ','00D73ZZ','00D74ZZ','00F30ZZ','00F33ZZ','00F34ZZ'
,'00F40ZZ','00F43ZZ','00F44ZZ','00F50ZZ','00F53ZZ','00F54ZZ','00F60ZZ','00F63ZZ'
,'00F64ZZ','00H002Z','00H003Z','00H004Z','00H00MZ','00H00YZ','00H032Z','00H033Z'
,'00H03MZ','00H03YZ','00H042Z','00H043Z','00H04MZ','00H04YZ','00H602Z','00H603Z'
,'00H60MZ','00H60YZ','00H632Z','00H633Z','00H63MZ','00H63YZ','00H642Z','00H643Z'
,'00H64MZ','00H64YZ','00J00ZZ','00J03ZZ','00J04ZZ','00K00ZZ','00K03ZZ','00K04ZZ'
,'00K70ZZ','00K73ZZ','00K74ZZ','00K80ZZ','00K83ZZ','00K84ZZ','00K90ZZ','00K93ZZ'
,'00K94ZZ','00KA0ZZ','00KA3ZZ','00KA4ZZ','00KB0ZZ','00KB3ZZ','00KB4ZZ','00KC0ZZ'
,'00KC3ZZ','00KC4ZZ','00KD0ZZ','00KD3ZZ','00KD4ZZ','00N00ZZ','00N03ZZ','00N04ZZ'
,'00N10ZZ','00N13ZZ','00N14ZZ','00N20ZZ','00N23ZZ','00N24ZZ','00N60ZZ','00N63ZZ'
,'00N64ZZ','00N70ZZ','00N73ZZ','00N74ZZ','00N80ZZ','00N83ZZ','00N84ZZ','00N90ZZ'
,'00N93ZZ','00N94ZZ','00NA0ZZ','00NA3ZZ','00NA4ZZ','00NB0ZZ','00NB3ZZ','00NB4ZZ'
,'00NC0ZZ','00NC3ZZ','00NC4ZZ','00ND0ZZ','00ND3ZZ','00ND4ZZ','00P000Z','00P002Z'
,'00P003Z','00P007Z','00P00JZ','00P00KZ','00P00MZ','00P00YZ','00P030Z','00P032Z'
,'00P033Z','00P037Z','00P03JZ','00P03KZ','00P03MZ','00P03YZ','00P040Z','00P042Z'
,'00P043Z','00P047Z','00P04JZ','00P04KZ','00P04MZ','00P04YZ','00P600Z','00P602Z'
,'00P603Z','00P60MZ','00P60YZ','00P630Z','00P632Z','00P633Z','00P63MZ','00P63YZ'
,'00P640Z','00P642Z','00P643Z','00P64MZ','00P64YZ','00Q00ZZ','00Q03ZZ','00Q04ZZ'
,'00Q10ZZ','00Q13ZZ','00Q14ZZ','00Q20ZZ','00Q23ZZ','00Q24ZZ','00Q60ZZ','00Q63ZZ'
,'00Q64ZZ','00Q70ZZ','00Q73ZZ','00Q74ZZ','00Q80ZZ','00Q83ZZ','00Q84ZZ','00Q90ZZ'
,'00Q93ZZ','00Q94ZZ','00QA0ZZ','00QA3ZZ','00QA4ZZ','00QB0ZZ','00QB3ZZ','00QB4ZZ'
,'00QC0ZZ','00QC3ZZ','00QC4ZZ','00QD0ZZ','00QD3ZZ','00QD4ZZ','00R107Z','00R10JZ'
,'00R10KZ','00R147Z','00R14JZ','00R14KZ','00R207Z','00R20JZ','00R20KZ','00R247Z'
,'00R24JZ','00R24KZ','00R607Z','00R60JZ','00R60KZ','00R647Z','00R64JZ','00R64KZ'
,'00T70ZZ','00T73ZZ','00T74ZZ','00U107Z','00U10JZ','00U10KZ','00U137Z','00U13JZ'
,'00U13KZ','00U147Z','00U14JZ','00U14KZ','00U207Z','00U20JZ','00U20KZ','00U237Z'
,'00U23JZ','00U23KZ','00U247Z','00U24JZ','00U24KZ','00U607Z','00U60JZ','00U60KZ'
,'00U637Z','00U63JZ','00U63KZ','00U647Z','00U64JZ','00U64KZ','00W000Z','00W002Z'
,'00W003Z','00W007Z','00W00JZ','00W00KZ','00W00MZ','00W00YZ','00W030Z','00W032Z'
,'00W033Z','00W037Z','00W03JZ','00W03KZ','00W03MZ','00W03YZ','00W040Z','00W042Z'
,'00W043Z','00W047Z','00W04JZ','00W04KZ','00W04MZ','00W04YZ','00W600Z','00W602Z'
,'00W603Z','00W60MZ','00W60YZ','00W630Z','00W632Z','00W633Z','00W63MZ','00W63YZ'
,'00W640Z','00W642Z','00W643Z','00W64MZ','00W64YZ','031G09G','031G0AG','031G0JG'
,'031G0KG','031G0ZG','031S09G','031S0AG','031S0JG','031S0KG','031S0ZG','031T09G'
,'031T0AG','031T0JG','031T0KG','031T0ZG','035G0ZZ','035G3ZZ','035G4ZZ','037G04Z'
,'037G05Z','037G06Z','037G07Z','037G0DZ','037G0EZ','037G0FZ','037G0GZ','037G0ZZ'
,'037G34Z','037G35Z','037G36Z','037G37Z','037G3DZ','037G3EZ','037G3FZ','037G3GZ'
,'037G3ZZ','037G44Z','037G45Z','037G46Z','037G47Z','037G4DZ','037G4EZ','037G4FZ'
,'037G4GZ','037G4ZZ','039G00Z','039G0ZZ','039G30Z','039G3ZZ','039G40Z','039G4ZZ'
,'03BG0ZZ','03BG3ZZ','03BG4ZZ','03CG0ZZ','03CG3Z7','03CG3ZZ','03CG4ZZ','03LG0BZ'
,'03LG0CZ','03LG0DZ','03LG0ZZ','03LG3CZ','03LG3ZZ','03LG4BZ','03LG4CZ','03LG4DZ'
,'03LG4ZZ','03RG07Z','03RG0JZ','03RG0KZ','03RG47Z','03RG4JZ','03RG4KZ','03UG07Z'
,'03UG0JZ','03UG0KZ','03UG37Z','03UG3JZ','03UG3KZ','03UG47Z','03UG4JZ','03UG4KZ'

```
,'03VG0BZ','03VG0CZ','03VG0DZ','03VG0HZ','03VG0ZZ','03VG3BZ','03VG3CZ','03VG3DZ'
,'03VG3HZ','03VG3ZZ','03VG4BZ','03VG4CZ','03VG4DZ','03VG4HZ','03VG4ZZ','055L0ZZ'
,'055L3ZZ','055L4ZZ','05BL0ZZ','05BL3ZZ','05BL4ZZ','05LL0CZ','05LL0DZ','05LL0ZZ'
,'05LL3CZ','05LL3ZZ','05LL4CZ','05LL4DZ','05LL4ZZ','05RL07Z','05RL0JZ','05RL0KZ'
,'05RL47Z','05RL4JZ','05RL4KZ','0G500ZZ','0G503ZZ','0G504ZZ','0G510ZZ','0G513ZZ'
,'0G514ZZ','0G800ZZ','0G803ZZ','0G804ZZ','0G9000Z','0G900ZZ','0G9030Z','0G903ZZ'
,'0G9040Z','0G904ZZ','0G9100Z','0G910ZZ','0G9130Z','0G913ZZ','0G9140Z','0G914ZZ'
,'0GB00ZZ','0GB03ZZ','0GB04ZZ','0GB10ZZ','0GB13ZZ','0GB14ZZ','0GC00ZZ','0GC03ZZ'
,'0GC04ZZ','0GC10ZZ','0GC13ZZ','0GC14ZZ','0GJ00ZZ','0GJ03ZZ','0GJ04ZZ','0GJ10ZZ'
,'0GJ13ZZ','0GJ14ZZ','0GN00ZZ','0GN03ZZ','0GN04ZZ','0GN10ZZ','0GN13ZZ','0GN14ZZ'
,'0GP000Z','0GP030Z','0GP040Z','0GP100Z','0GP130Z','0GP140Z','0GQ00ZZ','0GQ03ZZ'
,'0GQ04ZZ','0GQ10ZZ','0GQ13ZZ','0GQ14ZZ','0GT00ZZ','0GT04ZZ','0GT10ZZ','0GT14ZZ'
,'0GW000Z','0GW030Z','0GW040Z','0GW100Z','0GW130Z','0GW140Z','0W110J9','0W110JB'
,'0W110JG','0W110JJ','0W9100Z','0W910ZZ','0W9130Z','0W913ZZ','0W9140Z','0W914ZZ'
,'0WC10ZZ','0WC13ZZ','0WC14ZZ','0WF10ZZ','0WF13ZZ','0WF14ZZ','0WH101Z','0WH103Z'
,'0WH10YZ','0WH131Z','0WH133Z','0WH13YZ','0WH141Z','0WH143Z','0WH14YZ','0WJ10ZZ'
,'0WJ13ZZ','0WJ14ZZ','0WP100Z','0WP101Z','0WP103Z','0WP10JZ','0WP10YZ','0WP130Z'
,'0WP131Z','0WP133Z','0WP13JZ','0WP13YZ','0WP140Z','0WP141Z','0WP143Z','0WP14JZ'
,'0WP14YZ','0WW100Z','0WW101Z','0WW103Z','0WW10JZ','0WW10YZ','0WW130Z','0WW131Z'
,'0WW133Z','0WW13JZ','0WW13YZ','0WW140Z','0WW141Z','0WW143Z','0WW14JZ','0WW14YZ'
,'3E0Q004','3E0Q005','3E0Q00M','3E0Q028','3E0Q029','3E0Q03Z','3E0Q06Z','3E0Q07Z'
,'3E0Q0AZ','3E0Q0BZ','3E0Q0E0','3E0Q0E1','3E0Q0GC','3E0Q0HZ','3E0Q0KZ','3E0Q0NZ'
,'3E0Q0SF','3E0Q0TZ','0101','0102','0109','0110','0111','0112','0113','0114'
,'0115','0116','0117','0118','0119','0120','0121','0122'
,'0123','0124','0125','0126','0127','0128','0129','0131'
,'0132','0139','0141','0142','0151','0152','0153','0159'
,'016' ,'0201','0202','0203','0204','0205','0206','0207'
,'0211','0212','0213','0214','022' ,'0221','0222','0231'
,'0232','0233','0234','0235','0239','0241','0242','0243'
,'0291','0292','0293','0294','0295','0296','0299')


create table normal_patients as
select
mha.subject_id
,mha.hadm_id
,mda.age
,case when mhp.gender = 'M' then 1
        else 0
        end as gender
,mha.hospital_expire_flag as flag
from   mimiciv_hosp.admissions mha,mimiciv_derived.age mda, mimiciv_hosp.patients mhp
where mda.hadm_id= mha.hadm_id
```

```
  and mhp.subject_id = mha.subject_id
  and mha.dischtime >= DATETIME_ADD(mha.admittime, INTERVAL '1' DAY)
  order by mha.subject_id ,mha.hadm_id


CREATE TABLE phosphate AS
SELECT
    MAX(subject_id) AS subject_id
  , MAX(hadm_id) AS hadm_id
  , MAX(charttime) AS charttime
  , le.specimen_id
  -- convert from itemid into a meaningful column
  , MAX(CASE WHEN itemid = 50970 THEN valuenum ELSE NULL END) AS phosphate
FROM mimiciv_hosp.labevents le
WHERE le.itemid = 50970
AND valuenum IS NOT NULL
-- lab values cannot be 0 and cannot be negative
AND valuenum > 0
---AND valuenum <> '__'
AND hadm_id IS NOT NULL
GROUP BY le.specimen_id
;

,CASE when dod is null then 1
      else case when datetime_diff(dod, CAST(admittime AS DATE), 'DAY')/28 < 1 then
datetime_diff(dod, CAST(admittime AS DATE), 'DAY')/28
              when datetime_diff(dod, CAST(admittime AS DATE), 'DAY')/28 >= 1 then 1
              else null end
      end as onemouthsurvival

,CASE when dod is null then 3
      else case when datetime_diff(dod, CAST(admittime AS DATE), 'DAY')/28 < 3 then
datetime_diff(dod, CAST(admittime AS DATE), 'DAY')/28
              when datetime_diff(dod, CAST(admittime AS DATE), 'DAY')/28 >= 3 then 3
              else null end
      end as threemouthsurvival

,CASE when dod is null then 12
      else case when datetime_diff(dod, CAST(admittime AS DATE), 'DAY')/28 < 12 then
datetime_diff(dod, CAST(admittime AS DATE), 'DAY')/28
              when datetime_diff(dod, CAST(admittime AS DATE), 'DAY')/28 >= 12 then 12
              else null end
      end as oneyearsurvival
```
